# Efficient prediction of temperature-dependent elastic and mechanical properties of 2D materials

**DOI:** 10.1038/s41598-022-07819-8

**Published:** 2022-03-08

**Authors:** S. M. Kastuar, C. E. Ekuma, Z. -L. Liu

**Affiliations:** 1 Department of Physics, Lehigh University, Bethlehem, PA 18015 USA; 2 School of Materials Science and Engineering, Harbin Institute of Technology, Harbin, China; 3 College of Physics and Electric Information, Luoyang Normal University, Luoyang, 471934 China

**Keywords:** Mathematics and computing, Physics, Condensed-matter physics, Structure of solids and liquids, Materials science, Nanoscale materials, Structural materials, Theory and computation

## Abstract

An efficient automated toolkit for predicting the mechanical properties of materials can accelerate new materials design and discovery; this process often involves screening large configurational space in high-throughput calculations. Herein, we present the ElasTool toolkit for these applications. In particular, we use the ElasTool to study diversity of 2D materials and heterostructures including their temperature-dependent mechanical properties, and developed a machine learning algorithm for exploring predicted properties.

## Introduction

The mechanical and elastic properties of materials are among the most fundamental properties that must be accurately determined and are essential in many disciplines not limited to condensed matter physics, materials science, and geophysics^1,2^. In new materials design and integration into devices, mechanical properties, and elastic constants are frequently used to determine the stability of the structures^3,4^. The fundamental challenge in creating such new materials includes (1) the ability to enumerate the compositional space that is often too large for an Edisonian approach, and (2) rapidly characterize the thermodynamic, electronic, and optical properties. To facilitate this process, there is a growing need to develop an automated computational toolkit for predicting essential properties such as the elastic and mechanical properties of bulk as well as 2D materials from first-principles elastic tensor calculations^5,6^. Moreover, the accurate knowledge of the full elastic tensor enables the determination of many other important elastic, mechanical, and thermodynamic properties of materials that are essential for screening, design, and discovery of new materials^7,8^.

**Figure 1:**
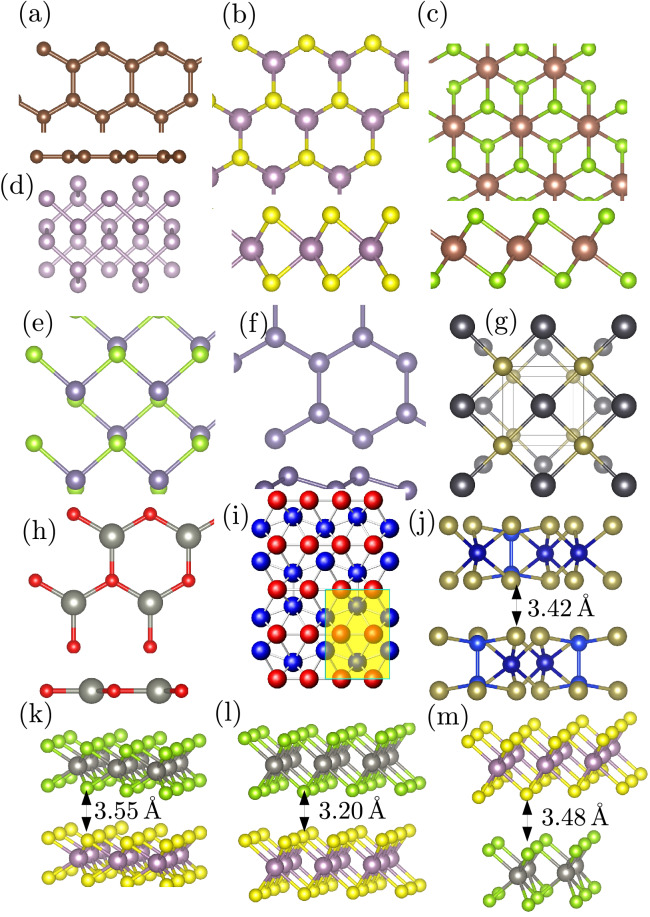
Crystal structure of diversity of 2D-based materials and their heterostructures. (**a**) Top and side view of the hexagonal structure of graphene. (**b**) Top and side view of the trigonal prismatic crystal structure of 2H transition metal dichalcogenides (TMDs) such as MoS$_2$. (**c**) Top and side view of the octahedral 1T-TMDs structures such as WS$_2$ and post-transition metal chalcogenides such as SnS$_2$. (**d**) Simple orthorhombic (Pmna, space group 53) structure of phosphorene. (**e**) Orthorhombic (Pmn2$_1$, space group 7) structure of group IV monochalcogenides, e.g., GeSe. (**f**) Top and side view of the silicene-like structures. (**g**) Tetragonal crystal structure of group IV–VI monochalcogenides such as PbTe, (**h**) crystal structure of the IV–VI materials, e.g., ZnS, (**i**) 8-Pmmn orthorhombic crystal structure (highlighted area depicts the unit cell) of borophene. Blue (inner position atoms) and red (atoms along the ridges) depict the nonequivalent boron atoms. (**j**) Trigonal crystal structure of transition metal trichalcogenides $\mathrm {ABX_3}$, where A $=\,$V, Cr, Mn, Fe, Co, Ni, and Cu; B $=\,$Si and Ge; and X $=\,$S, Se, and Te. (**k**–**m**) are the crystal structures of 2H, 1T, and 2H-1T heterostructures designed with MoS$_2$ and WS$_2$, respectively.

Recently, and specifically for 2D-based materials (Fig. 1), high-throughput elastic properties of several materials have been calculated and collected in some materials’ databases such as the Materials Project, the JARVIS-DFT, and the Computational 2D Materials Database^5,7,9^. The ElasTool toolkit^10,11^ builds upon the essential benefits of these already developed methods. However, unlike the existing toolkit for computing the elastic and mechanical properties of materials, the ElasTool toolkit can automate the process of computing zero-temperature as well as temperature-dependent elastic properties. It provides a complete elastic and mechanical toolkit for both automation and traditional prediction of zero-temperature, temperature-dependent, and the combined impact of dynamical pressure and temperature on crystal systems.

**Figure 2:**
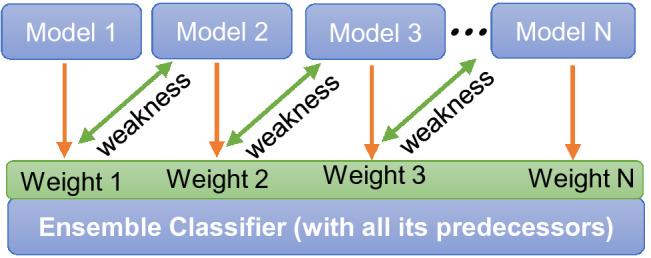
Schematic of ML boosting model. Model 1, 2, ..., *N* consisting of parallel learners and weighted dataset—one is weak (just as in standard algorithm), however, when they band together they are strong and together, they learn from the past.

The zero-temperature elastic and mechanical properties of several 2D-based materials, including their heterostructures and the less studied trichalcogenides, have been computed (see Table S3 in the Supplementary Material)^12^. The predicted elastic and mechanical properties are in excellent agreement with existing data in literature^5,8,13–16^. For example, our predicted Y$^{2D}\sim$135.86 N/m is in good agreement with recent experiments, which span from 129 to 185 N/m^15,17–19^ and previous first-principles calculations that reported values 122–146 N/m^20^. Our calculations show that most of the 1T-TMDs, e.g., 1T-MoSe$_2$ exhibit an auxetic behavior, manifested by the negative Poisson ratio. This behavior has been observed in previous studies^21^. It was attributed to the metastability of the 1T-TMDs mediated by the strong coupling between the chalcogen dominated *p*-states and the intermetallic $t_{2g}$-bonding states within the basic triangular pyramid building block structure. Indeed, as shown in Table S4 (see Supplementary Material (SM)^12^), the application of temperature of $\sim$300 K stabilizes the auxetic states. Temperature-induced structural distortions in the 1T structure (see right panel of Fig. 2) leads to a pressure of $\sim 2.41$ N/m (note the optimized vacuum size is 21.50 Å), which stabilized the structure. We also computed the elastic and mechanical properties of the less studied family of the transition metal trichalcogenides (ABX$_3$) with space C3i (No. 147), where A $=\,$V, Cr, Mn, Fe, Co, Ni, and Cu; B $=\,$Si and Ge; and X $=\,$S, Se, and Te. They form sandwich layers of X-(A,B)-X stacks. Each of the B atoms possesses three neighboring X atoms forming a tetrahedron, and two of the B-centered tetrahedrons forming a dumbbell-like B dimer in [AX$_6$]$^{4-}$ bipyramid leading to a monolayer unit cell composed of two A$^{2+}$ and one [AX$_6$]$^{4-}$ ions. The structure is such that in-plane interactions are dominated by strong covalent bonds while interlayers are mediated by weak van der Waals interactions. The trichalcogenides, especially those with the 3*d* end members, seem more stable with a spin-polarized solution. We note that even though CrSiS$_3$ shows instability, spin-polarized solution stabilizes the mechanical properties with K $\sim 196.43$ N/m, G $\sim 106.32$ N/m, Y$^{2D}\sim 275.99$ N/m.

Hardness is a fundamental property of a material that plays an essential role in the full description of the mechanical properties of materials. To gain a fundamental understanding of the macroscopic mechanical properties of materials under deformation, the ideal strength, which is the minimum stress needed to plastically deform a material vis-à-vis the upper bound to the critical stress for dislocation and crack nucleation in a material can in principle be calculated. However, for high-throughput calculations in materials screening and new materials design, such calculations can be highly challenging due to the large configurational space. We can estimate the ideal strength based on the modification of empirical relations between elastic moduli and the Vickers hardness H$_v$ proposed for bulk crystals^22,23^. Specific for 2D materials, we have the following empirical relations: $H_v^{1a}=0.151 G$, $H_v^{1b}=0.0608 Y^{2D}$, $H_v^{1c}=0.0963 K$, $H_v^{2}=0.1769 G-2.899$, $H_v^{3}=(1-2\nu )K/(6+6\nu )$, $H_v^{4}=2(G^3/K^2)^{0.585}-3$. To determine the best empirical model, we compute the hardness of the various 2D materials and heterostructures. Our test shows that all the models perform well for the superhard and moderately hard materials such as graphene, hexagonal BN, and borophene. For example, the $H_v$ for graphene using the models is $\sim$ 21 ± 2 N/m and $H_v\sim 16\pm 1$ for hexagonal BN, which is in good agreement with experiments if we use a thickness of approximately 3.35 Å^24,25^. However, aside from the superhard and moderately hard materials, our calculations show that models $H_v^{1a-1c}$ are robust enough to predict the hardness of the diversity of 2D materials and heterostructures. Specifically for the trichalcogenides, models $H_v^{1a-1b}$ are the most appropriate. In all cases, models $H_v^{2-4}$ are highly unreliable with very large uncertainties and should be avoided for 2D-based materials.

**Figure 3:**
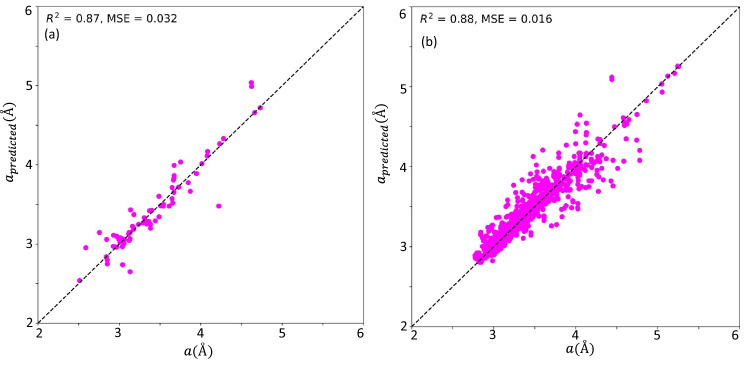
The relation between the computed lattice constants from our first-principles calculations and the predicted lattice constants from our machine learning model for the out-of-sample (unseen) data for (**a**) 2D materials and (**b**) 2D-based heterostructures. In both 2D and the heterostructures, computed accuracy (R$^2$) score is basically the same.

Our database currently has over 6000 computed temperature-dependent elastic and mechanical properties of 2D crystals and their heterostructures. With such a highly accurate and vast dataset, we can develop a machine-learning algorithm to gain a deeper understanding of the relationship between the lattice parameters and the associated mechanical and elastic properties. Machine learning (ML) offers a burgeoning approach in diverse fields and could assist to unravel hidden structure-property relations. (see all the details and other accompanying figures in the Supplementary Material). Aside from data integrity, the accuracy of any ML-based model depends on the set of features used in the training model. The choice of the target and features is nontrivial. We contemplated a number of potential targets and features. We initially used Young’s modulus, but since it is directly correlated with the elastic tensors, we had to drop it. The energy bandgap is often a good choice, but obtaining a highly accurate bandgap, for developing a robust ML algorithm beyond the training dataset is highly challenging. Accurate bandgap, especially for 2D-based structures where many-body effects due to quantum confinement dominate will require approaches such as the Green’s function and screened Coulomb method or at most the hybrid functional. In the end, we settled for the lattice constant *a* as the target. This choice is due to the important role it plays in determining the elastic and mechanical properties of materials. For the features, we have used the *Temp* (i.e., temperature), *SG* (i.e., space group), $C_{11}$, $C_{12}$, and *c* (i.e., vacuum size). As explained in detail in the SM^12^, we have restricted our choice of the 2D-based materials and heterostructures to isotropic lattices, i.e., crystal structures where the lattice constants *a* and *b* are equal. Unlike several other calculations for 2D-based materials where the vacuum size is a randomly fixed parameter, we have instead self-consistently determined it for each material. This is important since *c* in 2D-based materials plays a significant role not only to avoid artifacts of periodic boundary conditions, but has been shown to impact its properties. A rigorous exploration of the various regression models showed that the boosting models—XGBoost and LightGBM had the overall best performance. A boosting ML model is basically a generic algorithm (rather than a specific model); it seeks to improve the prediction power of the ML algorithm by systematically training a set of weak models (learners) with each compensating the weakness of its predecessor, thereby reducing bias and variance in predictions (Fig. 2). In this regard, the results presented herein are based on the XGBoost, which shows slightly better performance than the LightGBM (see accuracy and error metrics summarized in Table S2).

We also determined the contribution of each of the features to the ML model. Initial exploratory data analysis showed that the above features are the most significant. Temperature is found to contribute $\sim$ 20% and 6% for 2D-based materials and 2D-based heterostructures, respectively in determining the lattice constant. Vacuum size is found to play a significant role as well, especially for the 2D-based heterostructures, where $\sim$ 58% of the model feature importance are due to *c* (Fig. S4). This necessitated our choice of self-consistently calculating the vacuum size for each material. A more detailed exploratory study to unravel how the feature importance evolves in both 2D materials and 2D-based heterostructures will be a study of future research.

The developed ML algorithm showed consistent accuracy scores and error metrics (Fig. S3) both in the training, test, and out-of-sample datasets. Details are presented in the SM^12^ and a step-by-step guide of the implementation is provided in the accompanying manuscript code. Here, we want to focus on the performance of the developed ML model when used in out-of-sample (unseen) data, i.e., predictions on a dataset that is not part of both the training and testing samples. As presented in Fig. 3 and Fig. S5, the accuracy of the developed model is close to 90% for both the 2D-based materials and 2D-based heterostructures. The lowest percentage error is 0.26% and the highest percentage error is $\sim$ 7%. This is significant in two folds. The performance of the developed ML algorithm when used in out-of-sample data is basically the same as the accuracy score and error metrics from the cross-validation analysis. Secondly, we believe that the performance is within and in most cases, better than the standard acceptable error for 2D-based lattice constants. It is well-known that the lattice constant of 2D-based materials can span up to 27% depending on the type of functional used and other input parameters. For example, the lattice constant of MoS$_2$ can span from 3.16 Å to as high as 3.30 Å. From the analysis of the feature importance in the ML model (Fig. S4), the vacuum size plays an essential role, especially for the 2D-based heterostructures in determining the lattice constant.

## Summary

To summarize, we present an efficient computational toolkit based on density functional theory and ab-initio molecular dynamics to automate the calculation of the elastic and mechanical properties of several 2D materials and their heterostructures at both zero- and finite-temperature. A machine learning algorithm is developed for exploring the relation between the predicted properties. We believe that the highly efficient and automation enabled by the ElasTool toolkit will be important in both computational materials screening and traditional prediction of the mechanical and related properties of materials at experimentally relevant conditions such as temperature, pressure, or the combination of both that is not currently a routine in many electronic structure codes.

## Method

### Theoretical background

The stress–strain and the strain-energy method are the two essential approaches to compute the elastic properties of materials^26,27^. While the stress–strain approach is computationally more expensive, it is, however, simpler to implement and does not require the complicated pressure corrections needed for the strain-energy method when computing high-pressure elastic constants of materials^28^. Under a small and homogeneous deformation $\mathbf {D}=\mathcal {I}+\xi$, where $\mathcal {I}$ is a $3\times 3$ unit matrix. Within the elastic limit, the generalized stress–strain Hooke’s law is $\sigma _{ij}=C_{ijkl}\xi _{kl}$, where $C_{ijkl}$ is a fourth-rank elastic tensor and $\sigma _{ij}$ ($\xi _{kl}$) is a second-rank stress (strain) tensor. In Voigt notation, the stress–strain relation is

1 $$\begin{aligned} \sigma _i = \sum _{j=1}^6 C_{ij} \xi _j \end{aligned}$$

For 2D materials, only the in-plane elastic matrix is essential^14,29^

2 $$\begin{aligned} \begin{bmatrix} \sigma _1 \\ \sigma _2 \\ \sigma _6 \end{bmatrix} = \begin{bmatrix} C_{11} &{} C_{12} &{} 0 \\ C_{12} &{} C_{22} &{} 0 \\ 0 &{} 0 &{} C_{66} \end{bmatrix} \begin{bmatrix} \xi _1 \\ \xi _2 \\ \xi _6 \end{bmatrix} \end{aligned}$$

where $C_{66}=(C_{ii}-C_{ij})/2$, $i=1,2$. After deformation, the crystal lattice vector can be obtained as $\mathbf {A^\prime = A.D}$, where $\mathbf {A}$ is the undeformed crystal lattice vector. The elastic constants can readily be obtained by a polynomial fit of Eq. (2). Having obtained the elastic constants, we can compute other related properties. The in-plane arrangement of 2D-material designates the atomic arrangement in an *x*–*y* plane of chirality angle $\theta$ that span between 0$^{\circ }$ and 30$^{\circ }$, where $\theta =0^{\circ }$ and $30^{\circ }$ are for the zigzag and armchair chirality, respectively. For any arbitrary angle, the in-plane (2D) Young’s modulus Y$^{2D}$ and the Poisson’s ratio $\nu$ are^30^

3 $$\begin{aligned}&Y^{2D}(\theta ) = \frac{C_{11}C_{22} - C_{12}^2 }{C_{11}s^4 +C_{22}c^4 +c^2s^2\gamma } \end{aligned}$$

4 $$\begin{aligned}&\nu (\theta ) = \frac{C_{11}C_{22} - \frac{C_{11}C_{22} -C_{12}^2 }{C_{66}}c^2s^2 -C_{12}(c^4+s^4) }{C_{11}s^4 +C_{22}c^4 +c^2s^2\gamma } \end{aligned}$$

where $\gamma = [(C_{11}C_{22}-C_{12^2} )/C_{66}-2C_{12}]c^2s^2$, $c=\cos \,\theta$, and $s=\sin \,\theta$. Assuming $\theta =0$, then

5 $$\begin{aligned} Y^{2D} = \frac{C_{ii}^2 -C_{ij}^2 }{C_{ii}};\quad \nu = \frac{C_{ij}}{C_{ii}}\,\quad ; i,j=1,2 \end{aligned}$$

Having obtained the Young’s modulus and the Poisson ratio, we can compute the in-plane stiffness (layer modulus) *K*, i.e., the 2D equivalent of the bulk modulus and the shear modulus G as^31^

6 $$\begin{aligned} K = \frac{Y^{2D}}{2(1-\nu )}\,;\quad G = \frac{Y^{2D}}{2(1+\nu )}. \end{aligned}$$

The bulk modulus measures the resistance of a bulk material to compression while the layer modulus represents the resistance of a 2D material to stretching. Stiffness is a measure of the resistance of a material to elastic deformation. We can use it to measure the sound velocity in crystals [62,63] as

7 $$\begin{aligned} V_l = \sqrt{\frac{K + G}{\rho _{2D}}}\,;\quad V_t = \sqrt{\frac{G}{\rho _{2D}}} \end{aligned}$$

where $V_l$ and $V_t$ are the longitudinal and shear sound velocity, respectively, and $\rho _{2D}$ is the 2D mass density. We note that other forms of the sound velocities exist in literature, e.g., $V_t=\sqrt{C_{12}/\rho _{2D}}$^32^. Having obtained the sound velocities, it is straightforward to compute the Debye temperature $\Theta _D$ from the average sound velocities $V_a$ as^33^

8 $$\begin{aligned} \Theta _D = \frac{\hbar V_a}{\kappa _B} \left( \frac{4\pi N}{S}\right) ^{1/2} \end{aligned}$$

where $\hbar$ is the reduced Planck constant, *N* is the number of atoms in the unit cell, $\kappa _B$ is the Boltzmann constant, *S* is the area of the unit cell, and $V_a$ for a typical 2D system is

9 $$\begin{aligned} V_a = \left[ \frac{1}{2} \left( \frac{1}{V_l^2} + \frac{1}{V_t^2}\right) \right] ^{-1/2} \end{aligned}$$

**Figure 4:**
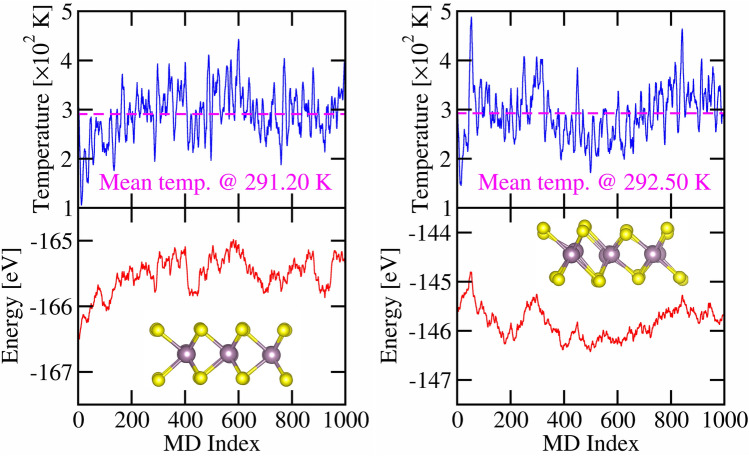
A representative profile of temperature and the total energy at various molecular dynamics steps. Left panel is for 2H-MoS$_2$ and right panel is for 1T-MoS$_2$. The inset in both plots is the crystal structure at the simulated temperature of 300 K. The applied temperature induced 0.0 (2.41 N/m) pressure on the 2H (1T) MoS$_2$ crystal structures.

### Code structure

The ElasTool toolkit is based on Python and can be downloaded from GitHub^10^. It exploits the versatility of several Python-based libraries such as NumPy for numerical calculations, ASE for structure manipulations, Pandas for efficient statistics of stress tensor calculations, and Spglib for automatic determination of the crystal structure symmetry. These libraries are easily installed via pip or conda. Currently, the ElasTool is interfaced with the VASP electronic structure code; interfacing with other electronic structure codes is straightforward. The main input file named “elastool.in” is needed to run the code. The crystal information is provided in the standard crystal information format or the POSCAR format, which is the standard form for VASP code. Other key input parameters are self explanatory and require no technical knowledge. Since the ElasTool code is currently interfaced with VASP, various key files namely INCARs for structural optimization and stress tensor calculations, KPOINTS-static for zero-temperature calculations, KPOINTS-dynamic for temperature-dependent calculations using ab initio molecular dynamics are provided. While the code usage is easy and straightforward, we have also provided several example calculations in the example folder. Other details of the ElasTool toolkit are provided in the Supplementary Material^12^ and in the documentation provided on the code website at https://github.com/zhongliliu/elastool.

### Computational details

The ElasTool toolkit obtains the second-order elastic constants of any material using highly optimized, high-efficient strain-matrix sets. It currently uses the VASP code^34^ as the stress–strain calculator. Extension to other electronic structure codes is straightforward. All the calculations were done within the first-principles density functional theory^35,36^ using the Perdew-Burke-Ernzerhof (PBE)^37^ exchange-correlation functional. The $\mathbf{k}$-point sampling was done on a $15\times \ 15 \times 1$ $\Gamma$-center grid. The total energy (charge) is converged to within 10$^{-7}$ (10$^{-3}$) eV, with the residual stresses and forces less than 0.01 N/m and 10$^{-3}$ eV/Å using a Fermi distribution function with a smearing parameter of 0.10 eV to integrate the states at the Fermi level. In all the cases, a kinetic energy cutoff of 550 eV was used. All calculations included van der Waals interaction corrections to avoid spurious interactions between the periodically repeated images of the slab. We used a vacuum of at least 15 Å along the out-of-plane direction to further eliminate the artifacts of the periodic boundary condition. The temperature-dependent elastic and mechanical properties were obtained using the thermal stress–strain data from ab initio molecular dynamics (AIMD)^38^ as implemented in VASP^34^. These calculations employed $5\times \ 5 \times 1$ $\Gamma$-center grid to sample the Brillouin zone. AIMD provides a reliable description of the time-evolution of systems and often reveals non-intuitive temperature-dependent system configurations. The structures were initially optimized with the PBE exchange-correlation functional and then equilibrated under an isothermal isobaric (NPT) ensemble using the Langevin thermostat to maintain the temperature. The structures were further simulated under a canonical (NVT) ensemble using the Nose-Hoover thermostat to maintain the temperature. All calculations used cutoff energy of 550 eV, a time step of 2 fs, and 1000 molecular dynamics (MD) steps with the last 500 MD steps used to average the thermal stresses. A representative MD profile of the temperature of the total energy for 2H- and 1T-MoS$_2$ are shown in Fig. 4.

## Supplementary Information

Supplementary Information.

## Data Availability

All data produced and analyzed in the this study are included in this published article. Additionally, we have computed the elastic and mechanical properties of over ten thousand 2D materials and their heterostructures. We will continue to update the database and the corresponding machine learning algorithm in the GitHub repository https://github.com/gmp007/2D_Elastic-Properties. Additional correspondence and request can be made by email to the authors.

## Code availability

The ElasTool is available free of charge on GitHub^10^. Developmental version can also be requested directly by emailing the authors.
