# Supplementary material for: Efficient prediction of temperature-dependent elastic and mechanical properties of 2D materials
Source: Sci Rep. 2022 Mar 8;12:3776. doi: 10.1038/s41598-022-07819-8 (PMC8904584; doi:10.1038/s41598-022-07819-8)
Supplement: Supplementary file 1 — Supplementary Information. [file 41598_2022_7819_MOESM1_ESM.pdf]

Supplementary Materials: Efficient prediction of temperature-dependent elastic and mechanical properties of 2D materials

S. M. Kastuar,[1] C. E. Ekuma,[1,*] and Z. -L. Liu[2,3]

[1]*Department of Physics, Lehigh University, Bethlehem, PA 18015, USA*
[2]*School of Materials Science and Engineering, Harbin Institute of Technology, Harbin, China*
[3]*College of Physics and Electric Information, Luoyang Normal University, Luoyang 471934, China*
(Dated: December 3, 2021)

ELASTOOL INPUT AND FLOWCHAT

The main input file for the ElasTool toolkit is the file named `elastool.in`; the key parameters are given in Table S1 and described below. We have also provided a sample `elastool.in` file for a typical 2D material in Input 1. The main input parameters in a typical `elastool.in` are described as follow.

(a) run_mode controls the type or level of calculations we want to perform. run_mode = 1 is for automatic run, i.e., the calculations starts from reading the crystal information and finishes by outputting the elastic tensor and related mechanical properties; run_mode = 2 should be used if the structure has been optimized at fixed volume or pressure with the subdirectory OPT containing the CONTCAR and the OUTCAR file; and run_mode = 3 is a post-processing option that enables recalculation of the elastic parameters. The subfolder STRESS that contains the various calculations at various strains must exist.

(b) dimensional determines the dimension of the crystal system. Currently, ElasTool can perform elastic constant calculations for both 2D and 3D materials.

(c) structure$_f$ile supplies the crystal structure information of the material in either the standard VASP POSCAR file or CIF with the extensions ".vasp" and ".cif", respectively.

(d) method_stress_statistics sets either zero-temperature (denoted as static) or finite-temperature (denoted with dynamic) calculations.

(e) strains_matrix enables choosing the method to be used for computing the stresses. The high-efficiency strain-matrix sets (OHESS) is our most efficient method.

(f) strains_list is used to specify the strain values for the calculations, e.g., for a static calculations, one can use -0.06 -0.03 0.03 0.06. For finite-temperature calculations, because of the computational cost, the strain list can be -0.03 0.03.

(g) repeat_num is used to specify the size of the supercell, e.g., $3 \times 3 \times 1$. This flag is only used for finite-temperature calculations. It is a dummy variable for zero temperature calculations.

(h) num_last_samples is used to specify the the number of last molecular dynamics (MD) steps to average thermal stresses. A good number is 500 for a 1000 MD steps. This flag is dormant for zero temperature calculations.

(i) parallel_submit_command is used to specify how the VASP executable is called. This can take serial as well as parallel calls to the VASP code.

TABLE S1. The controlling parameters and possible values for the main input file, elastol.in of ElasTool

Parameters	Values
`run_mode`	`1/2/3`
`dimensional`	`2D/3D`
`structure_file`	`name ends with .vasp or .cif`
`if_conventional_cell`	`yes/no`
`method_stress_statistics`	`static/dynamic`
`strains_matrix`	`ohess/asess/ulics`
`strains_list`	`one or more numbers`
`repeat_num`	`3 integers`
`num_last_samples`	`1 integer`
`parallel_submit_command`	`DFT parallel run command`

Input 1. A sample elastol.in file for 2D materials calculations

```
# run mode: 1 for automatic run,
# 2 for pre-processing,
# 3 for post-processing
run_mode = 1

# Define the dimensional of the system: 2D/3D
dimensional = 2D

# Crystal structure file in
# vasp POSCAR (.vasp) or cif (.cif) format
structure_file = CONTCAR.vasp

# if use conventional cell,
# no for primitive cell,
# yes for conventional cell
if_conventional_cell = yes

# static or dynamic, static for 0 K,
# dynamic for finite-temperature
method_stress_statistics = static

# strains matrix for
# solving all elastic constants,
```

```
# asess or ohess or ulics
strains_matrix = ohess

# strains list for deforming lattice cell,
strains_list = -0.06 -0.03 0.03 0.06

# repeat numbers of three lattice vectors
# in conventional lattice for making
# supercell of MD simulations
repeat_num = 3 3 1

# last number of steps for sampling
# stresses used in the dynamic method
num_last_samples = 500

# The parallel submiting commmd
parallel_submit_command = aprun
-n 44 vasp_std > log.vasp
```

An auxiliary input file named INCARs are provided as well. This file supplies all the necessary VASP-specific input parameters and flags need to optimize the structure and compute the stresses with either density functional theory for zero-temperature (Input 2) and *ab initio* molecular dynamics for finite-temperature calculations (Input 3). The given INCARs are for the calculation of a typical 2D material with two atoms per unit cell, e.g., MoS_2.

Input 2. INCARs for zero-temperature calculations

```
##—— Step: fixed-pressure-opt ——##
PREC     = Accurate
ENCUT    = 550
EDIFF    = 1e-5
EDIFFG   = -0.02
ISPIN    = 1
IBRION   = 2
ISIF     = 4
ISYM     = 2
NSW      = 200
ISMEAR   = 0
SIGMA    = 0.1
POTIM    = 0.1
PSTRESS  = 0.001 # Can be removed
NPAR     = 4
NSIM     = 4
ALGO     = Normal
IALGO    = 48
ISTART   = 0
LPLANE   = .TRUE.
LCHARG   = .FALSE.
LWAVE    = .FALSE.
LVDW     = .TRUE. #ADD VDW INTERACTION
IVDW     = 12 # DFT-D3

##—— Step: fixed-volume-opt ——##
PREC     = Accurate
ENCUT    = 550
EDIFF    = 1e-5
ISPIN=1
IBRION   = 2
ISIF     = 2
ISYM     = 2
NSW      = 200
ISMEAR   = 0
SIGMA    = 0.1
POTIM    = 0.1

NPAR     = 4
NSIM     = 4
ALGO     = Normal
IALGO    = 48
ISTART   = 0
LPLANE   = .TRUE.
LCHARG   = .FALSE.
LWAVE    = .FALSE.
LVDW = .TRUE. # ADD VDW INTERACTION
IVDW =12 # DFT-D3
```

Input 3. INCARs for finite-temperature calculations

```
##—— Step: fixed-pressure-opt ——##
PREC     = Accurate
ENCUT    = 550
EDIFF    = 1e-4
EDIFFG   = -0.001
ISPIN=1
IBRION   = 2
ISIF     = 4
ISYM     = 2
NSW      = 100
ISMEAR   = 2
SIGMA    = 0.2
POTIM    = 0.1
PSTRESS  = 0.0001
NPAR     = 4
NSIM     = 4
ALGO     = Normal
IALGO    = 48
ISTART   = 0
LPLANE   = .TRUE.
LCHARG   = .FALSE.
LWAVE    = .FALSE.
IWAVPR   = 11
LVDW = .TRUE. # ADD VDW INTERACTION
IVDW =12
##—— Step: fixed-volume-opt ——##
PREC     = Accurate
ENCUT    = 550
EDIFF    = 1e-4
EDIFFG   = -0.001
ISPIN      = 1
IBRION   = 2
ISIF     = 2
ISYM     = 2
NSW      = 2000
ISMEAR   = 2
SIGMA    = 0.2
```

```
POTIM    = 0.1

NPAR     = 4
NSIM     = 4
ALGO     = Normal
IALGO    = 48
ISTART   = 0
LPLANE   = .TRUE.
LCHARG   = .FALSE.
LWAVE    = .FALSE.
IWAVPR   = 11
PSTRESS  = 0.001
LVDW = .TRUE. # ADD VDW INTERACTION
IVDW =12
##— Step: NPT-MD ——##
ENCUT    = 550
EDIFF    = 1E-4
ALGO     = Normal
IALGO    = 48
MAXMIX   = 40
IBRION   = 0
NSW      = 1000
NBLOCK   = 1
KBLOCK   = 10
POTIM    = 2
ISYM     = 0
ISPIN=1
# NPT ensemble
ISIF     = 4
MDALGO   = 3
PSTRESS  = 50
TEBEG    = 300
PMASS    = 5000
LANGEVIN_GAMMA = 10 10
LANGEVIN_GAMMA_L = 1 1
LREAL    = False
NELMIN   = 4
PREC     = Normal
ISTART   = 0
ISMEAR   = 2
SIGMA    = 0.2
NPAR     = 4
NCORE    = 1
NSIM     = 4
NWRITE   = 0
LCHARG   = .FALSE.
LPLANE   = .TRUE.
LWAVE    = .FALSE.
IWAVPR   = 11
ISPIN      = 1
LVDW = .TRUE. #ADD VDW INTERACTION
IVDW =12
##—— Step: NVT-MD ——##
ENCUT    = 550
EDIFF    = 1E-4
ALGO     = Normal
IALGO    = 48
MAXMIX   = 40
IBRION   = 0
NSW      = 1000
NBLOCK   = 1
KBLOCK   = 10
POTIM    = 2
ISYM     = 0
ISPIN=1

# NVT ensemble
ISIF     = 2
SMASS    = 2
MDALGO   = 2
TEBEG    = 300
PSTRESS  = 50
LREAL    = False
NELMIN   = 4
PREC     = Normal
ISTART   = 0
ISMEAR   = 2
SIGMA    = 0.2
NPAR     = 4
NCORE    = 1
NSIM     = 4
NWRITE   = 0
LCHARG   = .FALSE.
LPLANE   = .TRUE.
LWAVE    = .FALSE.
IWAVPR   = 11
ISPIN      = 1
LVDW = .TRUE. # ADD VDW INTERACTION
IVDW =12
```

The flowchart of ElasTool package is presented in Fig.S1. As explained above, the main input file for the ElasTool toolkit is supplied through the `elastool.in` file. Crystal structure information is read by ElasTool either in the standard VASP format or in the Crystal Information Format (CIF). The crystal structure is then optimized at fixed pressure or volume using VASP as the calculator[1]. After the structural optimization, based on the strain values supplied by the user in the input file, ElasTool applies different deformation matrices based on the symmetry and further optimizes all the atomic positions based at each specified strain value. If it is a zero-temperature calculations, ElasTool computes all the stress tensors corresponding to each deformation matrix. If it is finite-temperature elastic tensor calculations, ElasTool averages the thermal stresses based on the number of last MD steps specified by the user. To obtain the elastic tensor, ElasTool fits first-order function to the collected stress-strain data. Further data visualization of the mechanical properties can be performed by supplying the computed elastic tensor to codes such as EIAM,[2] ELATE,[3], etc.

In Tables S3 and S4, we present the elastic and mechanical properties for several 2D materials and their heterostructures computed at zero-temperature and a temperature of 300 K, respectively. The database will be

continually updated and hosted on our website in the future.

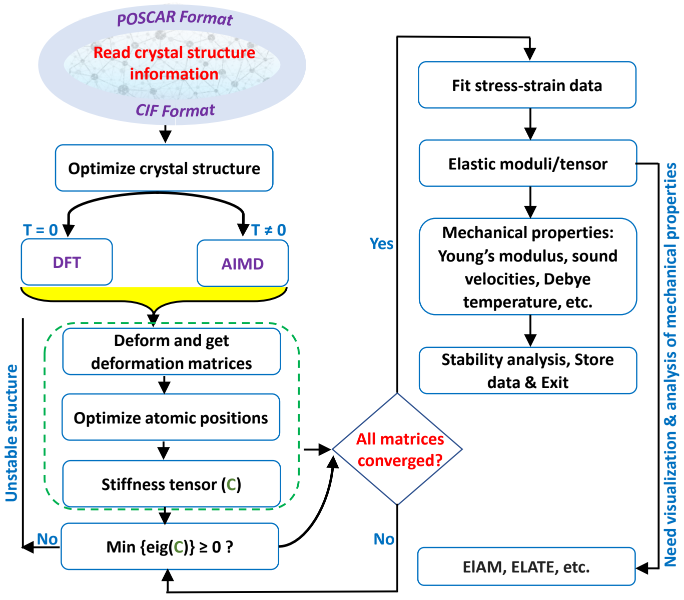

FIG. S1. The flowchart of ElasTool for computing the elastic and mechanical properties of materials. By using external opensource codes such as the EIAM,[2] ELATE,[3] etc., one can further visualize the mechanical properties of materials.

MACHINE LEARNING ALGORITHM

We present the calculated elastic and mechanical properties of several 2D materials and heterostructures at zero-temperature (Table S3) and a temperature of 300 K (Table S4). Data for 600 K are also computed and available in the manuscript GitHub website. Our database currently contains four hundred and eight (408) 2D materials and six thousand six hundred and fifty four (6654) 2D-based heterostructures.

To enable us to gain a deeper understanding of the correlations between the computed mechanical properties and develop a model framework for exploring and exploiting the predicted properties, we employ machine learning (ML) models to predict the lattice constant as the target. Several multilinear regression models were explored, including the boosting models, XGBoost and LightGBM. To make the developed ML model tractable, we focus on those materials with equal lattice constants for a and b. To develop the ML model, we chose the lattice constant (a) as the target. The initial ML-model is developed with $Temp$ (i.e., temperature), SG (i.e., space group), C_{11}, C_{12}, and c (i.e., vacuum size) as the features. Unlike several other calculations where the vacuum size is normally fixed, we self-consistently compute the vacuum size for each material. Then, we employ their pair-plot correlations (see Figures S2) to determine their level of correlation.

Before training and testing our ML model, we set aside 20% each for 2D materials and the heterostructures as unseen data for validation of the developed ML model. In general, an MLR can be written as

$$Y = f(X_1, X_2, ..., X_p) = \beta_o + \sum_{j=1}^{p}\beta_j X_j,$$

where Y is the target, X_j are the features of the model, and the intercept β_o and the coefficients β_j are the model parameters. To begin with, we fit, excluding the data set aside as an unseen sample to the MLR models with default parameters. We initially trained the data using standard multilinear regression (MLR) model to enable us to establish a baseline model; this led to an R^2 score of 0.60 and 0.74 for the 2D materials and 2D-based heterostructures, respectively (see the manuscript code in GitHub for details). To avoid any potential overfitting (or underfitting) we employed a cross-validation (CV) test (also known as out-of-sample testing or rotation estimation); we used the ShuffleSplit CV approach, which is particularly more appropriate for our case because it iteratively and randomly samples data instead of partitioning. In general, the CV will lead to the reduction of the accuracy scores, but will give a better description of the out-of-sample performance.

A more appropriate picture of ML-trained models can be obtained by hyperparameter tuning on top of the cross-validation process. The hyperparameter tuning was carried out using the grid search technique to obtain the optimal hyperparameter combinations for our various ML models based on the CV test. For example, for the XGBoost, we have tuned the number of weak learners, max depth, colsample-bytree, and the learning rate. Finally, the optimal hyperparameters were used to obtain our final ML models. Overall, the boosting ML models showed the best performance with training R^2 close to 1.0 and out-of-sample R^2 and CV accuracy approaching 0.90 for both 2D materials and their heterostructures (see Tables S2). Particularly, the XGBoost had the best combination of the accuracy score and error metrics; it has been used in the description of the results presented in the main text. A Python script providing a detailed step-by-step guide and other information on the implementation of the ML models are provided in a GitHub repository at `https://github.com/gmp007/2D_Elastic-Properties` and the obtained accuracy scores and error metrics are presented in Table S2 for 2D materials and 2D-based heterostructures.

Feature Importance

Presented in Figure S4 is the distribution of the feature importance in our best ML model - XGBoost for both 2D materials and 2D-based heterostructures. In both cases, all the features play a significant role in the model prediction. However, the level of importance is switched for 2D materials and 2D-based heterostructures. In the 2D materials, our ML model identifies C_{11} as the most

TABLE S2. The model parameters for the various machine learning regression models showing the accuracy scores: adjusted R^2 value for the training data (adj-R^2-Train), R^2 score for the test data (R^2-Test), cross-validation score (CV-score) and the error metrics: mean-squared error (MSE), mean-absolute error (MAE), and the mean-absolute-percentage error (MAPE) for 2D-based materials and 2D-based heterostructures.

Model	Adj-R^2-Train	R^2-Test	CV-Score	MSE	MAE	MAPE
	**2D Materials**					
**Linear**	0.63	0.59	0.57	0.15	0.28	7.86
**Bayesian Ridge**	0.60	0.56	0.55	0.17	0.30	8.27
**Lasso Lars**	0.51	0.43	0.46	0.21	0.33	9.10
**Lars**	0.62	0.59	0.57	0.15	0.28	7.86
**XGBoost**	1.0	0.89	0.80	0.04	0.14	4.01
**LightGBM**	0.99	0.85	0.75	0.06	0.15	4.39
	**2D-based Heterostructures**					
**Linear**	0.74	0.74	0.74	0.04	0.14	3.81
**Bayesian Ridge**	0.74	0.74	0.74	0.04	0.14	3.81
**Lasso Lars**	0.0	0.0	0.0	0.16	0.28	8.11
**Lars**	0.74	0.74	0.74	0.04	0.14	3.81
**XGBoost**	0.97	0.88	0.86	0.02	0.08	2.35
**LightGBM**	0.92	0.85	0.85	0.02	0.09	2.53

important feature contributing ~55%. Temperature and the vacuum are the next contributing ~20% and 13%, respectively to the developed model. On the other hand, the vacuum size is the most important feature for the 2D-based heterostructures controlling more than half ~ 58% to the developed model. While the C_{11} is still important here, it contributes ~22%

Out-of-sample Accuracy

The performance of a machine learning model in production is an essential step in developing a robust algorithm that is capable of predicting target to within acceptable accuracy. We have performed a rigorous out-of-sample analysis to determine the accuracy of the developed ML model when applied to unseen data, i.e., the dataset that was not part of both training and testing samples. As explained in the code documentation, before training our model, we set aside ~20% of the data for both 2D materials and 2D-based heterostructures for additional model validation as unseen data (see Figure 3 in the main text and the manuscript code in GitHub). For both the 2D materials and the heterostructures, the performance of our ML model is at the same level as that from our cross-validation analysis. Additionally, for the 2D materials, we have obtained from the current literature about 12 materials that meet the same standard as our data, i.e., reliable vacuum size and isotropic crystal lattice (lattice constants a and b are the same) to further validate the model (see Figure S5 and additional details in the manuscript code in GitHub). Again, the level of accuracy remained very high with an R^2 score that is basically the same as our CV score.

TABLE S3: Calculated lattice constants $a(b)$, mechanical, and elastic properties of 2D materials at zero pressure and temperature. The lattice constants are in Å, the in-plane stiffness K (i.e., the 2D equivalent of bulk modulus), the shear modulus G, the 2D Young's modulus Y^{2D}, and the elastic constant tensor C_{ij} are in N/m; ν is the Poisson ratio; and V_l and V_t are the longitudinal and shear sound velocity in km/s, respectively. The superscript (†) indicated auxetic materials, i.e., negative Poisson ratio, which implies anti-rubber behavior. Note for isotropic 2D materials, $C_{66} = (C_{ii} - C_{ij})/2$, where $i = 1, 2$ and has been omitted in the table. Superscript ♣ denotes an unstable structure.

Material	a(b)	SG	K	G	Y^{2D}	$C_{11}(C_{22})$	C_{12}	C_{66}	ν	V_l	V_t
					**2D Materials**						
**Graphene**	2.47	P6/mmm	216.43	148.47	352.25	364.90	67.97	-	0.19	21.96	14.01
**Borophene**	3.25(4.51)	$P2_1/m$	153.86	139.20	292.31	333.57(252.43)	14.11	101.45	0.05	17.25	11.89
**Germanene**	4.04	P-3m1	31.03	15.79	41.85	46.81	15.24	-	0.33	5.24	3.04
**Silicene**	3.85	P-3m1	44.69	23.54	61.67	68.22	21.15	-	0.31	9.68	5.69
**Stanene**	4.62	P-3m1	19.45	7.87	22.41	27.31	11.58	-	0.42	3.58	1.92
**Phosphorene**	3.23(4.35)	Pmna	14.24/779.78	11.69/60.28	25.68/223.83	244.40(28.04)	24.02	37.40	0.10/.86	2.98/16.93	3.27
**BN**	2.51	P-6m2	178.71	111.85	275.17	290.55	66.86	-	0.23	19.62	12.17
**BP**	3.21	P-6m2	93.81	53.18	135.76	146.99	40.62	-	0.28	13.73	8.26
**BAs**	3.38	P-6m2	82.35	45.04	116.47	127.40	37.31	-	0.29	9.41	5.60

Continued on next page

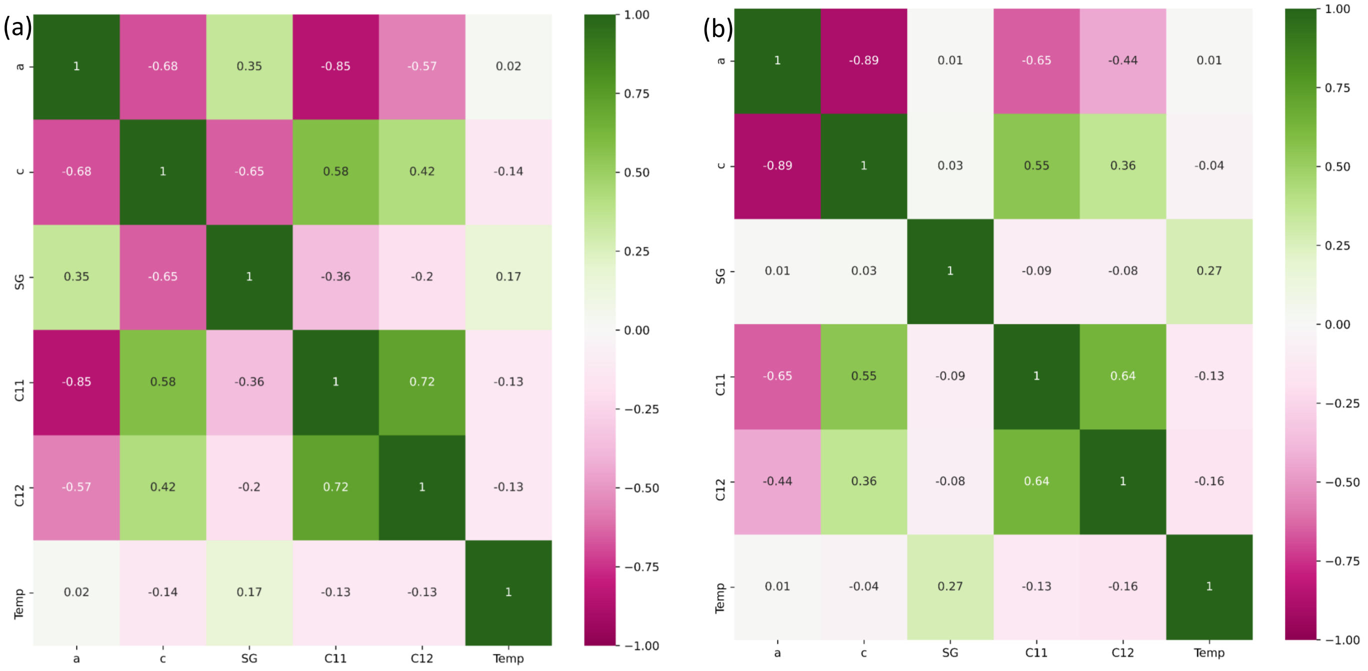

FIG. S2. The Spearman's pair-correlation relations of the model features and the target for (a) 2D materials and (b) 2D-based heterostructures.

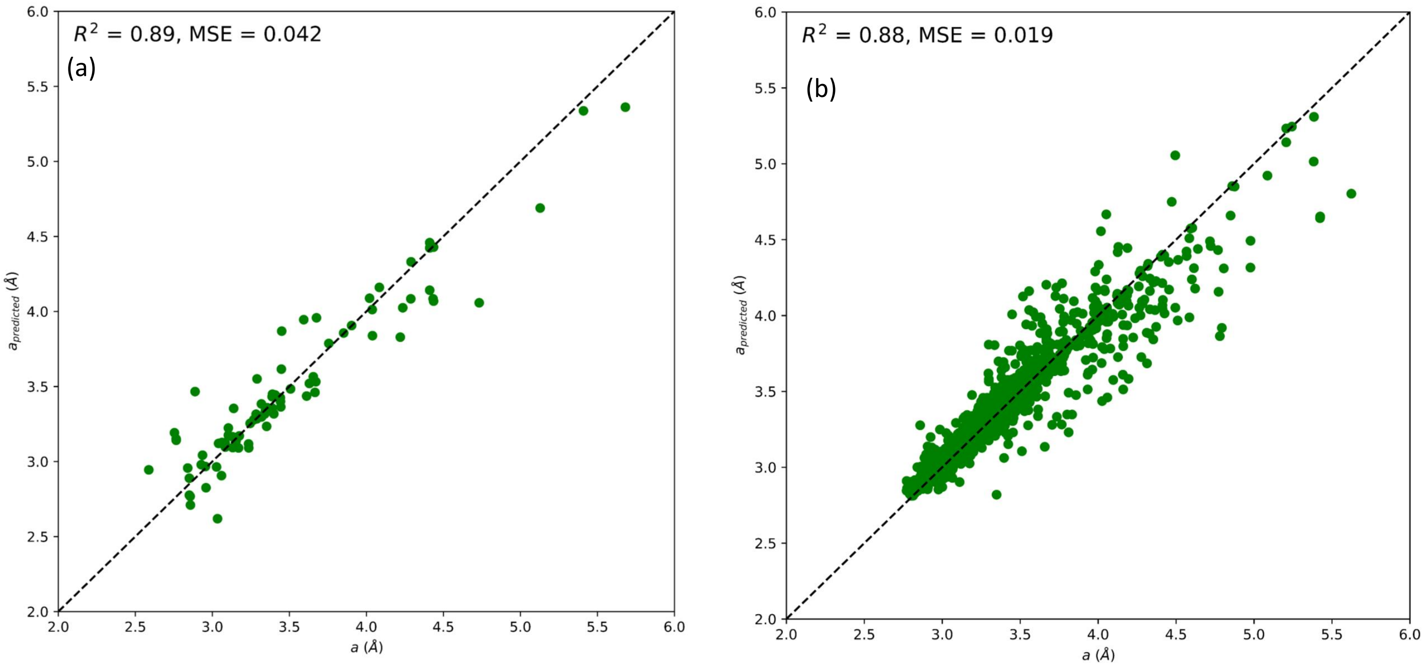

FIG. S3. The relation between the computed lattice constants from our first-principles calculations and the predicted lattice constants from our machine learning model for the test sample data for (a) 2D materials and (b) 2D-based heterostructures. In both 2D and heterostructures, the accuracy (R^2) score is basically the same. Note the R^2 value in the training data is practically unity.

Continued on next page

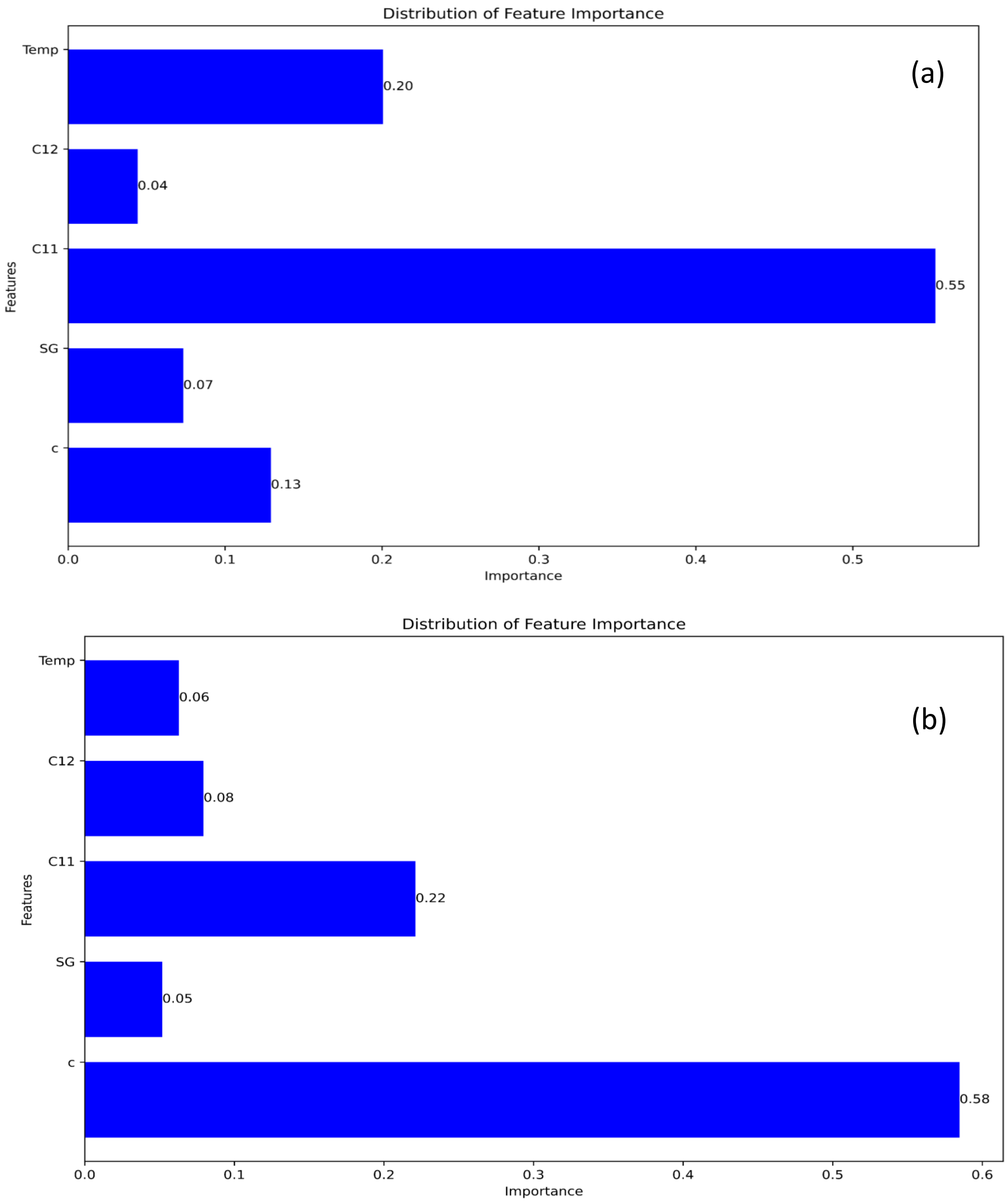

FIG. S4. The fractional representation of the feature importance in (a) 2D materials and (b) 2D-based heterostructures. In 2D materials the most significant feature is the C_{11} and in 2D-based heterostructures, it is the vacuum size.

TABLE S3 – Continued from previous page

Material	a(b)	SG	K	G	Y^{2D}	$C_{11}(C_{22})$	C_{12}	C_{66}	ν	V_l	V_t

TABLE S3 – Continued from previous page

Material	a(b)	SG	K	G	Y^{2D}	$C_{11}(C_{22})$	C_{12}	C_{66}	ν	V_l	V_t
**AlN**	3.12	P-6m2	105.47	37.12	109.84	142.59	68.34	-	0.48	13.30	6.78
**AlP**	3.93	P-6m2	55.89	18.54	55.68	74.43	37.36	-	0.50	10.16	5.07
**AlAs**	4.08	P-6m2	47.63	16.64	49.33	64.27	30.99	-	0.48	7.40	3.76
**GaN**	3.25	P-6m2	103.37	33.92	102.15	137.29	69.45	-	0.51	9.50	4.72
**GaP**	3.93	P-6m2	53.72	20.89	60.17	74.62	32.83	-	0.44	7.73	4.09
**GaAs**	4.10	P-6m2	41.49	17.34	48.92	58.83	24.14	-	0.41	5.96	3.24
**InN**	3.63	P-6m2	76.13	17.05	55.73	93.19	59.08	-	0.63	7.05	3.02
**InP**	4.28	P-6m2	42.09	13.35	40.55	55.45	28.74	-	0.52	6.03	2.96

Continued on next page

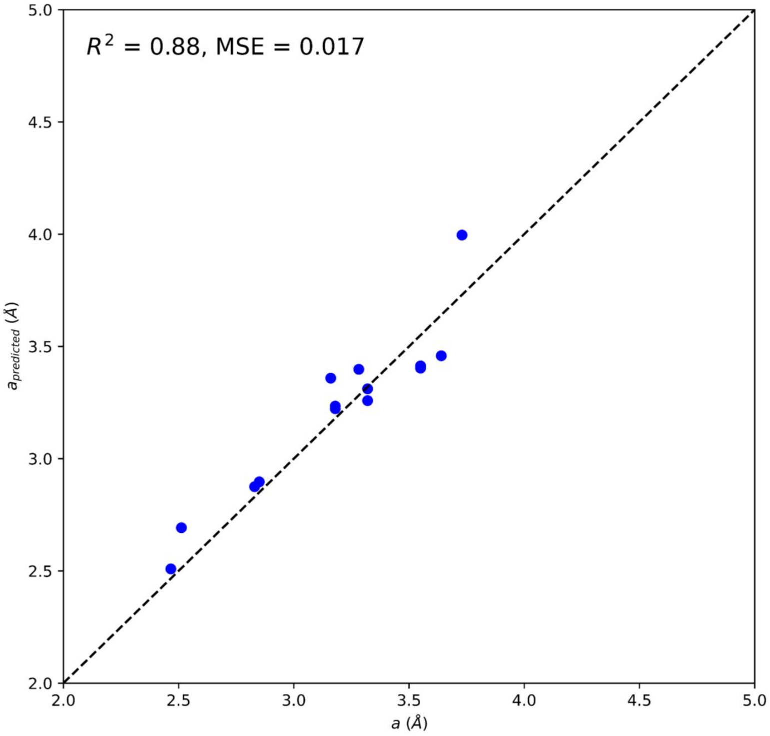

FIG. S5. The relation between some lattice constants from literature (see the code for details) and those predicted with our machine learning model for 2D materials. This further validates the high accuracy of our ML model when applied to unknown (independent) data.

TABLE S3 – Continued from previous page

Material	a(b)	SG	K	G	Y^{2D}	$C_{11}(C_{22})$	C_{12}	C_{66}	ν	V_l	V_t
**InAs**	4.44	P-6m2	34.13	10.85	32.94	44.98	23.28	-	0.52	4.93	2.42
**2H-MoS_2**	3.15	P-6m2	87.53	55.50	135.86	143.03	32.03	-	0.22	6.80	4.24
**2H-$MoSe_2$**	3.28	P-6m2	72.64	47.85	115.38	120.48	24.79	-	0.21	5.16	3.25
**2H-$MoTe_2$**	3.50	P-6m2	56.02	36.85	88.91	92.87	19.18	-	0.21	4.11	2.59
**2H-WS_2**	3.15	P-6m2	94.04	63.41	151.49	157.45	30.64	-	0.19	5.74	2.58
**2H-WSe_2**	3.28	P-6m2	76.60	54.74	127.70	131.34	21.86	-	0.17	4.65	3.00
**2H-WTe_2**	3.51	P-6m2	56.92	42.76	97.67	99.68	14.16	-	0.14	3.82	2.50
**2H-CrS_2**	3.01	P-6m2	80.57	48.57	121.21	129.14	32.00	-	0.25	7.25	4.45
**2H-$CrSe_2$**	3.18	P-6m2	67.88	38.33	97.99	106.21	29.54	-	0.28	5.16	3.10
**2H-$CrTe_2$**	3.49	P-6m2	62.23	25.04	71.43	87.27	37.19	-	0.45	4.25	2.27
**2H-MoO_2**	2.84	P-6m2	153.17	73.91	199.41	227.08	79.26	-	0.35	8.64	4.93
**2H-WO_2**	2.84	P-6m2	172.85	88.69	234.45	261.54	84.17	-	0.32	7.14	4.16
**2H-CrO_2**	2.75	P-6m2	89.30	53.32	133.54	142.62	35.99	-	0.25	8.19	5.01
**2H-HfS_2**	3.51	P-6m2	64.48	29.09	80.18	93.56	35.39	-	0.38	4.97	2.77
**2H-$HfSe_2$**	3.64	P-6m2	54.47	24.76	68.08	79.23	29.71	-	0.38	4.03	2.25
**2H-$HfTe_2$**	3.85	P-6m2	34.35	15.58	42.88	49.93	18.77	-	0.38	2.99	1.61
**2H-TiS_2**	3.31	P-6m2	60.96	30.58	81.46	91.54	30.38	-	0.33	6.82	3.94
**2H-$TiSe_2$**	3.45	P-6m2	47.88	22.13	60.53	70.00	25.75	-	0.37	4.59	2.58
**2H-$TiTe_2$**	3.68	P-6m2	29.46	9.81	29.43	39.27	19.66	-	0.50	3.02	1.51
**2H-ZrS_2**	3.54	P-6m2	59.20	25.62	71.52	84.81	33.58	-	0.40	5.98	3.28
**2H-$ZrSe_2$**	3.66	P-6m2	49.85	21.50	60.09	71.35	28.34	-	0.40	4.48	2.46
**2H-$ZrTe_2$**	3.87	P-6m2	30.80	12.14	34.83	42.93	18.66	-	0.43	3.11	1.66
**2H-TiO_2**	2.88	P-6m2	121.00	45.64	132.56	166.64	75.36	-	0.45	9.51	4.98
**2H-HfO_2**	3.10	P-6m2	124.32	42.90	127.57	167.21	81.42	-	0.49	6.32	3.20
**2H-ZrO_2**	3.14	P-6m2	115.86	36.88	111.89	152.74	78.99	-	0.52	7.98	3.92
**2H-GeO_2**	2.84	P-6m2	119.81	72.31	180.38	192.12	47.50	-	0.25	8.79	5.39
**2H-GeS_2**	3.36	P-6m2	62.42	32.40	85.31	94.82	30.02	-	0.32	6.38	3.73

Continued on next page

TABLE S3 – Continued from previous page

Material	a(b)	SG	K	G	Y^{2D}	$C_{11}(C_{22})$	C_{12}	C_{66}	ν	V_l	V_t
**2H-GeSe$_2$**	3.50	P-6m2	27.11	13.85	36.66	40.96	13.27	-	0.32	3.37	1.96
**2H-GeTe$_2$**	3.57	P-6m2	37.51	0.70	2.76	38.21	36.80	-	0.96	2.78	0.38
**2H-SiO$_2$**	2.72	P-6m2	113.06	75.67	181.31	188.72	37.39	-	0.20	11.02	6.98
**2H-SiS$_2$**	3.21	P-6m2	82.06	55.84	132.92	137.90	26.22	-	0.19	8.98	5.71
**2H-SiSe$_2$**	3.40	P-6m2	52.16	35.35	84.28	87.51	16.81	-	0.19	5.32	3.38
**2H-SiTe$_2$**	3.53	P-6m2	46.23	0.91	3.57	47.14	45.32	-	0.96	3.29	0.46
**2H-SnO$_2$**	3.09	P-6m2	107.80	53.75	143.47	161.55	54.05	-	0.33	7.31	4.21
**2H-SnS$_2$**	3.79	P-6m2	59.92	36.59	90.88	96.51	23.33	-	0.24	5.93	3.65
**2H-SnSe$_2$**	3.75	P-6m2	47.11	24.36	64.22	71.46	22.75	-	0.32	4.35	2.54
**2H-SnTe$_2$**[♣]	-	-	-	-	-	-	-	-	-	-	-
**2H-PbO$_2$**	3.26	P-6m2	78.12	38.07	102.38	116.19	40.06	-	0.34	5.19	2.97
**2H-PbS$_2$**	3.72	P-6m2	44.26	22.55	59.75	66.81	21.71	-	0.33	4.22	2.45
**2H-PbSe$_2$**[♣]	-	-	-	-	-	-	-	-	-	-	-
**2H-PbTe$_2$**[♣]	-	-	-	-	-	-	-	-	-	-	-
**GeS**	3.72(4.23)	Pmn2_1	16.27	4.40	12.34/20.08	53.90(33.11)	33.46	24.68	0.62/1.01	11.90	1.41
**GeSe**	3.96/4.24	Pmn2_1	20.33	6.46	17.48/28.15	59.11(36.70)	33.71	26.15	0.57/0.92	5.345	1.47
**GeTe**	4.22(4.36)	Pmn2_1	39.18	6.54	19.79/24.57	46.60(37.54)	28.76	28.69	0.62/0.77	3.52	1.36
**SiS**	3.35(4.57)	Pmn2_1	13.02/254.99	10.64	18.21/54.11	74.01(24.91)	22.27	17.75	0.30/0.89	9.15	2.82
**SiSe**	3.75(4.29)	Pmn2_1	15.02/81.35	3.02	9.05/14.06	58.42(37.59)	40.84	31.74	0.70/1.09	4.51	1.17
**SiTe**	4.10(4.32)	Pmn2_1	32.13/68.74	4.92	16.46/19.07	53.05(45.81)	39.46	33.91	0.74/0.86	4.29	1.30
**SnS**	4.07(4.15)	Pmn2_1	16.51	4.01	12.90	20.52	12.51	22.97	0.61	2.63	1.16
**SnSe**	4.27(4.28)	Pmn2_1	15.05	4.49	13.84	19.54	10.55	26.33	0.54	2.33	1.12
**SnTe**	4.58(4.72)	Pmn2_1	14.96/75.72	3.73	10.98/15.56	36.02(25.42)	22.81	19.85	0.63/0.90	3.40	0.99
**PbS**	4.07	P4/mmm	35.11	9.08	28.86	44.19	26.03	23.27	0.59	4.29	1.95
**PbS**	4.22	P4/mmm	33.75	7.55	24.69	41.31	26.20	21.67	0.63	3.93	1.68
**PbTe**	4.47	P4/mmm	28.99	5.10	17.34	34.09	23.90	19.36	0.70	3.50	1.35
**1T-MoS$_2$**[†]	3.14	P-3m1	51.75	60.11	111.23	111.85	-8.36	-	-0.07	5.98	4.39
**1T-MoSe$_2$**[†]	3.24	P-3m1	55.53	57.35	112.85	112.88	-1.82	-	-0.02	4.93	3.51
**1T-MoTe$_2$**	3.44	P-3m1	45.58	43.91	89.46	89.49	1.67	-	0.02	3.97	2.78
**1T-WS$_2$**[†]	3.16	P-3m1	51.83	58.99	110.36	110.82	-7.16	-	-0.06	4.82	3.52
**1T-WSe$_2$**[†]	3.25	P-3m1	54.69	58.97	113.51	113.67	-4.28	-	-0.04	4.28	3.08
**1T-WTe$_2$**[†]	3.76	P-3m1	19.06	23.61	42.19	42.67	-4.54	-	-0.11	2.68	1.99
**1T-CrS$_2$**[†]	3.02	P-3m1	54.02	54.63	108.65	108.66	-0.61	-	-0.01	6.67	4.73
**1T-CrSe$_2$**	3.44	P-3m1	21.19	20.86	42.04	42.05	0.33	-	0.01	3.51	2.48
**1T-CrTe$_2$**[†]	3.42	P-3m1	27.63	30.06	57.58	57.69	-2.43	-	-0.04	3.38	2.44
**1T-MoO$_2$**	2.93	P-3m1	83.21	25.61	78.34	108.82	57.60	-	0.53	6.16	2.99
**1T-WO$_2$**	2.92	P-3m1	92.05	12.73	44.73	104.77	79.32	-	0.76	7.14	4.16
**1T-CrO$_2$**[†]	2.87	P-3m1	29.30	34.02	62.96	63.31	-4.72	-	-0.07	5.68	4.17
**1T-HfS$_2$**	3.61	P-3m1	49.92	34.98	82.27	84.90	14.95	-	0.18	4.88	3.13
**1T-HfSe$_2$**	3.73	P-3m1	41.87	28.64	68.02	70.51	13.23	-	0.19	3.90	2.49
**1T-HfTe$_2$**	3.90	P-3m1	27.12	19.91	45.93	47.04	7.21	-	0.15	2.94	1.91
**1T-TiS$_2$**	3.37	P-3m1	46.73	31.62	75.43	78.35	15.11	-	0.19	6.44	4.09
**1T-TiSe$_2$**	3.49	P-3m1	37.61	25.37	60.60	62.98	12.24	-	0.19	4.40	2.79
**1T-TiTe$_2$**	3.68	P-3m1	28.01	10.86	31.31	38.87	17.15	-	0.44	3.01	1.59
**1T-ZrS$_2$**	3.64	P-3m1	47.21	32.31	76.73	79.52	14.90	-	0.19	5.95	3.79
**1T-ZrSe$_2$**	3.76	P-3m1	38.36	25.53	61.31	63.89	12.83	-	0.20	4.35	2.75
**1T-ZrTe$_2$**	3.90	P-3m1	25.48	16.63	40.26	42.12	8.85	-	0.21	3.10	1.95
**1T-TiO$_2$**	3.10	P-3m1	74.48	48.94	118.14	123.42	25.53	-	0.21	8.81	5.55
**1T-HfO$_2$**	3.26	P-3m1	96.91	64.20	154.46	161.11	32.71	-	0.20	6.52	4.12
**1T-ZrO$_2$**	3.29	P-3m1	94.30	59.46	145.87	153.77	34.84	-	0.23	8.39	5.22
**1T-GeO$_2$**	2.94	P-3m1	99.69	58.81	147.95	158.50	40.89	-	0.26	8.25	5.03
**1T-GeS$_2$**	3.42	P-3m1	58.87	35.98	89.33	94.85	22.89	-	0.24	6.50	4.00
**1T-GeSe$_2$**	3.59	P-3m1	48.65	17.69	51.89	66.34	30.96	-	0.47	4.40	2.27
**1T-GeTe$_2$**[♣]	-	-	-	-	-	-	-	-	-	-	-
**1T-SiO$_2$**	2.78	P-3m1	106.00	67.07	164.31	173.07	38.93	-	0.21	10.79	6.71
**1T-SiS$_2$**	3.27	P-3m1	73.62	49.66	118.63	123.28	23.95	-	0.19	8.63	5.48
**1T-SiSe$_2$**	3.47	P-3m1	60.10	36.07	90.17	96.17	24.02	-	0.25	5.70	3.49
**1T-SiTe$_2$**	3.75	P-3m1	49.24	7.80	26.93	57.04	41.44	-	0.73	3.84	1.42
**1T-SnO$_2$**	3.22	P-3m1	85.02	48.20	123.05	133.23	36.82	-	0.28	6.90	4.15
**1T-SnS$_2$**	3.66	P-3m1	46.65	29.09	71.66	75.74	17.56	-	0.23	5.38	3.33

Continued on next page

TABLE S3 – Continued from previous page

Material	a(b)	SG	K	G	Y^{2D}	$C_{11}(C_{22})$	C_{12}	C_{66}	ν	V_l	V_t
**1T-SnSe$_2$**	3.82	P-3m1	39.87	24.63	60.91	64.51	15.24	-	0.24	4.22	2.61
**1T-SnTe$_2$**	4.07	P-3m1	30.77	7.90	25.13	38.66	22.87	-	0.59	2.99	1.35
**1T-PbO$_2$**	3.40	P-3m1	62.27	31.91	84.40	94.19	30.36	-	0.32	4.87	2.83
**1T-PbS$_2$**	3.81	P-3m1	35.33	19.94	50.98	55.27	15.39	-	0.28	3.92	2.36
**1T-PbSe$_2$**	3.94	P-3m1	29.23	11.46	32.93	40.69	17.77	-	0.44	3.01	1.60
**1T-PbTe$_2$**♣	-	-	-	-	-	-	-	-	-	-	-
**BaO**	4.26	P-6m2	43.33	2.24	8.53	45.58	41.09	-	0.90	5.30	1.18
**BaS**	5.11	P-6m2	23.91	1.39	5.27	25.30	22.52	-	0.89	4.51	1.06
**BaSe**	5.33	P-6m2	20.85	1.07	4.08	21.93	19.78	-	0.90	3.87	0.86
**BaTe**	5.69	P-6m2	17.62	0.54	2.09	18.15	17.08	-	0.94	3.40	0.59
**BeO**	2.77	P-6m2	73.99	42.63	108.18	116.62	31.37	-	0.27	13.63	8.24
**BeS**	3.44	P-6m2	55.84	23.29	65.74	79.13	32.55	-	0.41	10.92	5.92
**BeSe**	3.66	P-6m2	48.11	20.19	56.89	68.30	27.91	-	0.41	7.35	4.00
**BeTe**	4.01	P-6m2	39.09	16.30	46.00	55.39	22.80	-	0.41	5.83	3.16
**CdO**	3.67	P-6m2	58.01	4.89	18.04	62.90	53.12	-	0.84	5.87	1.64
**CdS**	4.24	P-6m2	38.65	7.84	26.06	46.48	30.81	-	0.66	5.49	2.24
**CdSe**	4.44	P-6m2	33.60	6.99	23.14	40.59	26.61	-	0.66	4.66	1.94
**CdTe**	4.73	P-6m2	28.71	6.31	20.70	35.02	22.40	-	0.64	4.13	1.75
**MgO**	3.29	P-6m2	64.91	14.55	47.55	79.46	50.36	-	0.63	10.56	4.52
**MgS**	4.08	P-6m2	38.25	7.28	24.45	45.52	30.97	-	0.68	8.37	3.34
**MgSe**	4.29	P-6m2	32.72	6.38	21.35	39.10	26.34	-	0.67	6.02	2.43
**MgTe**	4.66	P-6m2	26.59	5.25	17.55	31.85	21.34	-	0.67	4.87	1.98
**SrO**	4.02	P-6m2	45.24	4.68	16.97	49.92	40.56	-	0.81	6.37	1.95
**SrS**	4.83	P-6m2	26.19	1.75	6.57	27.94	24.44	-	0.87	5.32	1.33
**SrSe**	5.04	P-6m2	22.88	1.41	5.31	24.29	21.47	-	0.88	4.39	1.06
**SrTe**	5.41	P-6m2	19.02	0.81	3.10	19.83	18.21	-	0.92	3.75	0.76
**ZnO**	3.28	P-6m2	73.16	12.66	43.17	85.82	60.50	-	0.70	7.70	2.96
**ZnS**	3.87	P-6m2	47.02	14.24	43.73	61.26	32.77	-	0.53	7.01	3.38
**ZnSe**	4.08	P-6m2	40.11	12.02	36.98	52.13	28.10	-	0.54	5.60	2.69
**ZnTe**	4.41	P-6m2	32.61	10.34	31.40	42.95	22.28	-	0.52	4.75	2.33
**CoSiS$_3$**	5.71	C3i	225.34	126.26	323.68	351.60	99.08	-	0.28	7.38	4.42
**CoSiSe$_3$**	6.07	C3i	173.62	98.24	250.95	271.86	75.38	-	0.28	5.18	3.11
**CoSiTe$_3$**	6.61	C3i	125.18	75.50	188.39	200.68	49.68	-	0.25	4.02	2.47
**CrSiS$_3$**♣	-	-	-	-	-	-	-	-	-	-	-
**CrSiSe$_3$**	6.05	C3i	189.20	10.29	39.05	199.49	178.90	-	0.90	4.48	1.02
**CrSiTe$_3$**	6.62	C3i	102.52	5.09	19.40	107.61	197.43	-	0.91	2.98	0.65
**CuSiS$_3$**	5.86	C3i	151.65	62.93	177.89	214.57	88.72	-	0.41	5.83	3.16
**CuSiSe$_3$**	6.17	C3i	88.60	29.99	89.62	118.59	58.61	-	0.49	3.45	1.74
**CuSiTe$_3$**	6.73	C3i	57.62	30.50	79.78	88.12	27.12	-	0.31	2.70	1.59
**FeSiS$_3$**	5.93	C3i	114.81	80.45	189.21	195.26	34.36	-	0.18	5.75	3.69
**FeSiSe$_3$**	6.14	C3i	100.20	61.01	151.68	161.21	39.19	-	0.24	4.06	2.50
**FeSiTe$_3$**	6.70	C3i	94.54	60.35	147.34	154.89	34.19	-	0.22	3.60	2.25
**MnSiS$_3$**	5.92	C3i	260.57	13.55	51.52	274.12	247.02	-	0.90	6.82	1.52
**MnSiSe$_3$**	6.11	C3i	63.68	40.80	99.47	104.48	22.88	-	0.22	3.26	2.04
**MnSiTe$_3$**	6.68	C3i	120.84	19.51	67.18	140.35	101.34	-	0.72	3.42	1.27
**NiSiS$_3$**	5.77	C3i	188.32	83.90	232.17	272.22	104.42	-	0.38	6.56	3.64
**NiSiSe$_3$**	6.12	C3i	140.66	69.28	185.67	209.94	71.38	-	0.34	4.60	2.64
**NiSiTe$_3$**	6.66	C3i	100.19	53.19	138.98	153.38	46.99	-	0.31	3.55	2.09
**VSiS$_3$**	5.80	C3i	183.91	49.52	156.07	233.44	134.39	-	0.58	6.24	2.88
**VSiSe$_3$**	6.11	C3i	138.01	31.77	103.31	169.78	106.24	-	0.63	4.17	1.80
**VSiTe$_3$**	6.11	C3i	106.15	33.71	102.34	139.86	72.44	-	0.52	3.40	1.67
**CoGeS$_3$**	5.83	C3i	203.94	113.08	290.99	317.02	90.85	-	0.29	6.41	3.83
**CoGeSe$_3$**	6.16	C3i	153.84	86.12	220.84	239.95	67.72	-	0.28	4.64	2.78
**CoGeTe$_3$**	6.70	C3i	99.88	59.86	149.71	159.74	40.02	-	0.25	3.48	2.13
**CrGeS$_3$**	5.98	C3i	8.48	2.80	8.41	11.27	5.68	-	0.50	1.26	0.63
**CrGeSe$_3$**	6.20	C3i	158.68	6.25	24.03	164.92	152.43	-	0.92	3.90	0.76
**CrGeTe$_3$**	6.72	C3i	86.39	32.53	94.52	118.92	53.86	-	0.45	3.03	1.59
**CuGeS$_3$**	5.93	C3i	148.51	56.08	162.83	204.59	92.43	-	0.45	5.18	2.71
**CuGeSe$_3$**	6.24	C3i	78.31	33.04	92.94	111.35	43.27	-	0.41	3.18	1.73
**CuGeTe$_3$**	6.78	C3i	42.39	30.38	70.79	72.77	12.01	-	0.16	2.37	1.53

Continued on next page

TABLE S3 – Continued from previous page

Material	a(b)	SG	K	G	Y^{2D}	$C_{11}(C_{22})$	C_{12}	C_{66}	ν	V_l	V_t
$\mathbf{FeGeS_3}$	6.07	C3i	109.07	76.63	180.03	185.70	32.44	-	0.17	5.15	3.31
$\mathbf{FeGeSe_3}$	6.29	C3i	107.07	58.46	151.26	165.53	48.60	-	0.29	3.94	2.34
$\mathbf{FeGeTe_3}$	6.78	C3i	99.07	51.91	136.25	150.98	47.15	-	0.31	3.43	2.01
$\mathbf{MnGeS_3}$	6.07	C3i	155.51	32.37	107.17	187.88	123.14	-	0.66	5.18	2.15
$\mathbf{MnGeSe_3}^\dagger$	6.45	C3i	43.52	54.67	96.92	98.19	-11.15	-	-0.11	3.12	2.33
$\mathbf{MnGeTe_3}$	6.82	C3i	29.58	28.03	57.56	57.60	1.55	-	0.03	2.13	1.49
$\mathbf{NiGeS_3}$	5.90	C3i	178.23	73.12	207.40	251.35	105.11	-	0.42	5.78	3.12
$\mathbf{NiGeSe_3}$	6.23	C3i	127.19	52.87	149.39	180.06	74.32	-	0.41	4.06	2.20
$\mathbf{NiGeTe_3}$	6.80	C3i	89.60	35.99	102.71	125.59	53.61	-	0.43	3.13	1.68
$\mathbf{VGeS_3}$	5.93	C3i	160.82	50.32	153.31	211.14	110.50	-	0.52	5.42	2.64
$\mathbf{VGeSe_3}$	6.26	C3i	108.98	18.63	63.65	127.61	90.34	-	0.71	3.47	1.33
$\mathbf{VGeTe_3}$	6.72	C3i	72.15	42.69	107.27	114.83	29.46	-	0.26	2.98	1.82
**2D Heterostructures**											
**Heterostructure with two 1T monolayer**											
$\mathbf{CrO_2GeS_2}$	3.16	P-3m1	148.87	64.33	179.68	213.20	84.54	-	0.40	7.10	3.90
$\mathbf{CrO_2GeSe_2}$	3.22	P-3m1	61.26	22.58	66.00	83.84	38.68	-	0.46	3.79	1.97
$\mathbf{CrO_2GeTe_2}$	3.07	P-3m1	148.87	64.33	179.68	213.20	84.54	-	0.40	7.10	3.90
$\mathbf{CrS_2GeO_2}$	2.96	P-3m1	152.65	115.83	263.44	268.49	36.82	-	0.14	7.46	4.90
$\mathbf{CrS_2GeSe_2}$	3.36	P-3m1	82.27	39.92	107.51	122.19	42.36	-	0.35	4.57	2.61
$\mathbf{CrSe_2GeO_2}$	3.02	P-3m1	142.62	93.00	225.17	235.61	49.62	-	0.21	5.97	3.75
$\mathbf{CrSe_2GeS_2}$	3.33	P-3m1	82.27	71.89	68.37	140.26	3.52	-	0.03	4.84	3.38
$\mathbf{CrSe_2GeTe_2}$	3.26	P-3m1	82.82	26.45	80.20	109.27	56.37	-	0.52	3.36	1.65
$\mathbf{CrTe_2GeO_2}$	3.14	P-3m1	142.78	63.77	176.32	206.55	79.01	-	0.38	5.08	2.82
$\mathbf{CrTe_2GeS_2}$	3.47	P-3m1	83.11	49.83	124.61	132.94	33.28	-	0.25	4.34	2.66
$\mathbf{CrTe_2GeSe_2}$	3.55	P-3m1	80.50	36.63	100.69	117.13	43.87	-	0.37	3.78	2.11
$\mathbf{CrO_2HfS_2}$	3.24	P-3m1	105.88	51.63	138.84	157.52	54.25	-	0.34	5.14	2.94
$\mathbf{CrO_2HfTe_2}$	3.15	P-3m1	133.86	29.14	95.73	163.00	104.72	-	0.64	4.04	1.71
$\mathbf{CrS_2HfO_2}$	3.17	P-3m1	155.67	104.97	250.78	260.64	50.69	-	0.19	6.46	4.10
$\mathbf{CrS_2HfSe_2}$	3.41	P-3m1	62.81	46.19	106.47	109.00	16.62	-	0.15	3.82	2.49
$\mathbf{CrS_2HfTe_2}$	3.51	P-3m1	56.64	14.38	45.87	71.02	42.26	-	0.60	2.88	1.30
$\mathbf{CrSe_2HfO_2}$	3.21	P-3m1	151.53	106.15	249.69	257.69	45.38	-	0.18	5.74	3.69
$\mathbf{CrSe_2HfS_2}$	3.44	P-3m1	88.43	59.69	142.52	148.11	28.75	-	0.19	4.49	2.85
$\mathbf{CrSe_2HeTe_2}$	3.54	P-3m1	69.11	25.36	74.21	94.47	43.75	-	0.46	3.10	1.61
$\mathbf{CrTe_2HfO_2}$	3.29	P-3m1	148.93	96.50	234.23	245.43	52.44	-	0.21	5.18	3.25
$\mathbf{CrTe_2HeS_2}$	3.52	P-3m1	102.71	58.38	148.88	161.08	44.33	-	0.28	4.35	2.62
$\mathbf{CrTe_2HeSe_2}$	3.59	P-3m1	85.08	49.56	125.27	134.64	35.52	-	0.26	3.76	2.28
$\mathbf{CrO_2MnS_2}$	2.88	P-3m1	80.62	70.35	150.26	150.96	10.27	-	0.07	5.67	3.87
$\mathbf{CrO_2MnSe_2}$	2.97	P-3m1	99.65	60.21	150.13	159.86	39.44	-	0.25	4.98	3.06
$\mathbf{CrO_2MnTe_2}$	3.10	P-3m1	145.90	32.61	106.61	178.51	113.29	-	0.63	4.76	2.03
$\mathbf{CrS_2MnO_2}$	3.15	P-3m1	90.58	35.09	101.17	125.67	55.49	-	0.44	5.66	2.99
$\mathbf{CrS_2MnSe_2}^\dagger$	3.11	P-3m1	62.90	63.98	126.87	126.88	-1.09	-	-0.01	4.41	3.13
$\mathbf{CrS_2MnTe_2}$	3.25	P-3m1	65.62	48.12	111.04	113.74	17.51	-	0.15	3.83	2.49
$\mathbf{CrSe_2MnO_2}$	3.00	P-3m1	124.63	63.64	168.52	188.27	60.98	-	0.32	5.46	3.17
$\mathbf{CrSe_2MnS_2}$	3.15	P-3m1	84.50	53.77	131.45	138.28	30.73	-	0.22	4.67	2.91
$\mathbf{CrSe_2MnTe_2}$	3.33	P-3m1	54.71	46.66	100.73	101.37	8.05	-	0.08	3.36	2.28
$\mathbf{CrTe_2MnO_2}$	3.12	P-3m1	146.31	35.20	113.48	181.51	111.11	-	0.61	4.83	2.13
$\mathbf{CrTe_2MnS_2}$	3.31	P-3m1	78.77	37.09	100.86	115.86	41.68	-	0.36	3.94	2.23
$\mathbf{CrTe_2MnSe_2}$	3.38	P-3m1	55.01	42.84	96.33	97.85	12.18	-	0.12	3.35	2.22
$\mathbf{CrO_2MoS_2}$	3.00	P-3m1	141.61	100.43	235.04	242.05	41.18	-	0.17	6.83	4.40
$\mathbf{CrO_2MoSe_2}$	3.07	P-3m1	144.98	65.72	180.89	210.70	79.26	-	0.38	5.53	3.09
$\mathbf{CrO_2MoTe_2}$	3.16	P-3m1	150.37	65.82	183.13	216.19	84.54	-	0.39	5.08	2.80
$\mathbf{CrS_2MoO_2}$	2.95	P-3m1	140.78	92.40	223.14	233.18	48.38	-	0.21	6.59	4.15
$\mathbf{CrS_2MoSe_2}$	3.16	P-3m1	95.99	90.98	186.83	186.97	5.01	-	0.03	5.13	3.58
$\mathbf{CrS_2MoTe_2}$	3.30	P-3m1	93.71	63.97	152.07	157.68	29.74	-	0.19	4.38	2.79
$\mathbf{CrSe_2MoO_2}$	3.02	P-3m1	124.72	76.27	189.30	200.98	48.45	-	0.24	5.32	3.28
$\mathbf{CrSe_2MoS_2}^\dagger$	3.17	P-3m1	95.08	95.48	190.56	190.56	-0.40	-	-0.00	5.20	3.68
$\mathbf{CrSe_2MoTe_2}^\dagger$	3.50	P-3m1	52.23	74.15	122.57	126.38	-21.93	-	-0.17	3.63	2.78
$\mathbf{CrTe_2MoO_2}$	3.15	P-3m1	79.75	54.09	128.92	133.84	25.66	-	0.19	3.99	2.54
$\mathbf{CrTe_2MoS_2}$	3.31	P-3m1	88.72	73.34	160.60	162.06	15.37	-	0.09	4.45	2.99
$\mathbf{CrTe_2MoSe_2}^\dagger$	3.35	P-3m1	51.33	77.79	123.70	129.12	-26.46	-	-0.20	3.67	2.85

Continued on next page

TABLE S3 – Continued from previous page

Material	a(b)	SG	K	G	Y^{2D}	$C_{11}(C_{22})$	C_{12}	C_{66}	ν	V_l	V_t
**CrO_2PbSe_2**	3.02	P-3m1	146.06	6.68	25.57	152.75	139.38	-	0.91	4.02	0.84
**CrS_2PbO_2**	3.26	P-3m1	109.80	64.83	163.05	174.63	44.97	-	0.26	5.22	3.18
**CrS_2PbSe_2**	3.09	P-3m1	98.31	27.11	84.99	125.42	71.21	-	0.57	3.60	1.67
**CrS_2PbTe_2**	3.23	P-3m1	120.18	2.47	9.67	122.65	117.72	-	0.96	3.40	0.48
**$CrSe_2PbO_2$**	3.32	P-3m1	106.05	63.91	159.51	169.96	42.14	-	0.25	4.66	2.86
**$CrSe_2PbS_2$**	3.55	P-3m1	88.93	37.66	105.83	126.59	51.26	-	0.40	4.16	2.27
**$CrSe_2PbTe_2$**	3.28	P-3m1	117.75	13.20	47.46	130.94	104.55	-	0.80	3.31	1.05
**$CrTe_2PbO_2$**	3.42	P-3m1	84.91	58.68	138.80	143.59	26.23	-	0.18	4.00	2.56
**$CrTe_2PbS_2$**	3.67	P-3m1	62.24	35.95	91.15	98.19	26.30	-	0.27	3.45	2.09
**$CrTe_2PbSe_2$**	3.77	P-3m1	37.02	12.29	36.90	49.31	24.73	-	0.50	2.33	1.16
**CrO_2SiS_2**	3.09	P-3m1	167.02	67.17	191.61	234.19	99.85	-	0.43	8.14	8.14
**CrO_2SiSe_2**	3.20	P-3m1	154.58	57.01	166.59	211.58	97.57	-	0.46	6.46	3.36
**CrS_2SiO_2**	2.84	P-3m1	171.60	120.58	283.27	292.18	51.02	-	0.17	8.36	5.37
**CrS_2SiSe_2**	3.32	P-3m1	108.03	57.35	149.86	165.39	50.68	-	0.31	5.60	3.30
**CrS_2SiTe_2**	3.50	P-3m1	61.59	16.92	53.09	78.51	44.67	-	0.57	3.54	1.64
**$CrSe_2SiO_2$**	2.91	P-3m1	166.07	85.85	226.38	251.93	80.22	-	0.32	6.41	3.74
**$CrSe_2SiS_2$**	3.24	P-3m1	120.18	84.93	199.05	205.11	35.25	-	0.17	6.09	3.92
**$CrSe_2SiTe_2$**	3.52	P-3m1	71.52	21.93	67.14	93.45	49.58	-	0.53	3.50	1.69
**$CrTe_2SiO_2$**	3.02	P-3m1	167.86	52.92	160.94	220.78	114.94	-	0.52	5.35	2.62
**$CrTe_2SiS_2$**	3.34	P-3m1	75.77	70.24	145.80	146.01	5.53	-	0.04	4.61	3.20
**$CrTe_2SiSe_2$**	3.49	P-3m1	93.20	55.78	139.58	148.98	37.43	-	0.25	4.38	2.68
**CrS_2SnO_2**	3.15	P-3m1	136.34	88.92	215.27	225.26	47.42	-	0.21	6.62	4.16
**CrS_2SnSe_2**	3.52	P-3m1	88.87	34.82	100.07	123.69	54.05	-	0.44	4.51	2.39
**$CrSe_2SnO_2$**	3.21	P-3m1	129.95	87.37	208.97	217.32	42.58	-	0.20	5.68	3.60
**$CrSe_2SnS_2$**	3.50	P-3m1	96.55	52.20	135.53	148.75	44.34	-	0.30	4.91	2.91
**$CrTe_2SnO_2$**	3.30	P-3m1	120.38	73.65	182.78	194.03	46.73	-	0.24	4.91	3.02
**$CrTe_2SnS_2$**	3.57	P-3m1	104.67	50.88	136.94	155.54	53.79	-	0.35	4.60	2.63
**$CrTe_2SnSe_2$**	3.68	P-3m1	69.71	42.47	105.57	112.18	27.24	-	0.24	3.68	2.27
**CrO_2TiS_2**	3.09	P-3m1	124.04	26.72	87.95	150.77	97.32	-	0.65	6.18	2.60
**GeO_2CrS_2**	2.96	P-3m1	151.62	115.55	262.30	267.17	36.07	-	0.14	7.45	4.90
**GeO_2CrSe_2**	3.02	P-3m1	142.27	93.13	225.14	235.40	49.14	-	0.21	5.97	3.75
**GeO_2CrTe_2**	3.14	P-3m1	142.79	63.78	176.34	206.56	79.01	-	0.38	5.08	2.82
**GeO_2HfS_2**	3.25	P-3m1	98.45	45.22	123.94	143.67	53.24	-	0.37	4.77	2.68
**GeS_2HfO_2**	3.30	P-3m1	167.91	102.39	254.41	270.30	65.52	-	0.24	6.65	4.10
**GeO_2MnS_2**	2.96	P-3m1	119.12	87.05	201.18	206.17	32.07	-	0.16	6.49	4.22
**GeO_2MoS_2**	3.01	P-3m1	163.73	125.78	284.54	289.51	37.94	-	0.13	7.19	4.74
**GeO_2MoSe_2**	3.07	P-3m1	148.95	98.90	237.75	247.85	50.04	-	0.20	5.83	3.68
**GeS_2MoO_2**	3.30	P-3m1	105.43	50.58	136.73	156.01	54.85	-	0.35	5.78	3.29
**GeO_2PbS_2**	3.33	P-3m1	73.40	3.80	14.45	77.21	69.60	-	0.90	3.45	0.76
**$GeTe_2PbSe_2$**	4.47	P-3m1	27.10	10.51	30.29	37.61	16.58	-	0.44	2.38	1.26
**GeO_2SiS_2**	3.10	P-3m1	172.45	97.06	248.41	269.50	75.39	-	0.28	8.27	4.96
**$GeTe_2SiSe_2$**	3.63	P-3m1	59.01	17.26	53.41	76.27	41.75	-	0.55	3.19	1.52
**GeS_2SnO_2**	3.31	P-3m1	141.17	79.71	203.78	220.88	61.46	-	0.28	6.63	3.98
**$GeTe_2SnSe_2$**	3.85	P-3m1	62.41	23.74	68.79	86.15	38.68	-	0.45	3.32	1.74
**$GeTe_2WSe_2$**	3.29	P-3m1	78.84	50.62	123.32	129.47	28.22	-	0.22	3.30	2.07
**HfO_2MoSe_2**	3.23	P-3m1	157.18	124.11	277.39	281.28	33.07	-	0.12	5.74	3.82
**HfS_2MoO_2**	3.37	P-3m1	125.85	48.32	139.67	174.17	77.52	-	0.45	5.28	2.78
**$HfSe_2MoS_2$**	3.46	P-3m1	75.61	59.54	133.24	135.15	16.07	-	0.12	4.12	2.74
**HfS_2PbSe_2**	3.76	P-3m1	71.30	40.16	102.77	111.47	31.14	-	0.28	3.67	2.21
**HfO_2SiS_2**	3.24	P-3m1	181.31	117.62	285.36	298.93	63.69	-	0.21	7.36	4.62
**HfS_2SnO_2**	3.39	P-3m1	122.63	69.73	177.82	192.37	52.90	-	0.27	5.42	3.26
**$HfTe_2TiO_2$**	3.17	P-3m1	118.04	47.96	136.42	166.00	70.08	-	0.42	4.11	2.21
**HfS_2WTe_2**	3.54	P-3m1	89.96	76.74	165.65	166.70	13.22	-	0.08	4.00	2.71
**MnO_2CrS_2**	3.15	P-3m1	91.04	35.67	102.51	126.71	55.38	-	0.44	5.68	3.01
**MnO_2GeS_2**	3.16	P-3m1	151.10	36.27	117.00	187.37	114.28	-	0.61	6.60	2.91
**MnS_2HfO_2**	3.20	P-3m1	150.14	78.82	206.74	228.96	71.33	-	0.31	6.08	3.57
**MnO_2MoS_2**	3.00	P-3m1	139.44	94.32	225.05	233.76	45.11	-	0.19	6.66	4.23
**MnS_2MoO_2**	2.95	P-3m1	105.09	64.34	159.63	169.43	40.75	-	0.24	5.59	3.44
**MnO_2PbS_2**	3.34	P-3m1	77.11	22.36	69.33	99.47	54.75	-	0.55	4.02	1.91
**$MnTe_2PbS_2$**	3.75	P-3m1	36.00	25.93	60.29	61.93	10.07	-	0.16	2.80	1.81

Continued on next page

TABLE S3 – Continued from previous page

Material	a(b)	SG	K	G	Y^{2D}	$C_{11}(C_{22})$	C_{12}	C_{66}	ν	V_l	V_t
**$MnTe_2SnO_2$**	3.29	P-3m1	102.19	61.65	153.82	163.84	40.54	-	0.25	4.48	2.75
**MoS_2GeO_2**	3.01	P-3m1	164.98	126.23	286.05	291.21	38.76	-	0.13	7.21	4.75
**$MoSe_2GeO_2$**	3.07	P-3m1	148.38	98.70	237.09	247.08	49.68	-	0.20	5.82	3.68
**MoS_2HfSe_2**	3.46	P-3m1	75.65	59.71	133.48	135.36	15.94	-	0.12	4.13	2.74
**$MoTe_2HfSe_2$**	2.61	P-3m1	74.51	60.49	133.54	135.00	14.01	-	0.10	3.65	2.44
**MoO_2PbSe_2**	3.03	P-3m1	136.96	9.71	36.25	146.66	127.25	-	0.87	3.77	0.97
**MoS_2SiO_2**	2.90	P-3m1	187.40	115.59	285.98	303.00	71.81	-	0.24	7.78	4.80
**$MoSe_2SiTe_2$**	3.56	P-3m1	74.51	34.06	93.49	108.56	40.45	-	0.37	3.65	2.05
**$MoTe_2WSe_2$**†	3.38	P-3m1	83.56	85.25	168.79	168.80	-1.69	-	-0.01	3.81	2.71
**PbO_2GeTe_2**	3.50	P-3m1	82.94	24.26	75.09	107.21	58.68	-	0.55	3.48	1.65
**PbO_2HfSe_2**	3.54	P-3m1	96.64	54.19	138.88	150.83	42.46	-	0.28	4.13	2.48
**PbO_2MnS_2**	3.29	P-3m1	107.53	46.62	130.08	154.15	60.91	-	0.40	4.93	2.71
**PbO_2MoS_2**	3.30	P-3m1	116.45	76.76	185.05	193.21	39.69	-	0.21	5.25	3.31
**$PbTe_2WSe_2$**	3.31	P-3m1	107.93	28.27	89.62	136.20	79.66	-	0.58	3.11	1.42
**$SiSe_2HfO_2$**	3.33	P-3m1	169.70	97.00	247.01	266.78	72.61	-	0.27	6.24	3.77
**SiO_2MnS_2**	2.82	P-3m1	150.88	106.69	249.98	257.57	44.19	-	0.17	7.73	4.98
**SiO_2MoS_2**	2.91	P-3m1	185.86	115.15	284.40	301.01	70.72	-	0.23	7.76	4.80
**SiO_2PbS_2**	2.88	P-3m1	200.40	14.99	55.78	215.39	185.41	-	0.86	5.30	1.40
**$SiTe_2SnSe_2$**	3.80	P-3m1	80.58	36.95	101.32	117.52	43.63	-	0.37	3.97	2.23
**SiO_2WS_2**	2.92	P-3m1	194.28	116.53	291.36	310.81	77.75	-	0.25	6.69	4.10
**SnO_2GeS_2**	3.31	P-3m1	141.29	79.79	203.97	221.08	61.51	-	0.28	6.63	3.98
**$SnSe_2GeTe_2$**	3.86	P-3m1	61.76	23.69	68.49	85.45	38.07	-	0.45	3.31	1.74
**$SnTe_2GeSe_2$**†	4.05	P-3m1	2.51	17.40	8.76	19.90	-14.89	-	-0.75	1.68	1.57
**SnO_2HfS_2**	3.39	P-3m1	122.47	69.71	177.70	192.18	52.76	-	0.27	5.42	3.26
**$SnTe_2HfSe_2$**	3.91	P-3m1	54.72	27.53	73.26	82.25	27.19	-	0.33	3.04	1.76
**SnO_2MnS_2**	3.18	P-3m1	129.20	61.52	166.70	190.72	67.68	-	0.35	6.11	3.47
**SnO_2PbS_2**	3.47	P-3m1	108.26	47.81	132.66	156.07	60.45	-	0.39	4.81	2.66
**$SnTe_2PbSe_2$**	5.62	P-3m1	28.77	4.61	15.90	33.39	24.16	-	0.72	2.73	1.01
**$SnTe_2SiS_2$**	3.29	P-3m1	139.74	12.31	45.26	152.05	127.43	-	0.84	4.29	1.22
**SnO_2TiTe_2**	3.36	P-3m1	112.29	34.24	104.95	146.53	78.05	-	0.53	4.36	2.11
**$SnTe_2WSe_2$**	3.28	P-3m1	123.81	24.73	82.46	148.54	99.08	-	0.67	3.41	1.39
**TiO_2GeS_2**	3.25	P-3m1	161.06	73.66	202.17	234.71	87.40	-	0.37	7.58	4.25
**$TiTe_2GeSe_2$**	3.65	P-3m1	69.00	30.01	83.66	99.01	38.98	-	0.39	3.59	1.98
**TiO_2HfS_2**	3.27	P-3m1	110.65	67.81	168.18	178.47	42.84	-	0.24	5.55	3.42
**TiO_2MnS_2**	3.01	P-3m1	123.62	83.66	199.58	207.28	39.95	-	0.19	7.01	4.45
**$TiTe_2MnSe_2$**	3.49	P-3m1	69.94	13.85	46.23	83.79	56.09	-	0.67	3.21	1.30
**TiO_2MoS_2**	3.04	P-3m1	161.13	128.97	286.53	290.09	32.16	-	0.11	7.64	5.09
**TiO_2MoSe_2**	3.10	P-3m1	143.37	111.13	250.42	254.50	32.24	-	0.13	6.18	4.08
**TiO_2PbSe_2**	3.06	P-3m1	152.35	37.69	120.86	190.03	114.65	-	0.60	4.56	2.03
**$TiTe_2PbSe_2$**	3.92	P-3m1	34.32	24.48	57.15	58.80	9.85	-	0.17	2.66	1.71
**$TiSe_2SiO_2$**	2.95	P-3m1	110.11	13.90	49.37	124.01	96.21	-	0.78	4.60	1.54
**TiO_2SnS_2**	3.30	P-3m1	140.69	64.77	177.40	205.45	75.92	-	0.37	6.67	3.75
**$TiTe_2SnSe_2$**	3.78	P-3m1	62.41	37.66	93.94	100.07	24.76	-	0.25	3.59	2.20
**TiO_2WS_2**	3.05	P-3m1	160.75	133.03	291.16	293.78	27.72	-	0.09	6.59	4.43
**WSe_2GeO_2**	3.07	P-3m1	150.79	98.29	238.02	249.08	52.50	-	0.21	5.24	3.29
**WO_2HfS_2**	3.39	P-3m1	107.71	40.28	117.27	147.99	67.43	-	0.46	4.39	2.29
**WTe_2HfSe_2**	3.62	P-3m1	74.65	63.45	137.19	138.10	11.20	-	0.08	3.48	2.36
**WO_2MnS_2**	2.95	P-3m1	111.87	47.56	133.49	159.43	64.31	-	0.40	4.64	2.54
**WO_2MoS_2**	3.00	P-3m1	159.49	94.66	237.61	254.15	64.83	-	0.26	5.64	3.44
**WSe_2MoS_2**†	3.21	P-3m1	106.47	111.28	217.64	217.75	-4.81	-	-0.02	4.83	3.45
**WS_2PbO_2**	3.31	P-3m1	120.00	78.80	190.27	198.80	41.20	-	0.21	4.82	3.04
**WTe_2PbSe_2**	3.80	P-3m1	48.92	28.00	71.23	76.92	20.92	-	0.27	2.68	1.62
**WO_2SiS_2**	3.09	P-3m1	148.22	82.88	212.62	231.09	65.34	-	0.28	6.12	3.67
**WS_2SiTe_2**	3.49	P-3m1	78.87	34.66	96.31	113.53	44.21	-	0.39	3.68	2.04
**WTe_2SiS_2**	3.36	P-3m1	111.81	84.60	192.65	196.42	27.21	-	0.14	4.67	3.06
**WSe_2SnS_2**	3.51	P-3m1	92.82	63.32	150.56	156.13	29.50	-	0.19	4.37	2.78
**WTe_2SnSe_2**	3.78	P-3m1	61.03	43.25	101.24	104.28	17.78	-	0.17	3.30	2.12
**WO_2TiS_2**	3.09	P-3m1	107.10	37.82	111.80	144.92	69.28	-	0.48	4.69	2.40
**WS_2TiSe_2**	3.31	P-3m1	89.73	58.74	142.00	148.47	31.00	-	0.21	4.32	2.72
**WSe_2TiTe_2**	3.48	P-3m1	68.38	38.50	98.53	106.88	29.88	-	0.28	3.23	1.94

Continued on next page

TABLE S3 – Continued from previous page

Material	a(b)	SG	K	G	Y^{2D}	$C_{11}(C_{22})$	C_{12}	C_{66}	ν	V_l	V_t
$\mathbf{WTe_2TiSe_2}$	3.48	P-3m1	76.11	67.65	143.27	143.76	8.46	-	0.06	3.76	2.58
			**Heterostructure with two 2H monolayer**								
$\mathbf{CrO_2GeTe_2}$	2.97	P-3m1	156.87	23.01	80.27	179.88	133.86	-	0.74	4.47	1.60
$\mathbf{CrS_2GeO_2}$	2.91	P-3m1	189.97	74.97	215.02	264.94	115.00	-	0.43	7.28	3.87
$\mathbf{CrS_2GeSe_2}$	3.17	P-3m1	128.85	27.48	90.59	156.33	101.38	-	0.65	4.86	2.04
$\mathbf{CrSe_2GeO_2}$	2.98	P-3m1	185.69	102.17	263.63	287.86	83.51	-	0.29	6.52	3.88
$\mathbf{CrSe_2GeS_2}$	3.27	P-3m1	131.63	70.59	183.79	202.22	61.04	-	0.30	5.71	3.37
$\mathbf{CrSe_2GeTe_2}$	3.25	Cm	97.06	3.63	14.01	100.69	93.42	-	0.93	3.21	0.61
$\mathbf{CrTe_2GeO_2}$	3.11	P-3m1	164.66	67.79	192.09	232.46	96.87	-	0.42	5.33	2.88
$\mathbf{CrTe_2GeS_2}$	3.57	P-3m1	52.85	37.01	87.07	89.86	15.84	-	0.18	3.66	2.35
$\mathbf{CrTe_2GeSe_2}$	3.63	Cm	45.16	29.26	71.02	74.42	15.89	-	0.21	3.09	1.94
$\mathbf{CrO_2HfSe_2}$	2.91	P-3m1	167.39	40.02	129.20	207.41	127.37	-	0.61	4.66	1.96
$\mathbf{CrO_2HfTe_2}$	3.02	P-3m1	87.25	37.12	104.17	124.37	50.12	-	0.40	3.39	1.85
$\mathbf{CrS_2HfO_2}$	3.07	P-3m1	206.82	89.28	249.45	296.10	117.54	-	0.40	6.67	3.66
$\mathbf{CrS_2HfSe_2}$	3.35	P-3m1	68.73	18.36	57.95	87.09	50.37	-	0.58	3.35	1.54
$\mathbf{CrS_2HfTe_2}$	3.22	Cm	118.00	43.12	126.33	161.13	74.88	-	0.46	3.98	2.06
$\mathbf{CrSe_2HfO_2}$	3.12	P-3m1	193.82	83.01	232.48	276.83	110.80	-	0.40	5.78	3.26
$\mathbf{CrSe_2HfS_2}$	3.35	P-3m1	104.05	57.90	148.80	161.95	46.14	-	0.28	4.58	2.74
$\mathbf{CrTe_2HfO_2}$	3.21	P-3m1	190.87	101.84	265.62	292.70	89.03	-	0.30	5.51	3.25
$\mathbf{CrTe_2HfS_2}$	3.49	P-3m1	113.46	50.29	139.38	163.75	63.17	-	0.39	4.35	2.41
$\mathbf{CrTe_2HfSe_2}$	3.56	Cm	134.84	46.84	139.07	181.68	87.99	-	0.48	4.31	2.19
$\mathbf{CrO_2MnS_2}$	2.79	P-3m1	137.85	91.12	219.43	228.97	46.73	-	0.20	6.77	4.27
$\mathbf{CrO_2MnSe_2}$	2.84	P-3m1	163.59	81.91	218.33	245.51	81.68	-	0.33	5.90	3.41
$\mathbf{CrO_2MnTe_2}$	2.96	P-3m1	185.40	76.96	217.53	262.36	108.45	-	0.41	5.51	2.99
$\mathbf{CrS_2MnO_2}$	2.84	P-3m1	187.21	90.58	244.18	277.79	96.62	-	0.35	7.58	4.33
$\mathbf{CrS_2MnSe_2}$	3.10	P-3m1	103.23	58.10	148.71	161.33	45.13	-	0.28	4.95	2.97
$\mathbf{CrS_2MnTe_2}$	3.19	P-3m1	108.14	54.39	144.75	162.53	53.76	-	0.33	4.50	2.60
$\mathbf{CrSe_2MnO_2}$	2.90	P-3m1	182.68	68.09	198.41	250.77	114.59	-	0.46	6.09	3.17
$\mathbf{CrSe_2MnS_2}$	3.12	P-3m1	141.47	58.22	164.99	199.69	83.25	-	0.42	5.56	3.00
$\mathbf{CrSe_2MnTe_2}$	3.30	Cm	88.18	44.09	117.57	132.27	44.09	-	0.33	3.80	2.19
$\mathbf{CrTe_2MnO_2}$†	3.02	P-3m1	47.07	52.99	99.71	100.06	-5.93	-	-0.06	3.47	2.53
$\mathbf{CrTe_2MnS_2}$	3.25	P-3m1	128.33	47.72	139.15	176.06	80.61	-	0.46	4.77	2.48
$\mathbf{CrTe_2MnSe_2}$	3.34	P-3m1	69.74	45.64	110.35	115.38	24.10	-	0.21	3.59	2.26
$\mathbf{CrO_2MoS_2}$	2.91	P-3m1	190.25	116.71	289.34	306.96	73.54	-	0.24	7.44	4.59
$\mathbf{CrO_2MoSe_2}$	2.96	P-3m1	175.80	85.87	230.76	261.67	89.93	-	0.34	5.94	3.40
$\mathbf{CrS_2MoO_2}$	2.90	P-3m1	228.87	123.03	320.07	351.90	105.84	-	0.30	7.95	4.70
$\mathbf{CrS_2MoSe_2}$	3.15	P-3m1	150.79	97.07	236.21	247.86	53.72	-	0.22	5.89	3.69
$\mathbf{CrS_2MoTe_2}$	3.26	Cm	131.50	72.28	186.57	203.77	59.22	-	0.29	4.92	2.93
$\mathbf{CrSe_2MoO_2}$	2.96	-	212.01	102.91	277.13	314.93	109.10	-	0.35	6.52	3.73
$\mathbf{CrSe_2MoS_2}$	3.16	P-3m1	155.71	93.60	233.84	249.32	62.11	-	0.25	5.93	3.63
$\mathbf{CrSe_2MoTe_2}$	3.35	Cm	91.24	68.90	157.02	160.13	22.34	-	0.14	4.09	2.68
$\mathbf{CrTe_2MoO_2}$	3.06	P-3m1	184.28	89.02	240.10	273.30	95.25	-	0.35	5.53	3.16
$\mathbf{CrTe_2MoS_2}$	3.28	P-3m1	140.28	74.44	194.53	214.72	65.85	-	0.31	5.07	2.99
$\mathbf{CrTe_2MoSe_2}$	3.35	Cm	87.16	73.97	160.05	161.13	13.18	-	0.08	4.10	2.78
$\mathbf{CrO_2PbS_2}$	2.86	P-3m1	158.87	2.83	11.13	161.70	156.06	-	0.96	4.41	0.58
$\mathbf{CrO_2PbSe_2}$	2.90	P-3m1	198.62	24.10	85.98	222.73	174.52	-	0.78	4.66	1.53
$\mathbf{CrO_2PbTe_2}$	3.02	P-3m1	159.70	29.90	100.75	189.60	129.80	-	0.68	4.07	1.62
$\mathbf{CrS_2PbO_2}$	3.16	P-3m1	153.03	74.10	199.71	227.13	78.93	-	0.35	5.77	3.29
$\mathbf{CrS_2PbSe_2}$	3.13	Cm	76.20	8.57	30.82	84.77	67.63	-	0.08	3.00	0.95
$\mathbf{CrS_2PbTe_2}$	3.22	P-3m1	99.68	11.03	39.73	110.71	88.65	-	0.80	3.22	1.02
$\mathbf{CrSe_2PbO_2}$	3.42	P-3m1	42.19	31.70	72.41	73.90	10.49	-	0.14	3.16	2.07
$\mathbf{CrSe_2PbS_2}$	3.51	P-3m1	96.78	27.97	86.80	124.75	68.81	-	0.55	4.08	1.93
$\mathbf{CrTe_2PbO_2}$	3.56	P-3m1	81.71	20.09	64.51	101.00	61.61	-	0.61	3.51	1.56
$\mathbf{CrTe_2PbS_2}$	4.00	P-3m1	42.42	16.03	46.54	58.46	26.39	-	0.45	2.91	1.52
$\mathbf{CrTe_2PbSe_2}$	3.54	P-3m1	22.16	13.20	33.10	35.36	8.96	-	0.25	1.86	1.13
$\mathbf{CrO_2SiS_2}$	2.92	P-3m1	113.30	68.64	170.98	181.95	44.66	-	0.25	6.77	4.16
$\mathbf{CrO_2SiSe_2}^{\dagger}$	2.92	P-3m1	24.33	43.76	62.54	68.09	-19.43	-	-0.29	3.35	2.68
$\mathbf{CrS_2SiO_2}$	2.82	P-3m1	222.48	156.48	367.47	378.96	65.99	-	0.17	9.44	6.07
$\mathbf{CrS_2SiSe_2}^{\dagger}$	3.16	P-3m1	42.29	56.35	96.64	98.64	-14.05	-	-0.14	4.13	3.12
$\mathbf{CrS_2SiTe_2}$	3.28	P-3m1	118.03	30.37	96.61	148.39	87.66	-	0.59	4.56	2.06

Continued on next page

TABLE S3 – Continued from previous page

Material	a(b)	SG	K	G	Y^{2D}	$C_{11}(C_{22})$	C_{12}	C_{66}	ν	V_l	V_t
**$CrSe_2SiO_2$**	2.88	P-3m1	192.95	97.56	259.19	290.51	95.39	-	0.33	6.82	3.95
**$CrSe_2SiS_2$**	3.19	P-3m1	179.17	101.97	259.94	281.14	77.20	-	0.27	7.03	4.23
**$CrSe_2SiTe_2$**	3.44	Cm	96.41	24.89	79.13	121.30	71.52	-	0.59	3.90	1.77
**$CrTe_2SiO_2$**	3.01	P-3m1	171.75	60.06	178.00	231.82	111.69	-	0.48	5.46	2.78
**$CrTe_2SiS_2$**	3.31	Cm	138.79	41.46	127.70	180.25	97.32	-	0.54	5.08	2.44
**GeO_2CrS_2**	2.91	P-3m1	155.03	97.43	239.33	252.47	57.60	-	0.23	7.11	4.42
**$GeSe_2CrTe_2$**	3.63	Cm	46.00	29.51	71.91	75.51	16.49	-	0.22	3.11	1.94
**GeO_2HfS_2**	3.18	P-3m1	133.00	16.34	58.21	149.34	116.66	-	0.78	4.75	1.57
**$GeTe_2HfSe_2$**	3.58	Cm	92.10	30.83	92.39	122.93	61.27	-	0.50	3.52	1.76
**GeO_2MnS_2**	2.93	P-3m1	145.12	84.34	213.35	229.45	60.78	-	0.26	6.77	4.11
**GeS_2MnTe_2**	3.43	P-3m1	104.39	36.85	108.94	141.24	67.54	-	0.48	4.41	2.25
**GeO_2MoS_2**	2.99	P-3m1	209.90	112.75	293.41	322.66	97.15	-	0.30	7.54	4.46
**GeO_2MoTe_2**	3.16	P-3m1	138.55	77.05	198.05	215.59	61.50	-	0.29	4.96	2.96
**$GeTe_2MoS_2$**	3.23	Cm	102.30	1.65	6.49	103.94	100.65	-	0.97	3.40	0.43
**GeO_2SiS_2**	3.01	P-3m1	153.89	96.16	236.72	250.05	57.74	-	0.23	7.76	4.81
**$GeSe_2WO_2$**	2.90	P-3m1	193.23	13.86	51.72	207.09	179.37	-	0.87	4.51	1.17
**HfO_2CrS_2**	3.07	P-3m1	206.97	89.28	249.49	296.25	117.70	-	0.40	6.67	3.66
**HfO_2GeS_2**	3.18	P-3m1	222.40	114.18	301.79	336.59	108.22	-	0.32	7.16	4.17
**HfS_2GeSe_2**	3.59	P-3m1	72.84	37.13	98.37	109.96	35.71	-	0.32	3.95	2.29
**$HfTe_2GeSe_2$**	4.01	Cm	58.03	19.07	57.42	77.10	38.95	-	0.51	3.12	1.55
**HfO_2MnS_2**	3.09	P-3m1	98.09	73.40	167.93	171.48	24.69	-	0.14	5.08	3.32
**HfS_2MnTe_2**	3.50	P-3m1	111.89	34.92	106.47	146.82	76.97	-	0.52	4.12	2.01
**$HfTe_2MnO_2$**	3.04	P-3m1	101.07	36.98	108.29	138.04	64.09	-	0.46	3.57	1.85
**HfS_2MoO_2**	3.12	P-3m1	135.64	13.95	50.59	149.59	121.69	-	0.81	4.52	1.38
**$HfSe_2MoS_2$**	3.38	Cm	115.65	34.41	106.08	150.06	81.24	-	0.54	4.24	2.03
**$HfTe_2MoS_2$**	3.28	Cm	104.53	39.95	115.62	144.48	64.58	-	0.45	3.70	1.94
**HfO_2PbS_2**	3.23	P-3m1	144.15	48.46	145.07	192.61	95.70	-	0.50	4.66	2.34
**$HfTe_2PbS_2$**	4.04	Cm	61.62	19.62	59.53	81.25	42.00	-	0.52	3.13	1.54
**HfO_2SiS_2**†	3.14	P-3m1	107.11	115.94	222.70	223.05	-8.84	-	-0.04	6.15	4.44
**HfS_2SnO_2**	3.28	P-3m1	157.74	66.29	186.70	224.03	91.46	-	0.41	5.66	3.08
**$HfTe_2TiO_2$**	3.11	P-3m1	106.45	51.36	138.57	157.80	55.09	-	0.35	3.94	2.25
**HfO_2WS_2**	3.12	P-3m1	224.60	114.54	303.43	339.14	110.05	-	0.32	6.12	3.56
**HfS_2WTe_2**	3.51	Cm	113.21	60.96	158.50	174.17	52.24	-	0.30	4.06	2.40
**MnO_2CrS_2**	2.84	P-3m1	186.58	90.38	243.55	276.96	96.19	-	0.35	7.57	4.33
**MnS_2HfO_2**	3.09	P-3m1	132.45	71.57	185.86	204.03	60.88	-	0.30	5.54	3.28
**MnO_2MoS_2**	2.92	P-3m1	190.15	103.47	268.03	293.62	86.68	-	0.30	7.27	4.32
**$MnTe_2PbSe_2$**	3.96	P-3m1	53.09	18.82	55.59	71.92	34.27	-	0.48	2.95	1.51
**$MnTe_2SnO_2$**	3.22	P-3m1	129.76	64.55	172.43	194.31	65.21	-	0.34	4.78	2.75
**MoS_2GeO_2**	2.99	P-3m1	216.04	85.90	245.84	301.93	130.14	-	0.43	7.30	3.89
**$MoSe_2GeO_2$**	3.05	P-3m1	185.13	108.41	273.49	293.54	76.71	-	0.26	6.29	3.82
**MoS_2HfSe_2**	3.38	Cm	115.49	34.54	106.36	150.03	80.95	-	0.54	4.24	2.03
**$MoSe_2HfTe_2$**	3.39	P-3m1	67.03	17.94	56.61	84.97	49.09	-	0.58	2.72	1.25
**$MoSe_2SiTe_2$**	3.40	P-3m1	121.05	37.67	114.92	158.72	83.38	-	0.53	4.22	2.06
**$MoTe_2WSe_2$**	3.39	Cm	127.00	86.54	205.87	213.54	40.45	-	0.19	4.30	2.74
**PbO_2GeTe_2**	3.66	Cm	47.89	10.30	33.91	58.19	37.59	-	0.65	2.68	1.13
**PbO_2MoS_2**	3.21	P-3m1	164.87	85.00	225.72	250.66	79.07	-	0.32	5.82	3.40
**$PbTe_2WSe_2$**	3.38	P-3m1	81.20	17.06	56.39	98.26	64.14	-	0.65	2.70	1.12
**SiO_2GeTe_2**	2.97	P-3m1	132.87	6.46	24.66	139.34	126.41	-	0.91	4.07	0.88
**$SiSe_2MoS_2$**	3.27	P-3m1	124.97	63.07	167.66	188.04	61.90	-	0.33	5.50	3.18
**$SiTe_2WS_2$**	3.32	P-3m1	140.60	49.73	146.95	190.34	90.87	-	0.48	4.54	2.32
**SnO_2GeS_2**	3.20	P-3m1	175.89	91.70	241.10	267.59	84.19	-	0.31	7.06	4.13
**SnO_2MnS_2**	3.09	P-3m1	125.43	69.35	178.64	194.79	56.08	-	0.29	5.99	3.57
**SnO_2PbS_2**	3.53	Cm	50.43	4.48	16.45	54.91	45.96	-	0.84	2.91	0.83
**SnO_2TiTe_2**	3.22	P-3m1	138.09	39.21	122.16	177.30	98.88	-	0.56	4.60	2.16
**$SnTe_2WSe_2$**	3.35	Cm	79.37	14.49	49.02	93.86	64.88	-	0.69	2.77	1.09
**TiO_2GeS_2**†	3.01	P-3m1	61.90	71.17	132.42	133.07	-9.27	-	-0.07	5.38	3.94
**TiO_2MoS_2**	3.00	P-3m1	171.80	98.68	250.71	270.48	73.12	-	0.27	7.28	4.40
**$TiSe_2SiO_2$**	2.85	P-3m1	152.70	50.22	151.17	202.93	102.48	-	0.51	5.69	2.83
**$TiTe_2SnSe_2$**	3.99	Cm	55.35	21.91	62.79	77.27	33.44	-	0.43	3.33	1.77
**WSe_2GeO_2**	3.06	P-3m1	196.70	123.70	303.76	320.40	73.00	-	0.23	5.91	3.67

Continued on next page

TABLE S3 – Continued from previous page

Material	a(b)	SG	K	G	Y^{2D}	$C_{11}(C_{22})$	C_{12}	C_{66}	ν	V_l	V_t
$\mathbf{WO_2MnS_2}$	2.90	P-3m1	184.90	103.29	265.09	288.19	81.60	-	0.28	6.14	3.67
$\mathbf{WSe_2MoS_2}$	3.22	P-3m1	165.63	112.18	267.53	277.81	53.45	-	0.19	5.46	3.47
$\mathbf{WTe_2PbSe_2}$	3.57	P-3m1	76.31	29.44	84.98	105.75	46.87	-	0.44	2.95	1.56
$\mathbf{WTe_2TiSe_2}$	3.49	Cm	98.83	59.27	148.20	158.10	39.56	-	0.25	3.94	2.41
**Heterostructure with one 2H and one 1T monolayer**											
$\mathbf{CrO_2/GeS_2}$	3.52(3.13)	P1	593.61/56.11	40.01/24.31	149.93/67.85	103.66(229.06)	90.57	69.64	0.84/0.40	12.60/4.49	3.17/2.47
$\mathbf{CrO_2/GeSe_2}^\dagger$	2.84(3.81)	P1	27.95/63.95	32.06/90.44	59.73/149.84	151.62(60.44)	-10.37	33.45	-0.07/-0.17	3.03/4.86	2.22/3.72
$\mathbf{CrO_2/GeTe_2}$	3.94(3.01)	P1	275.81/177.56	12.19	46.19	228.84	204.39	144.00	0.91/0.87	6.14/4.98	1.26
$\mathbf{CrO_2/HfTe_2}$	3.14(3.72)	P1	59.75/172.93	32.82/51.24	84.73/158.12	187.77(100.62)	54.62	44.43	0.29/0.54	3.19/4.96	1.90/2.37
$\mathbf{CrO_2/MnS_2}$	3.05(2.91)	P1	165.22/60.38	30.26/22.96	102.31/66.54	96.43(148.27)	66.57	12.94	0.69/0.45	6.68/4.36	2.63/2.29
$\mathbf{CrO_2/MnSe_2}$	3.01(3.31)	P1	35.00/442.87	13.39/20.66	38.73/78.96	133.14(65.31)	59.49	32.61	0.45/0.91	2.87/8.89	1.51/1.88
$\mathbf{CrO_2/MoTe_2}$	3.53(3.19)	P1	494.11/47.02	56.74/27.13	203.60/68.81	87.44(258.72)	69.42	91.12	0.79/0.27	8.46/3.11	2.72/1.88
$\mathbf{CrO_2/PbS_2}$	3.04(3.99)	P1	12.30/926.12	6.77/14.81	17.46/58.29	81.06(24.28)	23.15	19.91	0.29/0.97	1.72/12.09	1.03/1.52
$\mathbf{CrO_2/PbSe_2}$	3.89(2.94)	P1	558.69/85.44	10.40	39.13	164.80	143.25	99.64	0.96/0.79	8.10/3.31	1.10
$\mathbf{CrO_2/SiS_2}$	3.09(3.22)	P1	97.64/173.65	27.49	90.78	155.05	99.32	22.61	0.58/0.71	5.99/7.68	2.83
$\mathbf{CrO_2/SnS_2}$	3.26(3.71)	P1	78.94/96.88	58.86	140.98	146.61	28.61	46.16	0.20	5.74	3.64
$\mathbf{CrO_2/SnSe_2}$	3.81(2.93)	P1	458.47/81.14	14.72	61.14/46.67	139.28(182.46)	129.99	65.96	0.93(0.71)	8.19(3.66)	1.46
$\mathbf{CrO_2/SnTe_2}$	3.04(3.99)	P1	159.09/177.46	111.91	268.83	280.10	56.13	55.47	0.20	5.80	3.67
$\mathbf{CrO_2/TiS_2}$	3.04(3.31)	P1	65.16/115.37	16.87/19.01	53.60/65.29	112.98(92.75)	66.51	28.11	0.59/0.72	4.62/5.91	2.09/2.22
$\mathbf{CrO_2/TiSe_2}$	2.80(3.28)	P1	94.72/73.34	54.32/46.54	138.09/113.89	121.23(147.00)	32.86	13.07	0.27/0.22	5.07/4.55	3.06/2.84
$\mathbf{CrO_2/TiTe_2}$	3.12(3.72)	P1	60.72/274.32	24.39/35.50/	69.61/125.72	187.37(103.74)	79.97	31.83	0.43/0.77	3.51/6.70	1.88/2.27
$\mathbf{CrO_2/WTe_2}$	3.49(3.20)	P1	275.74/62.18	76.61/39.20	239.81/96.18	110.31(275.04)	62.34	92.68	0.57/0.23	6.15/3.30	2.87/2.05
$\mathbf{CrS_2/GeO_2}$	2.96	P1	152.17	115.56	262.73	267.73	36.61	-	0.14	7.45	4.90
$\mathbf{CrS_2/GeSe_2}$	3.21	P1	91.99	22.11	71.30	114.10	69.88	-	0.61	4.21	1.85
$\mathbf{CrS_2/HfO_2}^\dagger$	3.07	P1	79.34	103.18	179.41	182.52	-23.84	-	-0.13	5.25	3.95
$\mathbf{CrS_2/MnO_2}$	2.83	P1	164.93	96.23	243.08	261.16	68.71	-	0.26	7.33	4.45
$\mathbf{CrS_2/MnSe_2}$	3.14	P1	78.86	51.58	124.73	130.43	27.28	-	0.21	4.51	2.83
$\mathbf{CrS_2/MnTe_2}$	3.26	P1	69.09	35.73	94.20	104.82	33.36	-	0.32	3.68	2.15
$\mathbf{CrS_2/MoO_2}$	2.89	P1	204.12	129.83	317.42	333.94	74.29	-	0.22	7.73	4.82
$\mathbf{CrS_2/MoSe_2}$	3.19	P1	116.09	85.23	196.59	201.32	30.86	-	0.15	5.37	3.49
$\mathbf{CrS_2/PbO_2}$	3.18	P1	123.37	67.40	174.34	190.76	55.97	-	0.29	5.33	3.17
$\mathbf{CrS_2/PbSe_2}$	3.15	P1	43.44	5.82	20.54	49.26	37.61	-	0.76	2.30	0.79
$\mathbf{CrS_2/PbTe_2}$	3.07(3.33)	P1	77.70/155.36	47.85/69.12	118.45/191.35	210.59(130.35)	50.08	24.16	0.24/0.38	3.45/4.61	2.13/2.56
$\mathbf{CrS_2/SiO_2}$	2.85	P1	170.03	120.50	282.08	290.53	49.53	-	0.17	8.34	5.37
$\mathbf{CrS_2/SiSe_2}^\dagger$	3.25	P1	4.39	32.60	15.47	36.99	-28.21	-	-0.76	2.60	2.44
$\mathbf{CrS_2/SiTe_2}$	3.17	P1	96.77	4.38	16.77	101.16	92.39	-	0.91	3.64	0.76
$\mathbf{CrS_2/SnO_2}$	3.07	P1	111.04	98.71	209.03	209.75	12.32	-	0.06	6.22	4.27
$\mathbf{CrS_2/TiO_2}$	3.00	P1	144.51	108.42	247.77	252.92	36.09	-	0.14	7.78	5.10
$\mathbf{CrS_2/TiTe_2}$	3.25(3.47)	P1	52.47	16.04	49.35	69.48	37.40	25.58	0.54	3.10	1.49
$\mathbf{CrS_2/WO_2}$	2.90	P1	219.19	142.74	345.78	361.93	76.46	-	0.21	6.90	4.33
$\mathbf{CrS_2/WSe_2}$	3.19	P1	121.37	93.51	211.27	214.88	27.86	-	0.13	4.99	3.29
$\mathbf{CrSe_2/GeO_2}$	3.02	P1	142.73	92.58	224.62	235.31	50.15	-	0.21	5.97	3.75
$\mathbf{CrSe_2/GeS_2}$	3.33	P1	84.47	62.58	143.80	147.06	21.89	-	0.15	4.96	3.23
$\mathbf{CrSe_2/GeTe_2}$	3.23	P1	66.58	2.06	7.99	68.64	64.52	-	0.94	2.66	0.46
$\mathbf{CrSe_2/HfO_2}^\dagger$	3.12	P1	77.51	100.64	175.15	178.15	-23.13	-	-0.13	4.64	3.48
$\mathbf{CrSe_2/HfS_2}$	3.38	P1	105.90	52.74	140.82	158.64	53.17	-	0.34	4.57	2.64
$\mathbf{CrSe_2/HfTe_2}$	3.34(3.61)	P1	57.21/38.22	16.96/14.78	52.33/42.64	56.11(68.85)	30.44	16.18	0.54/0.44	2.72/2.30	1.30/1.22
$\mathbf{CrSe_2/MnO_2}$	2.90	P1	165.34	69.02	194.77	234.36	96.32	-	0.41	5.87	3.19
$\mathbf{CrSe_2/MnS_2}$	3.12	P1	118.28	59.61	158.54	177.89	58.67	-	0.33	5.24	3.03
$\mathbf{CrSe_2/MnTe_2}$	3.34	P1	46.10	36.50	81.48	82.59	9.60	-	0.12	3.04	2.02
$\mathbf{CrSe_2/MoO_2}$	2.95	P1	197.35	108.08	279.35	305.44	89.27	-	0.29	6.40	3.81
$\mathbf{CrSe_2/MoS_2}$	3.16	P1	133.12	95.29	222.14	228.41	37.83	-	0.17	5.67	3.66
$\mathbf{CrSe_2/MoTe_2}$	3.38	P1	87.11	61.99	144.87	149.10	25.11	-	0.17	3.98	2.56
$\mathbf{CrSe_2/PbO_2}$	3.24	P1	90.99	70.19	158.50	161.19	20.80	-	0.13	4.43	2.92
$\mathbf{CrSe_2/PbS_2}$	3.53	P1	63.44	24.72	71.28	88.56	39.13	-	0.44	3.46	1.83
$\mathbf{CrSe_2/SiO_2}$	2.91	P1	165.49	85.52	225.53	251.01	79.97	-	0.32	6.40	3.74
$\mathbf{CrSe_2/SiS_2}$	3.20	P1	122.87	72.01	181.61	194.89	50.86	-	0.26	5.87	3.57
$\mathbf{CrSe_2/SiTe_2}$	3.19	P1	103.24	41.80	119.02	145.04	61.44	-	0.42	3.98	2.14
$\mathbf{CrSe_2/SnO_2}^\dagger$	3.12	P1	81.55	93.57	174.29	175.12	-12.02	-	-0.07	4.97	3.63
$\mathbf{CrSe_2/SnS_2}$	3.48	P1	96.57	46.91	126.29	143.48	49.66	-	0.35	4.82	2.75

Continued on next page

TABLE S3 – Continued from previous page

Material	a(b)	SG	K	G	Y^{2D}	$C_{11}(C_{22})$	C_{12}	C_{66}	ν	V_l	V_t
**$CrSe_2/TiO_2$**	3.06	P1	129.44	89.42	211.54	218.86	40.02	-	0.18	6.06	3.88
**$CrSe_2/TiS_2$**	3.24	P1	108.98	63.96	161.21	172.94	45.02	-	0.26	5.43	3.30
**$CrSe_2/TiTe_2$**	3.36(3.53)	P1	50.79/58.74	20.48/21.66	58.38/63.30	78.75(72.62)	33.49	8.35	0.43/0.46	2.95/3.13	1.58/1.63
**$CrSe_2/WO_2$**	2.94	P1	217.59	120.39	310.03	337.98	97.19	-	0.29	5.98	3.57
**$CrSe_2/WS_2$**	3.16	P1	140.67	103.48	238.48	244.15	37.19	-	0.15	5.27	3.43
**$CrSe_2/WTe_2$**	3.38	P1	91.12	67.91	155.64	159.02	23.21	-	0.15	3.82	2.50
**$CrTe_2/GeO_2$**	3.15	P1	143.26	63.40	175.80	206.67	79.86	-	0.39	5.09	2.82
**$CrTe_2/GeSe_2$**	3.54	P1	88.32	36.72	103.75	125.04	51.59	-	0.41	3.89	2.11
**$CrTe_2/HfO_2$**	3.30	P1	145.12	95.70	230.69	240.83	49.42	-	0.21	5.13	3.24
**$CrTe_2/HfS_2$**	3.49	P1	106.56	50.41	136.89	156.97	56.14	-	0.36	4.26	2.42
**$CrTe_2/HfSe_2$**	3.55	P1	100.26	46.02	126.17	146.28	54.24	-	0.37	3.87	2.17
**$CrTe_2/MnO_2$**†	3.01	P1	48.47	53.44	101.68	101.92	-4.97	-	-0.05	3.50	2.53
**$CrTe_2/MnS_2$**	3.23	P1	124.87	50.19	143.20	175.06	74.68	-	0.43	4.73	2.53
**$CrTe_2/MnSe_2$**	3.32	P1	104.44	44.02	123.87	148.46	60.42	-	0.41	4.05	2.20
**$CrTe_2/MoO_2$**	3.04	P1	200.58	87.65	243.99	288.23	112.92	-	0.39	5.66	3.12
**$CrTe_2/MoS_2$**	3.26	P1	131.11	77.41	194.70	208.53	53.70	-	0.26	4.97	3.03
**$CrTe_2/MoSe_2$**	3.34	P1	79.50	75.46	154.86	154.96	4.04	-	0.03	4.01	2.80
**$CrTe_2/PbO_2$**†	3.33	P1	21.86	57.67	63.40	79.53	-35.82	-	-0.45	2.90	2.47
**$CrTe_2/PbS_2$**	3.75	P1	24.35	17.34	40.51	41.69	7.01	-	0.17	2.30	1.48
**$CrTe_2/PbSe_2$**	3.76	P1	29.14	9.40	28.43	38.54	19.74	-	0.51	2.06	1.02
**$CrTe_2/SiS_2$**	3.39(3.74)	P1	69.46/114.79	45.98/62.24	110.66/161.44	171.81(117.77)	34.96	39.20	0.20/0.30	4.29/5.32	2.71/3.15
**$CrTe_2/SnO_2$**	3.30	P1	120.01	73.86	182.88	193.87	46.15	-	0.24	4.91	3.03
**$CrTe_2/SnS_2$**	3.57	P1	109.75	49.64	136.73	159.40	60.11	-	0.38	4.65	2.59
**GeO_2/MnS_2**	2.99	P1	145.61	67.82	185.07	213.43	77.79	-	0.36	6.68	3.76
**GeO_2/MoS_2**	3.04	P1	179.51	113.17	277.64	292.68	66.34	-	0.23	7.31	4.54
**$GeO_2/MoSe_2$**	3.10	P1	164.31	99.10	247.27	263.41	65.20	-	0.25	6.07	3.72
**GeO_2/PbS_2**	3.02	P1	116.03	16.69	58.37	132.72	99.34	-	0.75	4.10	1.45
**GeS_2/MoO_2**	3.07	P1	194.66	79.34	225.46	273.99	115.32	-	0.42	7.13	3.84
**GeS_2/SnO_2**	3.31	P1	142.07	80.27	205.17	22.35	61.80	-	0.28	6.64	3.99
**$GeTe_2/WSe_2$**	3.46	P1	60.85	31.07	82.28	91.92	29.78	-	0.32	2.93	1.70
**HfO_2/MoS_2**	3.19	P1	193.25	119.26	294.99	312.51	73.99	-	0.24	6.70	4.14
**$HfO_2/MoSe_2$**	3.25	P1	179.89	117.65	284.51	297.54	62.25	-	0.21	5.94	3.73
**HfS_2/MoO_2**	3.11	P1	136.54	56.54	159.94	193.09	80.00	-	0.41	5.12	2.77
**$HfS_2/PbSe_2$**	3.63	P1	39.77	35.58	75.11	75.34	4.19	-	0.06	2.92	2.01
**HfS_2/SnO_2**	3.39	P1	122.79	70.50	179.14	193.29	52.30	-	0.27	5.43	3.28
**MnO_2/CrS_2**	2.94	P1	125.93	45.31	133.28	171.24	80.62	-	0.47	6.16	3.17
**MnO_2/GeS_2**	3.16	P1	152.44	38.32	122.49	190.76	114.12	-	0.60	6.66	2.98
**MnO_2/MoS_2**	3.04	P1	157.56	81.46	214.78	239.01	76.10	-	0.32	6.82	3.98
**MnS_2/HfO_2**†	3.09	P1	59.83	76.23	134.09	136.06	-16.40	-	-0.12	4.54	3.40
**MnS_2/MoO_2**	2.88	P1	174.59	105.21	262.59	279.80	69.38	-	0.25	7.01	4.30
**$MnTe_2/PbSe_2$**	3.96	P1	32.47	17.45	45.41	49.93	15.02	-	0.30	2.46	1.45
**$MnTe_2/SnO_2$**	3.29	P1	99.96	62.37	153.62	162.33	37.59	-	0.23	4.47	2.77
**MoS_2/GeO_2**	3.01	P1	164.87	125.86	285.49	290.72	39.01	-	0.13	7.21	4.74
**$MoS_2/HfSe_2$**	3.44	P1	88.57	42.15	114.23	130.72	46.43	-	0.36	4.03	2.29
**MoS_2/SiO_2**	2.91	P1	185.26	114.55	283.13	299.81	70.71	-	0.24	7.75	4.79
**$MoSe_2/GeO_2$**	3.07	P1	150.05	98.71	238.17	248.76	51.34	-	0.21	5.84	3.68
**$MoSe_2/SiTe_2$**	3.40	P1	91.89	44.11	119.22	136.01	47.78	-	0.35	3.91	2.23
**$MoTe_2/HfSe_2$**	3.57	P1	95.47	59.29	146.30	154.76	36.18	-	0.23	3.87	2.39
**$PbO_2/GeTe_2$**	3.46	P1	68.50	30.31	84.05	98.81	38.19	-	0.39	3.30	1.83
**PbO_2/HfS_2**	3.44	P1	128.64	62.39	168.06	191.03	66.25	-	0.35	4.95	2.83
**PbO_2/MnS_2**	3.25	P1	128.20	55.62	155.16	183.81	72.58	-	0.39	5.31	2.92
**PbO_2/MoS_2**	3.27	P1	148.67	79.95	207.96	228.62	68.72	-	0.30	5.65	3.34
**$PbTe_2/WSe_2$**	3.33	P1	119.43	21.18	71.96	140.61	98.25	-	0.70	3.19	1.24
**SiO_2/MnS_2**	2.84	P1	130.90	86.75	208.69	217.65	44.15	-	0.20	7.16	4.52
**SiO_2/MoS_2**	2.94	P1	200.39	121.49	302.54	321.88	78.90	-	0.25	8.12	4.99
**SiO_2/PbS_2**	2.87	P1	152.26	7.32	27.95	159.58	144.93	-	0.91	4.60	0.98
**SiO_2/WS_2**	2.95	P1	209.40	129.54	320.12	338.94	79.86	-	0.24	7.07	4.37
**$SiSe_2/HfO_2$**	3.33	P1	166.30	97.11	245.24	263.41	69.19	-	0.26	6.20	3.77
**$SiTe_2/HfSe_2$**	3.68	P1	102.90	35.93	106.51	138.82	66.97	-	0.48	3.98	2.02
**$SiTe_2/WS_2$**	3.42	P1	93.09	32.47	96.29	125.55	60.62	-	0.48	3.79	1.93

Continued on next page

TABLE S3 – Continued from previous page

Material	a(b)	SG	K	G	Y^{2D}	$C_{11}(C_{22})$	C_{12}	C_{66}	ν	V_l	V_t
**SnO_2/GeS_2**	3.31	P1	144.11	81.32	207.94	225.43	62.80	-	0.28	6.69	4.02
**$SnO_2/GeSe_2$**	3.27	P1	127.42	42.69	127.91	170.11	84.73	-	0.50	4.99	2.50
**SnO_2/HfS_2**	3.35	P1	142.07	67.77	183.53	209.84	74.30	-	0.35	5.59	3.18
**SnO_2/MnS_2**	3.16	P1	156.55	68.74	191.07	225.30	87.81	-	0.39	6.59	3.64
**SnO_2/MoS_2**	3.19	P1	171.83	101.69	255.53	273.52	70.14	-	0.26	6.82	4.16
**SnO_2/PbS_2**†	3.43	P1	23.87	40.11	59.86	63.98	-16.24	-	-0.25	3.05	2.42
**$SnTe_2/HfSe_2$**	3.84	P1	59.48	22.88	66.09	82.36	36.60	-	0.44	2.98	1.57
**$SnTe_2/MnS_2$**	3.30(4.00)	P1	198.69/157.24	43.35/40.99	142.35/130.06	208.55(228.24)	133.84	71.01	0.64/0.59	5.10/4.62	2.13
**$SnTe_2/WO_2$**	3.02	P1	224.85	45.31	150.85	270.16	179.54	-	0.66	4.67	1.91
**TiO_2/MnS_2**	3.02	P1	146.17	64.74	179.46	210.90	81.43	-	0.39	7.11	3.94
**TiO_2/MoS_2**	3.07	P1	158.78	103.61	250.80	262.39	55.17	-	0.21	7.33	4.60
**TiO_2/SnS_2**	3.54(3.36)	P1	272.47/43.17	37.31/21.60	131.27/57.59	77.07(175.68)	58.50	36.84	0.76/0.33	8.45/3.86	2.93/2.23
**TiO_2/WS_2**	3.07	P1	169.56	114.65	273.60	284.21	54.91	-	0.19	6.52	4.14
**$TiSe_2/MoS_2$**	3.27	P1	121.32	57.37	155.80	178.69	63.95	-	0.36	5.22	2.96
**$TiTe_2/SnSe_2$**	3.79	P1	60.24	35.86	89.91	96.10	24.39	-	0.25	3.52	2.15
**WO_2/GeS_2**	3.18	P1	50.05	44.83	94.60	94.88	5.22	-	0.06	3.77	2.59
**$WO_2/MoSe_2$**	3.10	P1	142.68	81.85	208.05	224.53	60.83	-	0.27	4.89	2.95
**WO_2/SiS_2**	3.10	P1	144.68	81.64	208.77	226.33	63.04	-	0.28	6.06	3.64
**$WO_2/TiTe_2$**	3.12	P1	114.19	35.20	107.62	149.39	78.99	-	0.53	3.83	1.86
**WS_2/PbO_2**	3.23	P1	133.60	86.69	210.30	220.29	46.92	-	0.21	4.96	3.11
**WS_2/SnO_2**†	3.13	P1	86.04	114.46	196.47	200.50	-28.42	-	-0.14	5.06	3.82
**$WS_2/TiSe_2$**	3.32	P1	94.68	53.76	137.17	148.45	40.92	-	0.28	4.33	2.61
**WSe_2/GeO_2**	3.07	P1	151.99	98.00	238.34	249.99	53.98	-	0.22	5.25	3.29
**WSe_2/HfS_2**	3.42	P1	114.68	68.54	171.59	183.21	46.14	-	0.25	4.37	2.67
**WSe_2/MoS_2**	3.19	P1	150.21	118.45	264.91	268.66	31.76	-	0.12	5.32	3.53
**WSe_2/PbS_2**	3.55	P1	60.09	41.64	98.38	101.73	18.46	-	0.18	3.30	2.11
**WSe_2/SnS_2**	3.51	P1	94.56	63.49	151.93	158.04	31.07	-	0.20	4.40	2.79
**WTe_2/HfS_2**	3.50	P1	107.12	71.42	171.40	178.54	35.70	-	0.20	4.08	2.58
**$WTe_2/PbSe_2$**	3.83	P1	34.34	19.57	49.87	53.92	14.77	-	0.27	2.27	1.37
**$WTe_2/TiSe_2$**	3.47	P1	88.49	68.24	154.12	156.73	20.24	-	0.13	3.91	2.58
			**Heterostructure with two planar (P) monolayer**								
**BaOBeS**	3.75	P-3m1	36.91	21.13	53.75	58.04	15.78	-	0.27	4.68	2.82
**BaOBeSe**	3.90	P-3m1	34.70	18.19	47.74	52.90	16.51	-	0.31	4.17	2.44
**BaOBeTe**	4.13	P-3m1	46.93	11.34	36.52	58.26	35.59	-	0.61	4.23	1.86
**BaOCdS**	4.26	P-3m1	54.13	4.57	16.86	58.70	49.56	-	0.84	4.32	1.20
**BaOCdSe**	4.33	P-3m1	39.37	7.63	25.56	47.00	31.74	-	0.68	3.65	1.47
**BaOCdTe**	4.41	P-3m1	28.34	9.17	27.72	37.51	19.16	-	0.51	3.11	1.54
**BaOMgS**	4.18	P-3m1	69.84	6.70	24.45	76.54	63.14	-	0.82	5.77	1.71
**BaOMgSe**	4.29	P-3m1	89.16	12.08	42.55	101.24	77.08	-	0.76	6.16	2.13
**BaOMgTe**	4.38	P-3m1	21.43	8.96	25.27	30.38	12.47	-	0.41	3.16	1.71
**BaOSrS**	4.36	P-3m1	7.17	1.87	5.94	9.04	5.30	-	0.59	1.81	0.82
**BaOSrTe**	4.45	P-3m1	31.39	9.14	28.31	40.53	22.25	-	0.55	3.37	1.60
**BaOZnS**	4.08	P-3m1	57.82	11.24	37.64	69.06	46.58	-	0.67	4.89	1.97
**BaOZnSe**	4.19	P-3m1	57.07	19.20	57.47	76.28	37.87	-	0.50	4.84	2.43
**BaOZnTe**	4.33	P-3m1	53.62	13.46	43.03	67.08	40.16	-	0.60	4.35	1.95
**BaSBeSe**	3.95	P-3m1	32.97	13.42	38.15	46.39	19.55	-	0.42	3.83	2.06
**BaSBeTe**	4.26	P-3m1	26.41	12.33	33.62	38.73	14.08	-	0.36	3.46	1.95
**BaSCdSe**	4.60	P-3m1	7.29	3.20	8.90	10.49	4.10	-	0.39	1.79	0.99
**BaSCdTe**	4.82	P-3m1	25.59	2.61	9.48	28.20	22.98	-	0.81	2.89	0.88
**BaSMgO**	3.71	P-3m1	45.64	6.31	22.19	51.95	39.32	-	0.76	4.22	1.47
**BaSSrO**	4.19	P-3m1	31.19	9.44	29.00	40.64	21.75	-	0.54	3.69	1.78
**BaSSrSe**	4.97	P-3m1	12.17	6.19	16.42	18.37	5.98	-	0.33	2.65	1.54
**BaSSrTe**	5.05	P-3m1	10.21	6.71	16.20	16.92	3.50	-	0.21	2.42	1.52
**BaSZnTe**	4.62	P-3m1	39.06	6.02	20.87	45.08	33.04	-	0.73	3.72	1.36
**BaSeBeO**	4.58	P-3m1	22.06	1.85	6.84	23.92	20.21	-	0.84	3.29	0.92
**BaSeBeS**	3.78	P-3m1	11.92	6.58	16.96	18.50	5.34	-	0.29	2.32	1.38
**BaSeBeTe**	4.26	P-3m1	25.94	8.77	26.21	34.70	17.17	-	0.49	3.05	1.53
**BaSeCdO**	3.95	P-3m1	36.56	15.49	43.52	52.05	21.07	-	0.40	3.50	1.91
**BaSeCdS**	4.46	P-3m1	8.72	0.59	2.21	9.31	8.13	-	0.87	1.64	0.41
**BaSeCdTe**	4.86	P-3m1	20.04	3.41	11.65	23.45	16.63	-	0.71	2.52	0.96

Continued on next page

TABLE S3 – Continued from previous page

Material	a(b)	SG	K	G	Y^{2D}	$C_{11}(C_{22})$	C_{12}	C_{66}	ν	V_l	V_t
**BaSeMgO**	3.72	P-3m1	26.89	11.75	32.71	38.64	15.14	-	0.39	3.29	1.82
**BaSeMgS**	4.30	P-3m1	31.21	6.71	22.08	37.92	24.50	-	0.65	3.66	1.54
**BaSeMgTe**	4.80	P-3m1	34.12	1.96	7.41	36.08	32.16	-	0.89	3.43	0.80
**BaSeSrO**	4.22	P-3m1	29.82	11.10	32.36	40.92	18.72	-	0.46	3.44	1.79
**BaSeSrS**	4.92	P-3m1	15.55	6.72	18.76	22.27	8.84	-	0.40	2.89	1.59
**BaSeSrTe**	5.12	P-3m1	7.52	7.01	14.52	14.53	0.51	-	0.04	2.15	1.49
**BrSeZnO**	4.59	P-3m1	23.97	2.83	10.13	26.80	21.14	-	0.79	3.14	1.02
**BaSeZnS**	4.17	P-3m1	31.07	0.67	2.64	31.74	30.39	-	0.96	3.03	0.44
**BaSeZnTe**	4.65	P-3m1	13.67	5.32	15.33	19.00	8.35	-	0.44	2.29	1.21
**BaTeBeS**	4.26	P-3m1	26.59	15.69	39.47	42.28	10.90	-	0.26	3.62	2.20
**BaTeBeSe**	4.02	P-3m1	19.30	0.72	2.78	20.02	18.58	-	0.93	2.19	0.41
**BaTeCdO**	3.96	P-3m1	11.75	2.66	8.67	14.41	9.10	-	0.63	1.73	0.74
**BaTeCdS**	5.25	P-3m1	17.12	1.60	5.84	18.71	15.52	-	0.83	2.56	0.75
**BaTeSrO**	4.30	P-3m1	32.47	11.65	34.31	44.13	20.82	-	0.47	3.39	1.74
**BaTeSrS**	4.97	P-3m1	14.46	7.26	19.33	21.72	7.20	-	0.33	2.70	1.56
**BaTeSrSe**	5.08	P-3m1	9.90	7.23	16.72	17.13	2.67	-	0.16	2.31	1.50
**BaTeZnO**	3.53	P-3m1	50.39	16.83	50.47	67.22	33.56	-	0.50	3.55	1.78
**BaTeZnS**	5.24	P-3m1	17.49	1.51	5.56	19.00	15.98	-	0.84	2.74	0.77
**BaTeZnSe**	5.46	P-3m1	13.67	1.72	6.11	15.39	11.96	-	0.78	2.42	0.81
**BeOBaSe**	4.58	P-3m1	22.27	1.88	6.93	24.15	20.39	-	0.84	3.31	0.92
**BeSBaO**	3.75	P-3m1	36.45	20.98	53.26	57.43	15.47	-	0.27	4.66	2.82
**BeSBaSe**	3.79	P-3m1	11.94	6.53	16.89	18.47	5.41	-	0.29	2.32	1.38
**BeSBaTe**	4.26	P-3m1	26.72	125.70	39.56	42.42	11.02	-	0.26	3.62	2.20
**BeSCdO**	3.61	P-3m1	97.87	24.58	78.59	122.45	73.29	-	0.60	7.01	3.14
**BeSCdSe**	3.62	P-3m1	47.48	15.74	47.28	63.22	31.75	-	0.50	4.31	2.15
**BeSCdTe**	3.38	P-3m1	52.07	6.74	23.88	58.82	45.33	-	0.77	3.53	1.20
**BeSMgO**	3.46	P-3m1	92.12	42.54	116.41	134.66	49.58	-	0.37	10.15	5.70
**BeSMgSe**	3.75	P-3m1	19.54	10.13	26.68	29.67	9.41	-	0.32	3.89	2.27
**BeSMgTe**	3.41	P-3m1	75.45	22.76	69.95	98.21	52.69	-	0.54	5.55	2.67
**BeTeMgO**	3.66	P-3m1	60.81	38.92	94.92	99.73	21.89	-	0.22	6.27	3.92
**BeTeZnS**	3.93	P-3m1	72.80	32.11	89.12	104.91	40.69	-	0.39	6.01	3.33
**CdOBeTe**	3.86	P-3m1	65.01	29.53	81.23	94.55	35.48	-	0.38	5.26	2.94
**CdOZnS**	3.79	P-3m1	77.87	24.17	73.77	102.03	53.70	-	0.53	5.82	2.83
**CdOZnSe**	3.83	P-3m1	62.94	20.49	61.83	83.43	42.45	-	0.51	4.84	2.40
**CdSBaO**	4.26	P-3m1	54.12	4.50	16.60	58.61	49.62	-	0.85	4.31	1.19
**CdSBaTe**	5.25	P-3m1	17.05	1.61	5.89	18.67	15.44	-	0.83	2.56	0.75
**CdSMgO**	3.66	P-3m1	27.63	7.53	23.67	35.16	20.10	-	0.57	3.65	1.69
**CdSeMgO**†	3.49	P-3m1	3.67	10.54	10.88	14.20	-6.87	-	-0.48	1.97	1.70
**CdTeBeSe**	3.71	P-3m1	37.67	7.22	24.23	44.89	30.45	-	0.68	3.14	1.26
**CdTeMgSe**	4.42	P-3m1	36.71	1.68	6.44	38.40	35.03	-	0.91	3.38	0.71
**MgOBaS**	3.71	P-3m1	36.54	7.60	25.17	44.15	28.94	-	0.66	3.89	1.61
**MgOBeSe**	3.55	P-3m1	75.90	40.59	105.79	116.49	35.31	-	0.30	7.72	4.56
**MgOZnS**	3.61	P-3m1	59.68	30.69	81.06	90.36	28.99	-	0.32	6.68	3.89
**MgOZnSe**	3.80	P-3m1	27.66	19.07	45.15	46.73	8.60	-	0.18	4.37	2.79
**MgSBaO**	4.18	P-3m1	69.84	6.72	24.52	76.56	63.12	-	0.82	5.77	1.71
**MgSBeSe**	3.87	P-3m1	54.15	23.16	64.90	77.32	30.99	-	0.40	6.46	3.54
**MgSZnO**	3.62	P-3m1	16.91	0.59	2.26	17.49	16.32	-	0.93	2.94	0.54
**MgSeBaO**	4.28	P-3m1	47.37	3.79	14.05	51.17	43.58	-	0.85	4.37	1.19
**MgSeBeS**	3.75	P-3m1	20.16	11.13	28.69	31.30	9.03	-	0.29	3.99	2.38
**MgSeCdO**	4.00	P-3m1	44.26	14.85	44.48	59.12	29.41	-	0.50	4.61	2.31
**MgSeCdS**	4.25	P-3m1	54.29	15.49	48.22	69.78	38.79	-	0.56	5.15	2.43
**MgSeCdTe**	4.42	P-3m1	36.63	1.68	6.42	38.31	34.95	-	0.91	3.37	0.71
**MgSeZnTe**	4.31	P-3m1	51.76	17.95	53.31	69.71	33.82	-	0.49	4.78	2.42
**MgTeBeS**	3.40	P-3m1	76.21	24.29	73.68	100.50	51.92	-	0.52	5.61	2.76
**MgTeCdSe**	4.50	P-3m1	36.13	13.26	38.79	49.39	22.87	-	0.46	3.90	2.02
**MgTeZnO**	3.43	P-3m1	45.10	16.14	47.55	61.24	28.95	-	0.47	4.01	2.06
**MgTeZnSe**	4.26	P-3m1	37.30	9.73	30.87	47.03	27.57	-	0.59	3.87	1.76
**SrOBaS**	4.18	P-3m1	25.25	10.29	29.25	35.54	14.95	-	0.42	3.44	1.85
**SrOBeSe**	3.88	P-3m1	69.11	19.62	61.13	88.73	49.49	-	0.56	6.03	2.83
**SrOMgS**	4.06	P-3m1	78.18	13.81	46.95	91.99	64.37	-	0.70	7.03	2.72

Continued on next page

TABLE S3 – Continued from previous page

Material	a(b)	SG	K	G	Y^{2D}	$C_{11}(C_{22})$	C_{12}	C_{66}	ν	V_l	V_t
**SrOZnS**	3.97	P-3m1	79.75	18.35	59.68	98.10	61.40	-	0.63	6.33	2.74
**SrOZnSe**	4.05	P-3m1	50.73	14.10	44.13	64.83	36.64	-	0.57	4.73	2.20
**SrSBaSe**	4.92	P-3m1	30.85	0.62	2.42	31.47	30.23	-	0.96	3.44	0.48
**SrSCdSe**	4.59	P-3m1	48.50	3.60	13.42	52.10	44.90	-	0.86	4.29	1.13
**SrSMgSe**	4.51	P-3m1	47.47	3.76	13.94	51.23	43.71	-	0.85	4.93	1.34
**SrSZnSe**	4.33	P-3m1	28.46	3.21	11.53	31.66	25.25	-	0.80	3.42	1.09
**SrSeMgS**	4.30	P-3m1	31.39	1.74	6.60	33.13	29.65	-	0.89	3.78	0.87
**SrSeZnO**	3.65	P-3m1	30.40	3.65	13.03	34.05	26.76	-	0.79	3.09	1.01
**SrSeZnS**†	4.14	P-3m1	3.84	4.90	8.62	8.74	-1.06	-	-0.12	1.72	1.29
**SrTeBaO**	4.45	P-3m1	31.51	9.17	28.41	40.68	22.33	-	0.55	3.38	1.60
**SrTeMgO**	3.54	P-3m1	45.97	12.32	38.87	58.29	33.64	-	0.58	3.86	1.77
**SrTeZnS**	4.88	P-3m1	20.50	2.06	7.47	22.55	18.44	-	0.82	2.99	0.90
**ZnOBaSe**	4.59	P-3m1	23.66	2.83	10.10	26.49	20.83	-	0.79	3.13	1.02
**ZnOBeS**	3.45	P-3m1	93.83	42.18	116.40	136.01	51.65	-	0.38	8.31	4.63
**ZnOMgTe**	3.43	P-3m1	45.39	16.29	47.95	61.68	29.10	-	0.47	4.03	2.07
**ZnSBaO**	4.08	P-3m1	57.18	11.50	38.30	68.68	45.68	-	0.67	4.87	1.99
**ZnSBeSe**	3.77	P-3m1	76.52	33.16	92.55	109.68	43.35	-	0.40	6.61	3.64
**ZnSCdO**	3.79	P-3m1	78.35	24.12	73.78	102.47	54.22	-	0.53	5.83	2.83
**ZnSMgSe**	4.03	P-3m1	46.62	19.19	54.39	65.81	27.42	-	0.42	5.27	2.85
**ZnSSrO**	3.96	P-3m1	79.60	18.28	59.46	97.88	61.33	-	0.63	6.32	2.73
**ZnSeCdS**	4.14	P-3m1	53.00	19.56	57.15	72.56	33.44	-	0.46	4.73	2.46
**ZnSeMgS**	4.07	P-3m1	66.97	20.05	61.72	87.02	46.92	-	0.54	6.11	2.93
**ZnSeSrS**	4.32	P-3m1	40.38	2.15	8.17	42.53	38.23	-	0.90	3.96	0.89
**ZnTeBaS**†	4.62	P-3m1	3.10	7.75	8.86	10.86	-4.65	-	-0.43	1.83	1.54
**ZnTeBeS**	3.51	P-3m1	32.19	12.85	36.73	45.03	19.34	-	0.43	3.52	1.88
**ZnTeMgS**	4.20	P-3m1	62.05	10.24	35.17	72.30	51.81	-	0.72	5.16	1.94
**ZnTeSrO**	4.16	P-3m1	47.84	15.23	46.20	63.06	32.61	-	0.52	4.38	2.15

TABLE S4: Calculated mechanical and elastic properties of 2D materials at zero pressure and temperature of 300 K. The in-plane stiffness K (i.e., the 2D equivalent of bulk modulus), the shear modulus G, the 2D Young's modulus Y^{2D}, and the elastic constant tensor C_{ij} are in N/m; ν is the Poisson ratio; and V_l and V_t are the longitudinal and shear sound velocity in km/s, respectively. The superscript (†) indicated auxetic materials, i.e., negative Poisson ratio, which implies anti-rubber behavior.Note for isotropic 2D materials, $C_{66} = (C_{ii} - C_{ij})/2$, where $i = 1, 2$ and has been omitted in the table. Superscript ♣ denotes an unstable structure.

Material	K	G	Y^{2D}	$C_{11}(C_{22})$	C_{12}	C_{66}	ν	V_l	V_t
**2D Materials**									
**Graphene**	215.32	99.59	272.39	314.92	115.73	-	0.37	20.40	11.47
**Borophene**	189.15	146.435	330.065	377.88(292.60)	41.29	103.93	0.13	18.46	12.20
**Silicene**	37.42	21.64	54.84	59.05	15.78	-	0.27	9.01	5.45
**Stanene**	10.52	6.01	15.31	16.54	4.51	-	0.27	2.78	1.68
**Germanene**	18.00	12.56	29.59	30.56	5.43	-	0.18	4.23	2.71
**Phosphorene**♣	-	-	-	-	-	-	-	-	-
**BN**	170.26	111.14	268.98	281.40	59.11	-	0.21	19.30	12.13
**BP**	87.10	49.44	126.16	136.54	37.65	-	0.28	13.23	7.96
**BAs**	60.61	40.13	96.57	100.74	20.48	-	0.20	8.37	5.28
**AlN**	83.63	33.91	96.51	117.54	49.73	-	0.42	12.07	6.48
**AlP**	17.15	16.22	33.34	33.37	0.93	-	0.03	6.80	4.74
**AlAs**†	12.70	14.25	26.86	26.94	-1.55	-	-0.06	4.79	3.48
**GaN**	81.67	32.44	92.86	114.10	49.23	-	0.43	8.66	4.62
**GaP**†	13.47	16.52	29.68	29.99	-3.06	-	-0.10	4.90	3.64
**GaAs**†	12.09	15.05	26.82	27.14	-2.95	-	-0.11	4.05	3.02
**InN**	41.62	18.73	51.67	60.35	22.89	-	0.38	5.67	3.16
**InP**†	9.83	12.30	21.85	22.12	-2.47	-	-0.11	3.81	2.84
**InAs**†	6.35	9.38	15.14	15.73	-3.04	-	-0.19	2.91	2.25
**2H-MoS$_2$**	84.91	53.96	131.98	138.88	30.95	-	0.22	6.71	4.18
**2H-MoSe$_2$**	69.34	45.69	110.17	115.03	23.65	-	0.21	5.05	3.18
**2H-MoTe$_2$**	54.38	35.42	85.79	89.79	18.96	-	0.21	4.05	2.54
**2H-WS$_2$**	90.60	61.21	146.12	151.82	29.36	-	0.19	5.65	3.58

Continued on next page

TABLE S4 – Continued from previous page

Material	K	G	Y^{2D}	$C_{11}(C_{22})$	C_{12}	C_{66}	ν	V_l	V_t
**2H-WSe_2**	72.89	51.80	121.12	124.69	21.09	-	0.17	4.54	2.92
**2H-WTe_2**	49.96	40.51	89.48	90.46	9.45	-	0.10	3.64	2.44
**2H-CrS_2**	78.99	47.63	118.86	126.62	31.25	-	0.25	7.18	4.44
**2H-$CrSe_2$**	65.97	36.59	94.14	102.56	29.38	-	0.29	5.07	3.03
**2H-$CrTe_2$**	129.57	19.95	69.16	149.52	109.62	-	0.73	5.56	2.03
**2H-MoO_2**	151.12	72.48	195.94	223.60	78.64	-	0.35	8.57	4.88
**2H-WO_2**	166.80	84.32	224.03	251.12	82.47	-	0.33	7.02	4.07
**2H-CrO_2**	95.81	50.87	132.91	146.68	44.94	-	0.31	8.31	4.89
**2H-HfS_2**	61.63	28.56	78.06	90.19	33.08	-	0.37	4.88	2.75
**2H-$HfSe_2$**	54.82	23.65	66.09	78.47	31.17	-	0.40	4.01	2.20
**2H-$HfTe_2$**	35.07	10.39	32.05	45.45	24.68	-	0.54	2.85	1.36
**2H-TiS_2**	59.20	29.07	77.98	88.27	30.14	-	0.34	6.70	3.85
**2H-$TiSe_2$**	49.10	21.52	59.84	70.61	27.58	-	0.39	4.61	2.55
**2H-$TiTe_2$**	28.62	3.13	11.30	31.75	25.49	-	0.80	2.72	0.85
**2H-ZrS_2**	56.61	24.53	68.45	81.14	32.08	-	0.40	5.84	3.21
**2H-$ZrSe_2$**	47.76	19.15	54.68	66.91	28.61	-	0.43	4.34	2.32
**2H-$ZrTe_2$**	30.31	6.26	20.74	36.56	24.05	-	0.66	2.86	1.18
**2H-TiO_2**	112.74	62.87	161.45	175.61	49.88	-	0.28	9.73	5.82
**2H-HfO_2**	94.85	56.76	142.04	151.61	38.10	-	0.25	6.01	3.68
**2H-ZrO_2**†	35.46	41.11	76.16	76.57	-5.66	-	-0.07	5.65	4.14
**2H-GeO_2**	119.98	57.24	155.00	177.22	62.75	-	0.35	8.44	4.80
**2H-GeS_2**♣	-	-	-	-	-	-	-	-	-
**2H-$GeSe_2$**♣	-	-	-	-	-	-	-	-	-
**2H-$GeTe_2$**	7.49	1.50	5.00	8.99	5.98	-	0.67	1.69	0.69
**2H-SiO_2**♣	-	-	-	-	-	-	-	-	-
**2H-SiS_2**	83.22	51.61	127.42	134.83	31.61	-	0.23	8.88	5.49
**2H-$SiSe_2$**	87.43	15.95	53.96	103.38	71.48	-	0.69	5.79	2.27
**2H-$SiTe_2$**♣	-	-	-	-	-	-	-	-	-
**2H-SnO_2**♣	-	-	-	-	-	-	-	-	-
**2H-SnS_2**♣	-	-	-	-	-	-	-	-	-
**2H-$SnSe_2$**	19.25	5.04	15.99	24.30	14.21	-	0.58	2.54	1.16
**2H-$SnTe_2$**	4.28	1.79	5.05	6.07	2.49	-	0.41	1.09	0.59
**2H-PbO_2**♣	-	-	-	-	-	-	-	-	-
**2H-PbS_2**♣	-	-	-	-	-	-	-	-	-
**2H-$PbSe_2$**♣	-	-	-	-	-	-	-	-	-
**2H-$PbTe_2$**♣	-	-	-	-	-	-	-	-	-
**GeS**	11.32/37.60	3.04	8.25/15.70	67.70(35.59)	43.02	27.53	0.64/1.21	3.42	1.17
**GeSe**	19.65/1107.14	7.46	18.54/35.68	77.00(40.02)	40.67	33.94	0.53/1.02	2.93/19.30	1.57
**GeTe**	16.63/202.79	4.44	12.40/20.77	61.01(36.41)	38.27	33.83	0.63/1.05	2.38/7.60	1.11
**SiS**	15.67/1016.27	11.92	21.10/62.53	91.54(30.88)	29.93	16.53	0.33/0.97	4.26/28.15	2.98
**SiSe**	23.58/382.10	5.23	15.46/23.71	77.24(50.36)	51.92	33.42	0.67/1.03	3.57/13.24	1.54
**SiTe**	39.89/118.45	9.38	28.45/37.26	81.39(62.14)	52.37	39.08	0.64/0.84	5.35	1.79
**SnS**	17.04/110.30	7.46	18.02/32.49	54.47(30.12)	25.67	29.87	0.47/0.85	2.80/6.35	1.58
**SnSe**	17.04/110.30	7.46	18.02/32.59	54.47(30.12)	25.67	29.87	0.47/0.85	2.80/6.35	1.58
**SnTe**	4.16/23.56	5.40	7.05/23.29	25.25(7.64)	3.86	27.02	0.15/0.51	2.13	1.17
**PbS**♣	-	-	-	-	-	-	-	-	-
**PbS**♣	-	-	-	-	-	-	-	-	-
**PbTe**♣	-	-	-	-	-	-	-	-	-
**1T-MoS_2**	70.85	43.79	108.26	114.64	27.05	-	0.24	6.05	3.74
**1T-$MoSe_2$**	61.98	35.79	90.75	97.76	26.19	-	0.27	4.58	2.77
**1T-$MoTe_2$**	47.53	35.13	80.80	82.66	12.40	-	0.15	3.81	2.49
**1T-WS_2**	72.90	41.90	106.42	114.79	31.00	-	0.27	4.92	2.97
**1T-WSe_2**	66.70	38.73	98.01	105.43	27.96	-	0.27	4.12	2.49
**1T-WTe_2**	19.88	5.29	16.72	25.17	14.58	-	0.58	2.06	0.94
**1T-CrS_2**	135.73	64.08	174.12	199.81	71.65	-	0.36	9.03	5.11
**1T-$CrSe_2$**♣	-	-	-	-	-	-	-	-	-
**1T-$CrTe_2$**	62.34	34.54	88.90	96.88	27.80	-	0.29	4.38	2.62
**1T-MoO_2**	100.22	53.21	139.03	153.43	47.01	-	0.31	7.32	4.31
**1T-WO_2**	112.98	57.49	152.40	170.46	55.49	-	0.33	5.93	3.44

Continued on next page

TABLE S4 – Continued from previous page

Material	K	G	Y^{2D}	$C_{11}(C_{22})$	C_{12}	C_{66}	ν	V_l	V_t
**1T-CrO_2♣**	-	-	-	-	-	-	-	-	-
**1T-HfS_2**	49.16	32.85	78.77	82.01	16.31	-	0.20	4.80	3.03
**1T-$HfSe_2$**	43.42	27.24	66.96	70.67	16.18	-	0.23	3.90	2.42
**1T-$HfTe_2$**	30.02	17.49	44.20	47.51	12.53	-	0.26	2.94	1.79
**1T-TiS_2**	40.81	31.70	71.37	72.51	9.12	-	0.13	6.19	4.09
**1T-$TiSe_2$**	39.74	24.75	61.00	64.49	15.00	-	0.23	4.45	2.76
**1T-$TiTe_2$**	22.60	9.31	26.37	31.91	13.29	-	0.42	2.72	1.47
**1T-ZrS_2**	46.13	29.23	71.56	75.36	16.19	-	0.22	5.80	3.61
**1T-$ZrSe_2$**	38.32	24.14	59.24	62.46	14.18	-	0.23	4.29	2.67
**1T-$ZrTe_2$**	25.87	12.96	34.55	38.83	12.90	-	0.33	2.98	1.72
**1T-TiO_2**	83.19	51.56	127.32	134.75	31.63	-	0.23	8.88	5.49
**1T-HfO_2**	101.18	63.83	156.55	165.01	37.35	-	0.23	6.54	4.07
**1T-ZrO_2**	84.99	57.00	136.48	141.99	27.98	-	0.20	8.02	5.08
**1T-GeO_2**	99.11	55.73	142.69	154.84	43.38	-	0.28	8.15	4.89
**1T-GeS_2**	57.45	32.82	83.55	90.27	24.63	-	0.27	6.34	3.82
**1T-$GeSe_2$**	45.05	13.48	41.51	58.53	31.57	-	0.54	4.14	1.99
**1T-$GeTe_2$♣**	-	-	-	-	-	-	-	-	-
**1T-SiO_2**	104.38	60.79	153.66	165.17	43.60	-	0.26	10.54	6.39
**1T-SiS_2**	70.79	44.05	108.61	114.84	26.74	-	0.23	8.33	5.16
**1T-$SiSe_2$**	59.70	32.56	84.28	92.26	27.14	-	0.29	5.59	3.32
**1T-$SiTe_2$**	44.34	10.32	33.48	54.66	34.02	-	0.62	3.76	1.63
**1T-SnO_2**	85.08	45.55	118.67	130.63	39.52	-	0.30	6.83	4.04
**1T-SnS_2**	45.14	26.13	66.20	71.27	19.01	-	0.27	5.22	3.16
**1T-$SnSe_2$**	39.56	21.65	55.97	61.22	17.19	-	0.29	4.10	2.44
**1T-$SnTe_2$**	17.90	5.29	16.34	23.19	12.61	-	0.54	2.31	1.11
**1T-PbO_2**	59.56	30.68	81.00	90.24	28.87	-	0.32	4.76	2.78
**1T-PbS_2**	32.26	15.91	42.62	48.17	16.34	-	0.34	3.66	2.11
**1T-$PbSe_2$**	3.43	1.14	3.41	4.57	2.30	-	0.50	1.01	0.50
**1T-$PbTe_2$♣**	-	-	-	-	-	-	-	-	-
**BaO**	7.25	5.91	13.02	13.16	1.34	-	0.10	2.85	1.91
**BaS**	10.34	1.69	5.82	12.03	8.64	-	0.72	3.12	1.17
**BaSe♣**	-	-	-	-	-	-	-	-	-
**BaTe**	5.27	1.20	3.91	6.47	4.07	-	0.63	2.03	0.87
**BeO**	68.48	42.08	104.26	110.56	26.40	-	0.24	13.27	8.19
**BeS**	27.24	23.73	50.73	50.97	3.52	-	0.07	8.75	5.97
**BeSe†**	16.51	19.33	35.62	35.84	-2.81	-	-0.08	5.33	3.91
**BeTe**	13.60	13.18	26.78	26.78	0.41	-	0.02	4.06	2.85
**CdO**	33.73	7.00	23.20	40.73	26.73	-	0.66	4.72	1.96
**CdS**	8.37	7.62	15.95	15.99	0.76	-	0.05	3.21	2.22
**CdSe**	8.00	7.01	14.94	15.00	0.99	-	0.07	2.84	1.94
**CdTe†**	6.75	7.07	13.81	13.82	-0.31	-	-0.02	2.59	1.85
**MgO**	60.17	16.31	51.33	76.48	43.86	-	0.57	10.36	4.78
**MgS**	14.78	8.36	21.36	23.14	6.42	-	0.28	5.97	3.59
**MgSe**	7.86	6.29	13.98	14.15	1.57	-	0.11	3.63	2.42
**MgTe**	8.09	6.28	14.15	14.37	1.81	-	0.13	3.27	2.16
**SrO**	18.65	5.87	17.85	24.52	12.79	-	0.52	4.47	2.18
**SrS†**	0.82	1.50	2.11	2.32	-0.68	-	-0.30	1.53	1.23
**SrSe♣**	-	-	-	-	-	-	-	-	-
**SrTe♣**	-	-	-	-	-	-	-	-	-
**ZnO**	49.43	14.46	44.74	63.88	34.97	-	0.55	6.64	3.16
**ZnS**	14.01	12.83	26.79	26.84	1.18	-	0.04	4.64	3.21
**ZnSe†**	12.12	12.71	24.81	24.83	-0.59	-	-0.02	3.87	2.77
**ZnTe**	12.00	7.95	19.12	19.95	4.05	-	0.20	3.24	2.04
**$CoSiS_3$**	139.09	99.38	231.85	238.47	39.71	-	0.17	6.08	3.92
**$CoSiSe_3$**	118.94	84.03	196.96	202.97	34.91	-	0.17	4.48	2.88
**$CoSiTe_3$**	89.33	69.08	155.82	158.41	20.15	-	0.13	3.57	2.36
**$CrSiS_3$**	49.36	30.89	75.99	80.24	18.47	-	0.23	3.67	2.27
**$CrSiSe_3$ ♣**	-	-	-	-	-	-	-	-	-
**$CrSiTe_3$**	221.44	71.67	216.59	293.11	149.76	-	0.51	4.91	2.43
**$CuSiS_3$♣**	-	-	-	-	-	-	-	-	-

Continued on next page

TABLE S4 – Continued from previous page

Material	K	G	Y^{2D}	$C_{11}(C_{22})$	C_{12}	C_{66}	ν	V_l	V_t
**$CuSiSe_3$**	63.48	17.41	54.66	80.89	46.07	-	0.57	2.85	1.32
**$CuSiTe_3$**	115.53	48.98	137.58	164.51	66.56	-	0.40	3.70	2.02
**$FeSiS_3$**	181.68	87.32	235.90	269.00	94.36	-	0.35	6.77	3.86
**$FeSiSe_3$**	153.32	45.86	141.22	199.19	107.46	-	0.54	4.51	2.16
**$FeSiTe_3$**	120.09	32.44	102.16	152.53	87.65	-	0.57	3.57	1.65
**$MnSiS_3$**	180.25	15.58	57.37	195.83	164.67	-	0.84	5.76	1.63
**$MnSiSe_3$**	103.35	32.66	99.27	136.01	70.70	-	0.52	3.72	1.82
**$MnSiTe_3$**	227.89	41.79	141.24	269.67	186.10	-	0.69	4.74	1.86
**$NiSiS_3$**	89.58	79.87	168.89	169.44	9.71	-	0.06	5.18	3.56
**$NiSiSe_3$**	107.14	54.93	145.25	162.07	52.21	-	0.32	4.04	2.35
**$NiSiTe_3$**	111.79	41.53	121.13	153.32	70.26	-	0.46	3.55	1.85
**$VSiS_3$**♣	-	-	-	-	-	-	-	-	-
**$VSiSe_3$**	124.78	75.57	188.26	200.35	49.21	-	0.25	4.54	2.79
**$VSiTe_3$**	163.85	15.40	56.31	179.25	148.45	-	0.83	3.86	1.13
**$CoGeS_3$**†	81.50	94.67	175.19	176.18	-13.17	-	-0.07	4.78	3.51
**$CoGeSe_3$**†	77.59	94.34	170.30	171.93	-16.75	-	-0.10	3.93	2.91
**$CoGeTe_3$**	95.68	49.18	129.94	144.87	46.50	-	0.32	3.31	1.93
**$CrGeS_3$** ♣	-	-	-	-	-	-	-	-	-
**$CrGeSe_3$**	60.54	58.82	119.34	119.36	1.71	-	0.01	3.32	2.33
**$CrGeTe_3$**♣	-	-	-	-	-	-	-	-	-
**$CuGeS_3$**♣	-	-	-	-	-	-	-	-	-
**$CuGeSe_3$**	73.27	32.40	89.87	105.67	40.86	-	0.39	3.10	1.71
**$CuGeTe_3$**	117.91	56.70	153.15	174.61	61.21	-	0.35	3.67	2.09
**$FeGeS_3$**	235.52	57.31	184.38	292.83	178.20	-	0.61	6.47	2.86
**$FeGeSe_3$**	109.34	58.78	152.92	168.12	50.56	-	0.30	3.98	2.35
**$FeGeTe_3$**	62.02	18.20	56.29	80.23	43.86	-	0.55	2.50	1.19
**$MnGeS_3$**	141.56	65.89	179.85	207.45	75.67	-	0.36	5.44	3.07
**$MnGeSe_3$**	107.03	52.53	140.94	159.56	54.51	-	0.34	3.97	2.28
**$MnGeTe_3$**	104.84	31.96	97.96	136.79	72.88	-	0.53	3.29	1.59
**$NiGeS_3$**	156.92	17.70	63.62	174.61	139.22	-	0.80	4.84	1.54
**$NiGeSe_3$**	89.32	42.62	115.40	131.93	46.70	-	0.35	3.47	1.97
**$NiGeTe_3$**	98.84	28.37	88.18	127.21	70.47	-	0.55	3.15	1.49
**$VGeS_3$** ♣	-	-	-	-	-	-	-	-	-
**$VGeSe_3$**†	33.52	40.07	73.00	73.58	-6.55	-	-0.09	2.62	1.93
**$VGeTe_3$**	114.84	11.18	40.75	126.02	103.66	-	0.82	3.12	0.93
				**2D Heterostructures**					
				**Heterostructure with two 1T monolayer**					
**CrO_2GeS_2**	108.02	47.82	132.59	155.84	60.20	-	0.39	6.33	3.51
**CrS_2GeO_2**	161.12	66.28	187.85	227.40	94.84	-	0.42	6.86	3.70
**CrS_2GeSe_2**	41.49	9.79	31.69	51.28	31.70	-	0.62	2.96	1.29
**CrS_2GeTe_2**	47.53	12.56	39.74	60.09	34.97	-	0.58	2.84	1.30
**$CrSe_2GeO_2$**	156.77	83.75	218.36	240.52	73.01	-	0.30	6.04	3.56
**$CrSe_2GeS_2$**	70.92	34.79	93.35	105.70	36.13	-	0.34	4.20	2.41
**$CrTe_2GeO_2$**	142.33	62.96	174.60	205.29	79.37	-	0.39	5.05	2.80
**$CrTe_2GeS_2$**	45.61	36.65	81.28	82.26	8.95	-	0.11	3.42	2.28
**$CrTe_2GeSe_2$**	85.03	22.78	71.86	107.81	62.25	-	0.58	3.63	1.67
**CrO_2HfS_2**	121.70	64.62	168.84	186.32	57.08	-	0.31	5.60	3.30
**CrS_2HfO_2**	136.60	53.33	153.43	189.93	83.27	-	0.44	5.52	2.92
**CrS_2HfSe_2**	69.49	44.38	108.33	113.87	25.12	-	0.22	3.94	2.46
**$CrSe_2HfO_2$**	160.16	129.46	286.36	289.62	30.70	-	0.11	6.09	4.07
**$CrSe_2HfS_2$**	59.33	40.01	95.58	99.34	19.32	-	0.19	3.72	2.36
**$CrTe_2HfO_2$**	166.53	95.89	243.41	262.43	70.64	-	0.27	5.35	3.23
**$CrTe_2HfS_2$**	67.10	56.42	122.60	123.53	10.68	-	0.09	3.81	2.58
**$CrTe_2HfSe_2$**	73.41	32.76	90.60	106.17	40.65	-	0.38	3.33	1.85
**CrO_2MnSe_2**	21.89	12.22	31.37	34.11	9.67	-	0.28	2.31	1.38
**CrS_2MnO_2**	59.71	12.18	40.46	71.89	47.53	-	0.66	3.97	1.64
**CrS_2MnSe_2**	130.71	22.31	76.22	153.01	108.40	-	0.71	4.84	1.85
**CrS_2MnTe_2**	58.80	32.54	83.80	91.35	26.26	-	0.29	3.44	2.05
**$CrSe_2MnS_2$**	127.32	70.14	180.91	197.46	57.17	-	0.29	5.57	3.32
**$CrSe_2MnTe_2$**	118.85	72.72	180.45	191.56	46.13	-	0.24	4.60	2.84

Continued on next page

TABLE S4 – Continued from previous page

Material	K	G	Y^{2D}	$C_{11}(C_{22})$	C_{12}	C_{66}	ν	V_l	V_t
**$CrTe_2MnS_2$**	67.73	37.17	96.00	104.90	30.56	-	0.29	3.75	2.23
**$CrTe_2MnSe_2$**†	65.46	77.16	141.66	142.62	-11.70	-	-0.08	4.03	2.97
**CrO_2MoS_2**	74.42	23.91	72.38	98.33	50.51	-	0.51	4.36	2.15
**CrS_2MoO_2**	173.45	49.42	153.85	222.87	124.03	-	0.56	6.42	3.02
**CrS_2MoSe_2**	86.49	42.24	113.51	128.72	44.25	-	0.34	4.25	2.43
**CrS_2MoTe_2**	83.36	41.77	111.31	125.14	41.59	-	0.33	3.89	2.25
**$CrSe_2MoO_2$**	139.87	99.69	232.83	239.56	40.18	-	0.17	5.80	3.74
**$CrSe_2MoS_2$**	133.16	79.11	198.50	212.27	54.05	-	0.25	5.48	3.34
**$CrSe_2MoTe_2$**	65.21	45.25	106.85	110.46	19.95	-	0.18	3.39	2.17
**$CrTe_2MoS_2$**	128.60	65.99	174.45	194.59	62.61	-	0.32	4.85	2.82
**$CrTe_2MoSe_2$**	134.66	68.88	182.28	203.53	65.78	-	0.32	4.58	2.66
**CrS_2PbO_2**	65.41	41.87	102.12	107.28	23.53	-	0.22	4.09	2.55
**CrS_2PbSe_2**	40.41	26.03	63.33	66.44	14.38	-	0.22	2.60	1.63
**CrS_2PbTe_2**	68.03	77.54	144.94	145.56	-9.51	-	-0.07	3.64	2.66
**GeO_2CrS_2**	160.87	113.34	265.98	274.21	47.53	-	0.17	7.54	4.84
**GeO_2CrSe_2**	158.19	109.30	258.55	267.49	48.89	-	0.18	6.37	4.07
**GeO_2CrTe_2**	146.84	59.85	170.07	206.69	86.99	-	0.42	5.07	2.73
**GeS_2MoO_2**†	21.87	42.09	57.57	63.96	-20.22	-	-0.32	3.69	2.99
**GeO_2MoS_2**	162.45	94.59	239.13	257.05	67.86	-	0.26	6.79	4.12
**GeO_2MoSe_2**	143.84	78.68	203.44	222.52	65.16	-	0.29	5.53	3.29
**GeS_2HfO_2**	171.29	97.79	249.01	269.08	73.50	-	0.27	6.64	4.00
**GeO_2MnS_2**	182.00	159.23	339.71	341.23	22.77	-	0.07	8.36	5.71
**$GeTe_2MoS_2$**†	17.78	34.96	47.15	52.75	-17.18	-	-0.33	2.56	2.08
**$GeTe_2PbSe_2$**	6.22	2.24	6.59	8.46	3.98	-	0.47	1.00	0.52
**GeO_2SiS_2**	170.94	83.36	224.14	254.30	87.58	-	0.34	8.04	4.60
**$GeTe_2SiSe_2$**†	10.70	12.34	22.92	23.04	-1.65	-	-0.07	1.71	1.25
**GeS_2SnO_2**	142.65	76.34	198.92	218.99	66.30	-	0.30	6.60	3.90
**$GeTe_2SnSe_2$**	48.81	19.88	56.50	68.69	28.93	-	0.42	2.96	1.59
**HfO_2MoSe_2**	157.38	106.42	253.96	263.81	50.96	-	0.19	5.56	3.53
**$HfSe_2MoS_2$**	74.82	43.01	109.24	117.83	31.81	-	0.27	3.85	2.32
**HfS_2PbSe_2**†	15.72	22.02	36.69	37.74	-6.31	-	-0.17	2.14	1.63
**HfO_2SiS_2**	183.77	110.74	276.40	294.51	73.03	-	0.25	7.31	4.48
**HfS_2SnO_2**	109.51	63.97	161.52	173.47	45.54	-	0.26	5.15	3.13
**HfS_2WTe_2**	124.13	79.39	193.68	203.51	44.74	-	0.22	4.41	2.76
**MnO_2CrS_2**	74.96	21.38	66.55	96.34	53.57	-	0.56	4.57	2.15
**MnO_2GeS_2**	132.51	53.34	152.12	185.84	79.17	-	0.43	6.58	3.52
**MnS_2HfO_2**	144.99	77.61	202.21	222.61	67.38	-	0.30	6.00	3.54
**MnO_2MoS_2**	91.87	50.37	130.13	142.23	41.50	-	0.29	5.19	3.09
**MnS_2MoO_2**	207.79	94.30	259.46	302.09	113.48	-	0.38	7.43	4.15
**MnO_2PbS_2**	15.86	8.53	22.19	24.39	7.34	-	0.30	1.99	1.18
**$MnTe_2PbSe_2$**	14.40	12.43	26.68	26.82	1.97	-	0.07	1.77	1.20
**$MnTe_2SnO_2$**	147.65	76.83	202.14	224.48	70.82	-	0.32	5.25	3.07
**MoS_2GeO_2**	173.47	116.16	278.29	289.63	57.31	-	0.20	7.21	4.56
**$MoSe_2GeO_2$**	143.70	70.95	190.00	214.66	72.75	-	0.34	5.43	3.12
**MoS_2HfSe_2**	77.36	48.03	118.53	125.39	29.33	-	0.23	3.97	2.46
**$MoTe_2HfSe_2$**	82.47	17.69	58.25	100.16	64.79	-	0.65	3.14	1.32
**MoS_2SiO_2**	175.88	92.54	242.54	268.42	83.34	-	0.31	7.33	4.31
**$MoTe_2WSe_2$**	123.86	25.09	83.45	148.95	98.77	-	0.66	3.57	1.47
**PbO_2HfS_2**	113.29	57.60	152.73	170.88	55.69	-	0.33	4.75	2.76
**PbO_2MnS_2**	70.06	59.07	128.19	129.13	10.99	-	0.09	4.52	3.06
**PbO_2MoS_2**	130.47	52.75	150.25	183.22	77.72	-	0.42	5.10	2.74
**$PbTe_2WSe_2$**	117.88	36.70	111.95	154.58	81.18	-	0.53	3.26	1.59
**SiO_2GeTe_2**	47.17	8.35	28.38	55.52	38.82	-	0.70	2.56	0.99
**$SiSe_2HfO_2$**	153.55	75.23	201.96	228.78	78.33	-	0.34	5.78	3.32
**$SiTe_2HfSe_2$**	80.43	6.80	25.10	87.24	73.63	-	0.84	3.21	0.90
**SiO_2MnS_2**	131.80	76.00	192.82	207.80	55.80	-	0.27	6.94	4.20
**SiO_2MoS_2**	171.86	92.56	240.63	264.42	79.30	-	0.30	7.28	4.31
**SiO_2PbS_2**	97.91	58.75	146.87	156.66	39.16	-	0.25	4.49	2.75
**$SiTe_2SnSe_2$**	83.76	10.80	38.26	94.55	72.96	-	0.77	3.57	1.21
**SiO_2WS_2**	185.64	101.09	261.80	286.73	84.54	-	0.29	6.43	3.82

Continued on next page

TABLE S4 – Continued from previous page

Material	K	G	Y^{2D}	$C_{11}(C_{22})$	C_{12}	C_{66}	ν	V_l	V_t
SnO_2GeS_2	140.52	72.06	190.54	212.58	68.45	-	0.32	6.50	3.79
$SnSe_2GeTe_2$	44.50	16.95	49.09	61.45	27.55	-	0.45	2.80	1.47
SnO_2HfS_2	112.82	61.52	159.24	174.33	51.30	-	0.29	5.16	3.07
$SnTe_2HfSe_2$	57.26	12.63	41.40	69.89	44.62	-	0.64	2.79	1.19
SnO_2MnS_2	133.69	110.92	242.49	244.61	22.77	-	0.09	6.93	4.67
$SnTe_2PbSe_2$	10.55	0.55	2.07	11.10	10.01	-	0.90	1.26	0.28
$SnTe_2SiS_2$	158.00	81.97	215.89	239.97	76.02	-	0.32	5.31	3.10
SnO_2TiTe_2	90.54	9.16	33.28	99.70	81.38	-	0.82	3.58	1.09
$SnTe_2WO_2$	78.20	56.08	130.63	134.28	22.13	-	0.16	3.36	2.17
$SnTe_2WSe_2$	139.49	34.83	111.48	174.31	104.66	-	0.60	3.64	1.63
TiO_2GeS_2	102.64	26.77	84.93	129.41	75.87	-	0.59	5.64	2.56
$TiTe_2GeSe_2$	70.23	6.08	22.39	76.31	64.14	-	0.84	3.15	0.89
TiO_2HfS_2	71.33	12.11	41.42	83.45	59.22	-	0.71	3.79	1.44
TiO_2MnS_2	163.40	114.90	269.85	278.30	48.50	-	0.17	8.14	5.23
TiO_2MoS_2	147.08	73.56	196.15	220.64	73.52	-	0.33	6.67	3.85
TiO_2MoSe_2	124.51	85.38	202.60	209.89	39.12	-	0.19	5.61	3.58
TiO_2PbSe_2	66.58	47.89	111.41	114.46	18.69	-	0.16	3.51	2.27
$TiTe_2PbSe_2$	40.33	15.88	45.57	56.20	24.45	-	0.43	2.59	1.38
TiO_2SnS_2	87.47	16.22	54.73	103.69	71.25	-	0.69	4.74	1.88
$TiTe_2SnSe_2$	64.85	33.88	89.01	98.73	30.97	-	0.31	3.58	2.10
TiO_2WS_2	152.73	70.37	192.70	223.10	82.36	-	0.37	5.75	3.23
WSe_2GeO_2	153.85	84.57	218.30	238.43	69.28	-	0.29	5.15	3.06
WO_2HfS_2	94.19	36.98	106.22	131.17	57.21	-	0.44	4.12	2.19
WTe_2HfSe_2	52.43	32.65	80.49	85.08	19.77	-	0.23	2.73	1.69
WO_2MnS_2	226.75	69.68	213.21	296.43	157.06	-	0.53	6.30	3.05
WO_2MoS_2	185.63	94.45	250.40	280.09	91.18	-	0.33	5.90	3.42
WSe_2MoS_2	141.80	84.23	211.37	226.04	57.57	-	0.25	4.92	3.00
WS_2PbO_2	122.64	79.25	192.57	201.89	43.39	-	0.21	4.85	3.04
WSe_2PbS_2	34.60	21.50	53.04	56.10	13.09	-	0.23	2.47	1.53
WTe_2PbSe_2	18.49	1.82	6.63	20.30	16.67	-	0.82	1.38	0.41
WO_2SiS_2	154.96	84.57	218.84	239.53	70.39	-	0.29	6.26	3.72
WTe_2SiS_2	117.44	24.07	79.91	141.51	93.36	-	0.66	3.97	1.64
WSe_2SnS_2	103.60	34.45	103.41	138.05	69.15	-	0.50	4.10	2.05
WTe_2SnSe_2	62.16	57.29	119.25	119.45	4.88	-	0.04	3.45	2.39
WS_2TiSe_2	84.75	16.82	56.14	101.58	67.93	-	0.67	3.58	1.46
WTe_2TiSe_2	101.02	60.11	150.74	161.13	40.91	-	0.25	3.97	2.42
**Heterostructure with two 2H monolayer**									
$CrO_2GeSe_2^{\dagger}$	13.22	24.88	34.54	38.10	-11.66	-	-0.31	2.20	1.78
$CrSe_2GeTe_2$	32.27	31.61	63.87	63.88	0.66	-	0.01	2.57	1.81
$CrSe_2HfTe_2$	87.74	37.20	104.49	124.94	50.55	-	0.40	3.34	1.82
CrO_2MnS_2	146.02	77.72	202.89	223.74	68.31	-	0.31	6.68	3.94
CrS_2MnSe_2	126.21	64.86	171.37	191.07	61.35	-	0.32	5.42	3.16
$CrSe_2MnS_2$	121.78	68.40	175.20	190.18	53.38	-	0.28	5.42	3.25
CrO_2MoS_2	188.54	102.73	265.99	291.27	85.81	-	0.29	7.24	4.30
$CrSe_2MoTe_2$	148.90	51.01	151.97	199.91	97.89	-	0.49	4.69	2.37
CrO_2PbS_2	89.96	27.31	83.80	117.27	62.65	-	0.53	3.71	1.79
$CrTe_2PbO_2^{\dagger}$	25.52	32.90	57.48	58.42	-7.38	-	-0.13	2.66	1.99
$CrO_2SiS_2^{\dagger}$	5.06	29.68	17.30	34.74	-24.62	-	-0.71	2.93	2.71
$CrTe_2SiS_2$	66.93	60.33	126.91	127.25	6.60	-	0.05	4.27	2.94
CrS_2SnTe_2	109.76	52.70	142.42	162.46	57.07	-	0.35	4.12	2.35
$CrTe_2SnSe_2^{\dagger}$	22.88	81.80	71.52	104.68	-58.92	-	-0.56	3.49	3.08
$CrSe_2TiO_2$	189.12	103.89	268.21	293.00	85.23	-	0.29	6.88	4.10
$CrTe_2TiS_2^{\dagger}$	5.77	41.05	20.23	46.82	-35.28	-	-0.75	2.57	2.40
CrO_2WS_2	193.98	118.22	293.82	312.20	75.76	-	0.24	6.46	3.97
GeO_2CrS_2	200.01	117.99	296.85	318.00	82.02	-	0.26	7.98	4.86
$GeSe_2CrTe_2$	57.33	29.66	78.18	86.98	27.67	-	0.32	3.34	1.95
GeS_2HfO_2	124.65	59.13	160.42	183.78	65.51	-	0.36	5.30	3.01
$GeTe_2HfSe_2^{\dagger}$	4.91	6.63	11.29	11.54	-1.72	-	-0.15	1.19	0.90
GeO_2MnS_2	168.46	89.85	234.38	258.31	78.61	-	0.30	7.19	4.24
GeS_2MnTe_2	97.72	29.80	91.34	127.52	67.92	-	0.53	4.19	2.03

Continued on next page

TABLE S4 – Continued from previous page

Material	K	G	Y^{2D}	$C_{11}(C_{22})$	C_{12}	C_{66}	ν	V_l	V_t
$GeTe_2MnSe_2^{\dagger}$	18.59	24.82	42.52	43.42	-6.23	-	-0.14	2.13	1.61
GeO_2MoS_2	212.22	125.87	316.03	338.09	86.35	-	0.26	7.72	4.71
$GeTe_2MoS_2$	112.69	56.06	149.75	168.76	56.63	-	0.34	4.27	2.46
GeO_2PbS_2	35.93	12.41	36.90	48.34	23.52	-	0.49	2.81	1.42
GeS_2PbSe_2	23.76	6.00	19.16	29.76	17.76	-	0.60	2.29	1.03
$GeTe_2PbSe_2$	30.47	9.56	29.11	40.03	20.91	-	0.52	2.19	1.07
$GeO_2SiS_2^{\dagger}$	40.57	153.43	128.34	194.00	-112.86	-	-0.58	6.81	6.06
$GeTe_2SiS_2$	52.00	26.55	70.30	78.55	25.46	-	0.32	3.23	1.88
$GeTe_2SnSe_2$	59.49	22.88	66.10	82.37	36.60	-	0.44	3.36	1.77
$GeTe_2TiO_2^{\dagger}$	45.34	56.99	101.00	102.33	-11.65	-	-0.11	3.48	2.59
GeO_2WS_2	218.07	133.00	330.45	351.07	85.07	-	0.24	6.84	4.21
$GeSe_2WO_2$	165.82	57.46	170.69	223.28	108.35	-	0.49	4.70	2.38
HfO_2CrS_2	113.92	70.27	173.85	184.20	43.65	-	0.24	5.26	3.25
HfO_2GeS_2	24.35	111.55	79.94	135.90	-87.20	-	-0.64	4.56	4.13
HfS_2GeSe_2	40.36	27.95	66.06	68.31	12.40	-	0.18	3.20	2.04
$HfTe_2GeSe_2$	11.21	2.84	9.08	14.06	8.37	-	0.60	1.33	0.60
HfS_2MnTe_2	48.81	31.13	76.03	79.94	17.68	-	0.22	3.04	1.90
$HfSe_2MoS_2$	107.92	22.69	75.00	130.61	85.23	-	0.65	3.96	1.65
$HfTe_2MoS_2$	118.55	50.41	141.47	168.95	68.14	-	0.40	4.01	2.19
HfO_2PbS_2	140.44	74.70	195.06	215.15	65.74	-	0.31	4.89	2.88
HfS_2PbTe_2	94.54	28.90	88.53	123.43	65.64	-	0.53	3.34	1.62
$HfTe_2PbS_2$	50.90	7.91	27.39	58.81	42.99	-	0.73	2.67	0.98
HfO_2SiS_2	220.85	122.75	315.60	343.60	98.09	-	0.29	7.64	4.56
$HfTe_2TiO_2$	90.00	20.69	67.30	110.69	69.30	-	0.63	3.30	1.43
$HfO_2WS_2^{\dagger}$	84.97	150.50	217.24	235.48	-65.53	-	-0.28	5.11	4.08
HfS_2WTe_2	114.98	58.95	155.88	173.93	56.03	-	0.32	4.06	2.36
MnO_2CrS_2	185.04	88.93	240.25	273.97	96.12	-	0.35	7.53	4.29
MnO_2GeS_2	107.62	43.96	124.84	151.58	63.67	-	0.42	5.42	2.92
$MnS_2HfO_2^{\dagger}$	19.40	72.99	61.30	92.39	-53.59	-	-0.58	3.73	3.32
MnS_2MoO_2	195.47	82.65	232.35	278.12	112.82	-	0.41	7.04	3.84
$MnTe_2PbSe_2$	32.53	10.09	30.82	42.63	22.44	-	0.53	2.27	1.11
$MnTe_2SnO_2$	131.35	91.32	215.47	222.67	40.03	-	0.18	5.10	3.27
MoS_2GeO_2	206.72	125.30	312.05	332.02	81.43	-	0.25	7.65	4.70
$MoSe_2GeO_2$	183.64	105.19	267.51	288.82	78.45	-	0.27	6.25	3.77
MoS_2HfSe_2	105.41	22.74	74.82	128.15	82.67	-	0.65	3.92	1.65
$MoSe_2SiTe_2$	107.68	41.61	120.06	149.29	66.07	-	0.44	4.07	2.15
$MoTe_2HfSe_2$	100.63	49.05	131.90	149.68	51.58	-	0.34	3.82	2.18
MoS_2SiO_2	185.66	85.04	233.30	270.70	100.62	-	0.37	7.35	4.12
$MoTe_2WSe_2$	128.95	88.98	210.59	217.93	39.98	-	0.18	4.34	2.78
PbO_2GeTe_2	36.11	15.40	43.18	51.50	20.71	-	0.40	2.52	1.38
$PbO_2MoS_2^{\dagger}$	4.85	52.61	17.77	57.46	-47.75	-	-0.83	2.78	2.66
$PbTe_2WSe_2$	115.60	55.37	149.76	170.97	60.23	-	0.35	3.47	1.97
$SiSe_2MoS_2$	68.87	40.52	102.05	109.39	28.34	-	0.26	4.17	2.54
$SiTe_2WS_2$	38.55	37.61	76.15	76.17	0.94	-	0.01	2.82	1.98
SnO_2GeS_2	181.17	88.41	237.67	269.59	92.76	-	0.34	7.08	4.05
$SnO_2MnS_2^{\dagger}$	67.32	77.10	143.76	144.42	-9.78	-	-0.07	5.16	3.77
SnO_2PbS_2	42.08	15.28	44.85	57.36	26.79	-	0.47	2.98	1.54
SnO_2TiTe_2	149.76	65.24	181.78	215.00	84.52	-	0.39	5.05	2.78
$SnTe_2WSe_2$	68.87	61.20	129.62	130.08	7.67	-	0.06	3.28	2.25
TiO_2GeS_2	130.29	44.17	131.95	174.46	86.12	-	0.49	6.25	3.14
TiO_2MoS_2	207.30	123.11	308.96	330.42	84.19	-	0.25	8.06	4.92
$TiSe_2SiO_2$	129.38	64.78	172.66	194.16	64.16	-	0.33	5.58	3.23
$TiTe_2SnSe_2$	38.69	18.80	50.61	57.49	19.89	-	0.35	2.87	1.64
WSe_2GeO_2	182.33	113.98	280.54	296.31	68.35	-	0.23	5.70	3.54
WO_2MnS_2	196.63	96.79	259.44	293.42	99.84	-	0.34	6.19	3.56
WSe_2MoS_2	159.19	107.17	256.20	266.36	52.02	-	0.20	5.35	3.39
WTe_2PbSe_2	72.72	3.88	14.72	76.60	68.85	-	0.90	2.50	0.56
WTe_2TiSe_2	99.54	58.62	147.57	158.16	40.92	-	0.26	3.95	2.40
**Heterostructure with one 2H and one 1T monolayer**									
CrO_2/HfS_2	120.13/131.22	64.48/67.54	167.83/178.37	197.43(185.77)	59.52	44.93	0.30/0.32	5.71/5.92	3.37/3.45

Continued on next page

TABLE S4 – Continued from previous page

Material	K	G	Y^{2D}	$C_{11}(C_{22})$	C_{12}	C_{66}	ν	V_l	V_t
**$CrTe_2/MoS_2$**	143.06	58.13	165.35	201.19	84.92	-	0.42	4.90	2.64
**$CrS_2/MnSe_2$**	103.32	10.67	38.69	113.99	92.65	-	0.81	4.23	1.29
**CrS_2/PbO_2**	114.07	18.89	64.84	132.96	95.17	-	0.72	4.43	1.67
**$CrTe_2/SnS_2$**	106.89	28.30	89.50	135.19	78.59	-	0.58	4.28	1.96
**GeO_2/MnS_2**	151.56	76.98	204.20	228.54	74.58	-	0.33	6.92	4.01
**GeO_2/MoS_2**	177.18	111.52	273.76	288.69	65.66	-	0.23	7.26	4.51
**$GeO_2/MoSe_2$**	168.71	77.23	211.91	245.94	91.49	-	0.37	5.86	3.29
**GeO_2/PbS_2**	80.10	34.68	96.81	114.78	45.42	-	0.40	3.83	2.11
**GeS_2/MoO_2**	144.17	24.79	84.61	168.96	119.38	-	0.71	5.61	2.15
**GeS_2/SnO_2**	136.62	69.93	185.02	206.55	66.69	-	0.32	6.42	3.74
**$GeTe_2/WSe_2$**	61.07	51.55	111.82	112.62	9.52	-	0.08	3.21	2.17
**HfO_2/MoS_2**	188.05	117.53	289.30	305.58	70.53	-	0.23	6.59	4.09
**$HfO_2/MoSe_2$**	167.96	108.72	264.00	276.68	59.24	-	0.21	5.73	3.59
**HfS_2/MoO_2**	93.35	10.21	36.82	103.57	83.14	-	0.80	3.73	1.17
**$HfS_2/PbSe_2^{\dagger}$**	12.11	46.81	38.48	58.92	-34.69	-	-0.59	2.56	2.28
**HfS_2/SnO_2**	116.27	63.13	163.65	179.39	53.14	-	0.30	5.25	3.11
**MnO_2/CrS_2**	80.52	47.25	119.10	127.77	33.27	-	0.26	5.32	3.23
**MnO_2/GeS_2**	261.26/73.40	55.18/35.82	182.22/96.28	124.09(234.85)	80.82	49.78	0.65/0.34	8.84/5.19	3.26/2.97
**MnS_2/HfO_2**	52.66/220.56	30.30/53.93	76.93/173.33	207.23(91.98)	55.84	14.97	0.27/0.61	3.64/6.62	2.20/2.93
**MnS_2/MoO_2**	236.76	81.46	242.43	318.22	155.30	-	0.49	7.47	3.78
**$MnTe_2/PbSe_2^{\dagger}$**	16.21	20.52	36.23	36.73	-4.31	-	-0.12	2.11	1.58
**$MnTe_2/SnO_2$**	147.06	76.41	201.13	223.47	70.65	-	0.32	5.23	3.06
**MoS_2/GeO_2**	84.67/336.59	17.47/20.66	57.94/77.88	186.21(138.53)	122.50	53.47	0.66/0.88	4.38/8.20	1.81/1.97
**$MoS_2/HfSe_2$**	74.21	34.87	94.89	109.08	39.34	-	0.36	3.68	2.08
**MoS_2/SiO_2**	178.22	89.85	238.94	268.07	88.36	-	0.33	7.31	4.23
**$MoSe_2/GeO_2$**	138.62	77.20	198.34	215.82	61.42	-	0.28	5.45	3.26
**$MoSe_2/SiTe_2^{\dagger}$**	12.82	61.65	42.46	74.47	-48.82	-	-0.66	2.86	2.60
**$MoTe_2/HfSe_2$**	117.59	9.54	35.29	127.13	108.05	-	0.85	3.50	0.96
**PbO_2/HfS_2**	132.64	61.12	167.36	193.76	71.51	-	0.37	4.98	2.80
**PbO_2/MnS_2**	132.53	51.80	148.97	184.33	80.73	-	0.44	5.31	2.81
**PbO_2/MoS_2**	138.95	75.08	194.97	214.03	63.88	-	0.30	5.47	3.24
**$PbTe_2/WSe_2$**	143.36	93.52	226.39	236.88	49.84	-	0.21	4.06	2.55
**SiO_2/MnS_2**	159.05	63.07	180.65	222.12	95.99	-	0.43	7.21	3.84
**SiO_2/MoS_2**	202.91	116.69	296.35	319.60	86.21	-	0.27	8.08	4.88
**SiO_2/PbS_2**	87.52	51.39	129.52	138.92	36.13	-	0.26	4.23	2.57
**SiO_2/WS_2**	206.76	126.44	313.84	333.20	80.32	-	0.24	7.00	4.31
**$SiSe_2/HfO_2$**	171.18	72.92	204.54	244.10	98.26	-	0.40	5.98	3.27
**SnO_2/GeS_2**	139.40	74.95	194.98	214.36	64.45	-	0.30	64.45	
**SnO_2/MnS_2**	153.30	70.10	192.41	223.40	83.20	-	0.37	6.58	3.69
**$SnTe_2/HfSe_2$**	8.95	0.69	2.58	9.64	8.26	-	0.86	1.02	0.27
**$SnTe_2/GeSe_2$**	20.01	6.30	19.16	26.31	13.71	-	0.52	1.83	0.90
**$SnTe_2/MnS_2$**	177.75	46.75	148.05	224.50	131.01	-	0.58	4.88	2.23
**$SnTe_2/SiS_2$**	182.40	94.48	248.96	276.87	87.92	-	0.32	5.72	3.34
**$SnTe_2/WO_2^{\dagger}$**	67.16	100.75	161.20	167.92	-33.59	-	-0.20	3.69	2.86
**SnO_2/HfS_2**	147.35	66.29	182.89	213.64	81.06	-	0.38	5.65	3.15
**$SnO_2/GeSe_2$**	123.63	42.27	126.00	165.80	81.36	-	0.49	4.89	2.47
**TiO_2/GeS_2**	84.73	28.47	85.25	113.20	56.25	-	0.50	5.33	2.68
**TiO_2/MoS_2**	158.34	104.23	251.42	262.57	54.11	-	0.21	7.33	4.62
**TiO_2/MnS_2**	130.09	73.64	188.09	203.73	56.45	-	0.28	7.01	4.22
**TiO_2/WS_2**	173.40	117.08	279.56	290.48	56.33	-	0.19	6.60	4.19
**$TiSe_2/SiS_2$**	70.96/89.77	47.61/55.39	113.97/137.02	143.73(119.54)	28.31	37.59	0.22	4.99/5.52	3.16/3.41
**$TiTe_2/SnSe_2$**	62.20	40.12	97.56	102.32	22.09	-	0.22	3.66	2.29
**$TiSe_2/MoS_2$**	114.42	28.64	91.63	143.06	85.78	-	0.60	4.64	2.07
**WO_2/SiS_2**	150.15	72.16	195.77	222.76	77.54	-	0.35	6.04	3.45
**$WO_2/MoSe_2$**	152.19	76.71	204.01	228.90	75.47	-	0.33	4.94	2.86
**WS_2/PbO_2**	87.31	77.23	163.92	164.54	10.09	-	0.06	4.30	2.95
**$WS_2/SiTe_2$**	128.75	60.50	164.63	189.24	68.25	-	0.36	4.53	2.56
**WSe_2/GeO_2**	157.37	96.96	239.98	254.33	60.41	-	0.24	5.30	3.27
**WSe_2/MoS_2**	149.90	99.57	239.31	249.47	50.34	-	0.20	5.15	3.25
**WSe_2/SnS_2**	104.98	40.78	117.48	145.76	64.20	-	0.44	4.22	2.23

Continued on next page

TABLE S4 – Continued from previous page

Material	K	G	Y^{2D}	$C_{11}(C_{22})$	C_{12}	C_{66}	ν	V_l	V_t
**WSe_2/PbS_2**	18.10	1.64	6.01	19.73	16.46	-	0.83	1.46	0.42
**WTe_2/HfS_2**	142.41	73.32	193.60	215.73	69.09	-	0.32	4.48	2.61
**$WTe_2/PbSe_2$**†	33.67	57.65	85.02	91.32	-23.98	-	-0.26	2.72	2.16
**$WTe_2/TiSe_2$**	125.74	7.50	28.31	133.24	118.24	-	0.89	3.60	0.85
**$WTe_2/SnSe_2$**	73.82	41.39	106.08	115.21	32.43	-	0.28	3.45	2.07
**$WS_2/TiSe_2$**	94.95	46.17	124.26	141.12	48.78	-	0.35	4.23	2.42
**WS_2/SnO_2**	84.75	48.06	122.68	132.81	36.70	-	0.28	4.11	2.47
			**Heterostructure with two planar (P) monolayer**						
**BaOCdS**	28.00	0.88	3.40	28.88	27.12	-	0.94	3.03	0.53
**BaOMgS**†	0.63	1.41	1.73	2.03	-0.78	-	-0.38	0.94	0.78
**BaOZnSe**	30.43	9.84	29.73	40.27	20.60	-	0.51	3.52	1.74
**BaOCdSe**	17.01	0.78	2.99	17.79	16.23	-	0.91	2.25	0.47
**BaSSrTe**	2.27	0.24	0.88	2.51	2.02	-	0.81	0.93	0.29
**BaSZnTe**	20.94	0.96	3.66	21.89	19.98	-	0.91	2.59	0.54
**BaSeBeTe**	23.46	9.57	27.20	33.03	13.89	-	0.42	2.98	1.61
**BaSeCdS**	26.13	18.11	42.79	44.24	8.01	-	0.18	3.55	2.27
**BaSeMgTe**	13.00	11.30	24.18	24.30	1.70	-	0.07	2.82	1.92
**BaTeCdS**	8.20	1.14	4.02	9.35	7.06	-	0.76	1.80	0.63
**BeSBaSe**†	7.75	12.77	19.30	20.52	-5.02	-	-0.24	2.45	1.93
**BeSCdO**	9.64	7.75	17.18	17.39	1.89	-	0.11	2.65	1.77
**BeSMgTe**	39.83	32.87	72.04	72.70	6.95	-	0.10	4.80	3.22
**BeSMgO**	70.34	16.84	54.36	87.19	53.50	-	0.61	8.16	3.59
**BeSeBaS**	1.37	0.24	0.81	1.60	1.13	-	0.70	0.71	0.27
**BeSZnO**	35.13	19.48	50.12	54.60	15.65	-	0.29	5.27	3.15
**BeSeCdO**	40.76	12.24	37.66	53.00	28.52	-	0.54	4.17	2.00
**BeSeMgO**	20.10	18.63	38.67	38.73	1.47	-	0.04	4.45	3.09
**BeSeZnTe**	24.21	6.00	19.23	30.21	18.21	-	0.60	2.92	1.30
**BeTeBaO**†	5.35	13.17	15.21	18.52	-7.82	-	-0.42	2.39	2.01
**BeTeZnS**	33.15	4.65	16.33	37.80	28.49	-	0.75	3.61	1.27
**CdOZnS**	39.18	15.54	44.50	54.72	23.65	-	0.43	4.26	2.27
**CdOBeTe**	19.25	0.92	3.51	20.17	18.33	-	0.91	2.41	0.52
**CdSBaO**	6.79	4.98	11.49	11.77	1.82	-	0.15	1.94	1.26
**CdSBaTe**	14.41	1.59	5.73	16.00	12.82	-	0.80	2.35	0.74
**CdSMgTe**†	12.51	29.34	35.08	41.85	-16.83	-	-0.40	3.74	3.13
**CdSeMgO**	29.90	2.84	10.39	32.74	27.05	-	0.83	3.02	0.89
**CdTeBeSe**	42.88	6.78	23.42	49.66	36.10	-	0.73	3.29	1.22
**CdTeMgO**	38.00	22.71	56.85	60.71	15.30	-	0.25	3.61	2.21
**CdTeZnO**	24.36	23.62	47.97	47.98	0.74	-	0.02	3.01	2.11
**CdTeZnS**	3.38	0.91	2.86	4.28	2.47	-	0.58	1.06	0.49
**MgOBeSe**	55.45	18.25	54.92	73.70	37.21	-	0.50	6.14	3.06
**MgOCdTe**	45.98	18.08	51.91	64.06	27.90	-	0.44	3.71	1.97
**MgSBaO**	7.62	5.35	12.57	12.97	2.28	-	0.18	2.38	1.53
**MgSBeSe**	24.76	9.97	28.44	34.74	14.79	-	0.43	4.33	2.32
**MgSZnO**	11.85	10.12	21.84	21.97	1.73	-	0.08	3.44	2.34
**MgSeBaO**	27.15	7.34	23.12	34.49	19.81	-	0.57	3.59	1.65
**MgSeBeS**†	5.73	27.46	18.95	33.18	-21.73	-	-0.65	4.09	3.72
**MgSeSrTe**†	1.56	3.12	4.16	4.68	-1.56	-	-0.33	1.24	1.01
**MgSeZnTe**†	21.38	30.49	50.27	51.87	-9.11	-	-0.18	4.12	3.16
**MgTeBeS**	44.84	28.58	69.82	73.42	16.26	-	0.22	4.89	3.05
**MgTeCdSe**†	8.43	26.20	25.51	34.63	-17.77	-	-0.51	3.26	2.84
**MgTeZnO**†	6.04	24.02	19.31	30.06	-17.98	-	-0.60	2.83	2.53
**MgTeZnSe**†	0.87	8.09	3.13	8.96	-7.23	-	-0.81	1.69	1.60
**SrOBeSe**	15.84	8.56	22.24	24.41	7.28	-	0.30	3.16	1.87
**SrOMgS**	32.78	7.37	24.07	40.15	25.41	-	0.63	4.64	1.99
**SrOZnS**	26.41	8.35	25.38	34.76	18.06	-	0.52	3.77	1.85
**SrOZnSe**	25.42	5.85	19.02	31.27	19.58	-	0.63	3.29	1.42
**SrSBaSe**	1.24	0.09	0.33	1.33	1.15	-	0.87	0.71	0.18
**SrSCdTe**†	15.13	26.50	38.53	41.64	-11.37	-	-0.27	3.68	2.93
**SrSMgSe**†	13.57	15.52	28.96	29.09	-1.94	-	-0.07	3.72	2.72
**SrSeBeO**†	18.28	48.64	53.15	66.92	-30.36	-	-0.45	4.51	3.84

Continued on next page

TABLE S4 – Continued from previous page

Material	K	G	Y^{2D}	$C_{11}(C_{22})$	C_{12}	C_{66}	ν	V_l	V_t
**SrSeMgS**†	0.49	14.87	1.90	15.36	-14.38	-	-0.94	2.57	2.53
**SrSeZnS**	17.46	6.25	18.41	23.72	11.21	-	0.47	2.83	1.45
**SrSeZnTe**†	9.23	11.95	20.83	21.17	-2.72	-	-0.13	2.54	1.91
**SrTeBaO**	50.55	17.53	52.06	68.07	33.02	-	0.49	4.37	2.22
**SrTeCdS**	19.24	11.29	28.46	30.53	7.95	-	0.26	2.91	1.77
**SrTeMgO**	12.36	1.31	4.75	13.68	11.05	-	0.81	1.88	0.58
**SrTeZnS**	14.83	0.02	0.10	14.85	14.81	-	1.00	2.43	0.10
**ZnOBaSe**	23.72	1.77	6.59	25.50	21.95	-	0.86	3.06	0.81
**ZnOBeS**	47.36	14.22	43.74	61.58	33.14	-	0.54	5.60	2.69
**ZnOCdTe**	49.20	9.13	30.80	58.33	40.07	-	0.69	3.31	1.31
**ZnOMgTe**	21.20	15.83	36.25	37.03	5.37	-	0.15	3.14	2.05
**ZnSBaO**	22.58	17.84	39.86	40.42	4.74	-	0.12	3.73	2.48
**ZnSBeSe**	18.94	0.24	0.96	19.18	18.69	-	0.97	2.76	0.31
**ZnSCdO**	28.82	0.02	0.09	28.85	28.80	-	1.00	3.09	0.09
**ZnSMgSe**	33.28	5.94	20.16	39.22	27.34	-	0.70	4.06	1.58
**ZnSSrO**	11.80	9.35	20.86	21.14	2.45	-	0.12	2.94	1.95
**ZnSeBaS**	34.69	2.31	8.67	37.00	32.38	-	0.88	3.42	0.85
**ZnSeBeO**	19.35	8.11	22.85	27.46	11.24	-	0.41	3.42	1.86
**ZnSeCdTe**	4.71	1.81	5.23	6.52	2.90	-	0.44	1.27	0.67
**ZnSeSrO**†	0.14	8.34	0.53	8.47	-8.20	-	-0.97	1.71	1.70
**ZnTeBaS**†	9.40	21.38	26.12	30.78	-11.97	-	-0.39	3.07	2.56
**ZnTeBeS**†	14.29	19.44	32.95	33.74	-5.15	-	-0.15	3.06	2.32
**ZnTeMgS**	28.71	6.74	21.83	35.45	21.98	-	0.62	3.61	1.57
**ZnTeSrO**	69.64	12.67	42.87	82.31	56.97	-	0.69	5.00	1.96

* che218@lehigh.edu

[1] G. Kresse and J. Furthmüller, Comput. Mater. Sci. **6**(1), 15–50 (1996).

[2] A. Marmier, Z. A. Lethbridge, R. I. Walton, C. W. Smith, S. C. Parker, and K. E. Evans, Computer Physics Communications **181**(12), 2102–2115, ISSN 0010-4655 (2010).

[3] P. P. R. Gaillac1 and F. X. Coudert, J. Phys.: Condens. Mat. **28**, 275201 (2016).

[4] .

[5] Z.-L. Liu, C. E. Ekuma, W.-Q. Li, J.-Q. Yang, and X.J. Li *ElasTool: an automated toolkit for elastic constants calculation* Computer Physics Communications **270**, 108180 (2022).
